# Enantioselective Thiolysis and Aminolysis of Cyclic Anhydrides Using a Chiral Diamine-Derived Thiourea Catalyst

**DOI:** 10.1021/acsomega.1c04741

**Published:** 2021-11-24

**Authors:** Jae Ho Shim, Sung Joo Park, Byung Kook Ahn, Ji Yeon Lee, Hyeon Soo Kim, Deok-Chan Ha

**Affiliations:** † Department of Anatomy, Korea University College of Medicine, 46, Gaeunsa 2-gil, Seongbuk-gu, Seoul 02842, Republic of Korea; ‡ Department of Chemistry, Korea University, 145 Anam-ro, Seongbuk-gu, Seoul 02841, Korea

## Abstract

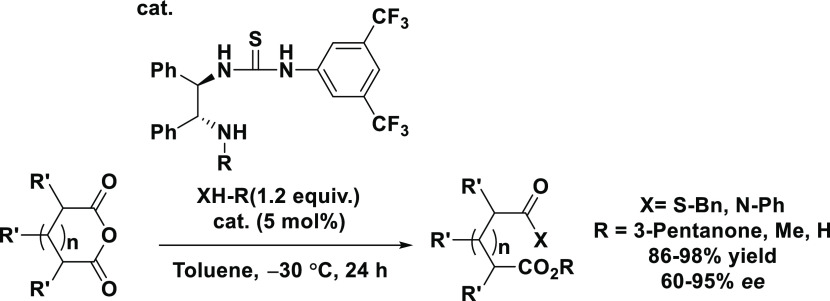

Catalytic desymmetrization of cyclic anhydrides has been widely investigated in the field of organocatalysis. Using this approach, many stereocenters can be established in a single, symmetry-breaking transformation. Herein, a thiourea organocatalyst was prepared in a single step from a chiral diamine, (*R*,*R*)-1,2-diphenylethylenediamine, and used for the desymmetrization of various cyclic anhydrides through double hydrogen-bonding activation. The asymmetric ring-opening reaction of the cyclic anhydride proceeded via the enantioselective addition reaction catalyzed by diamine thiourea. Thiolysis afforded the desired products in the yields of 86–98% and enantioselectivities of 60–94%, while aminolysis afforded the yields of 90–94% and enantioselectivities of 90–95%.

## Introduction

**Scheme 1:**
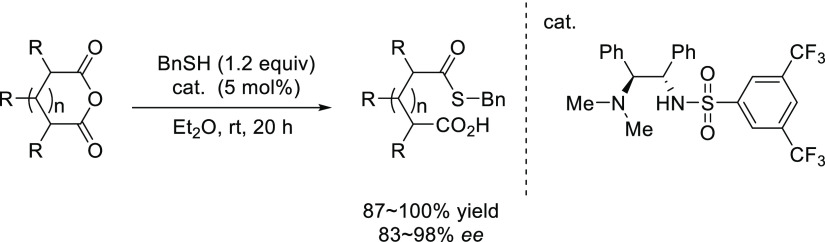
Thiolysis of a Prochiral Cyclic Anhydride Using a Sulfonamide Organocatalyst

An organocatalyst is composed of carbon, hydrogen, sulfur, and other nonmetallic elements commonly found in organic molecules. Organocatalysts are used to catalyze organic reactions. Unlike conventional catalysts, organic catalysts do not possess a metal and a ligand. Since 1998, many studies have been conducted on stereoselective syntheses using organic catalysts that are devoid of metals.^1^ Among them, the asymmetric ring-opening reaction of cyclic meso-anhydrides is particularly useful for the synthesis of biologically active substances. In this context, mesochiral, prochiral, and racemic cyclic anhydrides have been used as synthetic building blocks of natural products containing α-amino acid, α-hydroxy acid, and hemiester moieties. Hence, cyclic anhydrides are important building blocks for synthesis in the field of organocatalysis.^2^ In 1985, Oda studied the methanolysis of cyclic anhydrides using a readily available, stable, and inexpensive cinchona alkaloid as a chiral Lewis base and obtained product yields of up to 95% and enantioselectivities of 70% ee using a 10 mol % cinchonine catalyst.^3^ The effects of the structure and selectivity of the catalyst have also been examined; the results showed that the ring-opening reaction was dependent on a specific bond between the catalyst and the substrate.^4^ This research paved the way for many studies on asymmetric ring-opening reactions using organic catalysts. Although Oda’s work was limited to mono- or bicyclic anhydrides, Aitken extended this study to more complex multiring anhydrides.^5^ In 1993, Fujisawa studied asymmetric ring-opening reactions of cyclic anhydrides using diethyl zinc and cinchonidine as a catalyst to achieve enantioselectivities of up to 91% ee and yields of up to 57%, which, however, were deemed too low relative to the amount of the catalyst used.^6^ The asymmetric ring-opening reaction was further developed by Bolm, affording products of up to 99% yield and enantioselectivities of up to 99% ee using excess methanol and 110 mol % quinine or quinidine at low temperatures.^7^ Various bicyclic and tricyclic anhydrides were subjected to methanolysis under the same conditions, and very good yields of 65–90% as well as enantioselectivities were achieved; however, the reaction time was too long despite the use of excess amount of catalyst. Deng carried out an asymmetric ring-opening reaction using a bis-cinchona alkaloid catalyst to afford the product in excellent yield and enantioselectivity.^8^ This was achieved at a relatively low temperature, using a significantly less Sharpless catalyst (5 mol %) compared to the aforementioned reaction. In 2005, Nagao carried out a thiolysis reaction of a prochiral cyclic anhydride, using a chiral sulfonamide organocatalyst (Scheme 1).^9^

This thiolysis reaction was the first reaction in which a thiol was used as a nucleophile; the carbonyl of the anhydride is activated by the acidic hydrogen of the sulfonamide, which results in excellent reactivity and enantioselectivity. Nagao used (*R*,*R*)-1,2-diphenylethylenediamine (DPEN) as the basic skeleton of the chiral catalyst within which thiourea is introduced for application in the asymmetric ring-opening reaction by hydrogen-bonding catalysis.^10,11^ We envisaged that we could use this catalyst for investigating the thiolysis of a range of cyclic anhydrides.

## Results and Discussion

**Scheme 2:**
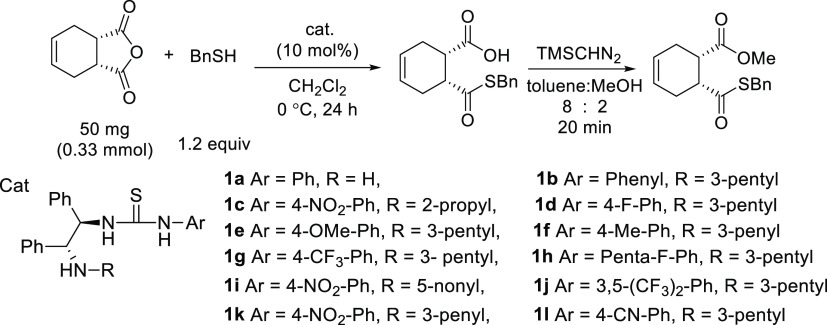
Asymmetric Ring-Opening Reaction of *cis*-1,2,3,6-Tetrahydrophthalic Anhydride Using a Thiourea Catalyst with Various Substituents

In the present study, a thiourea molecule possessing a chiral diamine DPEN skeleton was used to catalyze the asymmetric ring-opening reaction of cyclic meso-anhydrides and the aminolysis reaction of cyclic anhydrides. The catalyst was investigated through the variations illustrated in Scheme 2. As the reaction using monothiourea resulted in a low yield, *N*-monoalkylated thiourea was used to increase the basicity of the catalyst (R substituent). In addition, the ability of thiourea to form hydrogen bonds was enhanced by increasing the acidity of hydrogen on thiourea (Ar substituent); this was achieved by introducing an electron-withdrawing group (EWG). The results of the asymmetric ring-opening reaction of *cis*-1,2,3,6-tetrahydrophthalic anhydride, with respect to changes in the substitution pattern on the thiourea catalyst, are summarized in Table 1.

**Table 1:** Effects of Different Catalyst Substituents on the Yield of the Ring-Opening Reaction and ee of the Products

entry	catalyst	solvent	yield (%)^a^	ee (%)^b^
1	1a	CH_2_Cl_2_	80	0
2	1b	CH_2_Cl_2_	83	36
3	1c	CH_2_Cl_2_	88	57
4	1d	CH_2_Cl_2_	89	70
5	1e	CH_2_Cl_2_	89	49
6	1f	CH_2_Cl_2_	88	70
7	1g	CH_2_Cl_2_	82	72
8	1h	CH_2_Cl_2_	94	63
9	1i	CH_2_Cl_2_	92	58
10	1j	CH_2_Cl_2_	89	73
11	1k	CH_2_Cl_2_	87	67
12	1l	CH_2_Cl_2_	87	65
13	1j	hexane	86	67
14	1j	diethyl ether	82	65
15	1j	THF	81	64
16	1j	toluene	98	74

a Isolated yield of *S*-benzyl thioester monocarboxylic acid.

b The ee values were determined by chiral-phase HPLC using the OD-H column.

The results demonstrated that a higher enantioselectivity was achieved when 3-pentyl (entry 2) or 2-propyl (entry 3) was the alkyl group than that obtained when no alkyl group was used (entry 1). An alkyl group substituted on one amine exerts a significant influence on the enantioselectivity. In addition, a better enantioselectivity was observed when the aryl group on the thiourea moiety contained an EWG rather than an electron-donating group (EDG). This is because the thiourea hydrogen involved in hydrogen bonding is more acidic when an EWG rather than an EDG is substituted, making hydrogen bonding easier, thereby affecting the enantioselectivity.

Having established that the most effective thiourea catalyst was substituted with 3,5-(CF_3_)_2_-Ph (entry 10), further optimization studies were conducted in which the reaction solvent was examined. Compared to CH_2_Cl_2_ (entry 10) and toluene (entry 16), THF (entry 15) and Et_2_O (entry 14) displayed lower yields and enantioselectivities. This result showed that in the case of noncovalent catalysis, the enantioselectivity was lower in solvents that can participate in hydrogen bonding. This accounted for the higher enantioselectivity observed with CH_2_Cl_2_ in noncovalent organic catalysis and even better reactivity and selectivity with toluene.

**Scheme 3:**
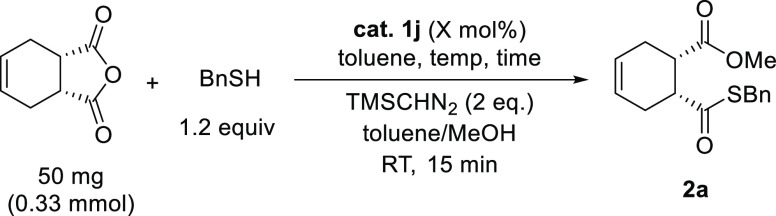
Thiolysis under Optimized Reaction Conditions

Thus far, the highest enantioselectivity was achieved when thiourea substituted with 3,5-(CF_3_)_2_-Ph was used as the catalyst and toluene was used as the solvent (Scheme 3). Subsequently, the optimal reaction conditions were established with respect to the temperature, reaction time, and mole fraction of the catalyst (Table 2). Initially, the reaction temperature was investigated (entries 2–5). The results showed that when the temperature was decreased to 0 °C, the product was obtained in a comparable yield and with improved enantioselectivity (entry 2). Thereafter, further decreasing the temperature was found to be detrimental, both in terms of the yields and enantioselectivities of the reaction. Therefore, subsequent reactions were conducted at 0 °C. Although the highest yield was obtained when the reaction was carried out for 96 h in toluene, it did not have a significant effect on the enantioselectivity; thus, longer reaction times were not warranted. The optimal catalyst loading was 5 mol %, as a decrease in the amount of catalyst to 2.5 or 1 mol % saw a drastic drop in both the yields and enantioselectivities of the products. Therefore, the reaction has the following optimal conditions: temperature, 0 °C; time, 24 h; catalyst loading, 5 mol %; solvent, toluene.

**Table 2:** Optimization of the Temperature, Reaction Time, and Mole Fraction

entry	catalyst (mol %)	time (h)	temp (°C)	yield (%)^a^	ee (%)^b^
1	5	24	rt	94	74
2	5	24	0	92	81
3	5	24	–20	61	78
4	5	24	–48	53	65
5	5	24	–78	77	45
6	5	96	0	94	80
7	2.5	96	0	69	74
8	1	96	0	30	65

a Isolated yield of *S*-benzyl thioester monocarboxylic acid.

b The ee values were determined by chiral-phase HPLC using the OD-H column.

**Scheme 4:**
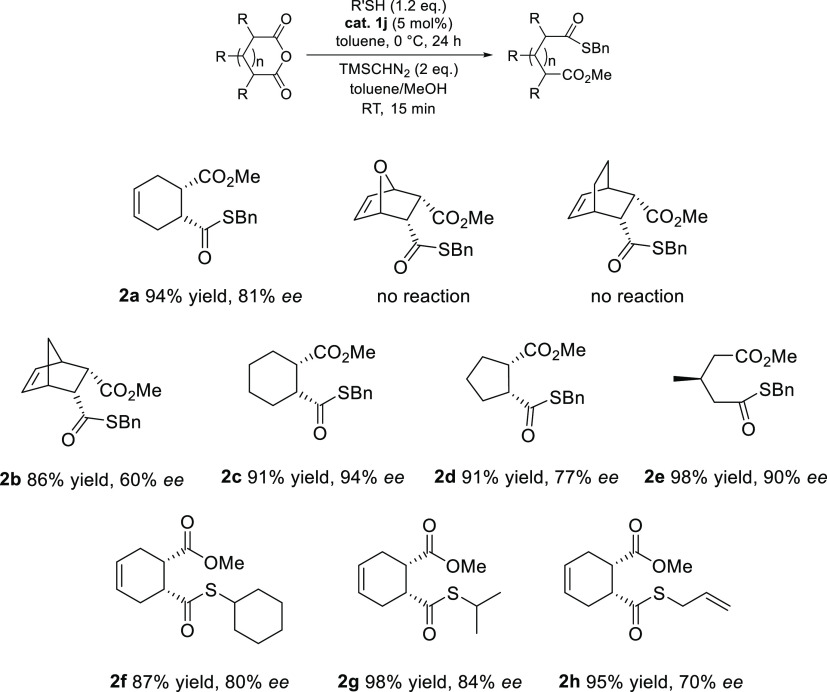
Ring-Opening Thiolysis Using Various Thiols and Anhydrides

**Scheme 5:**
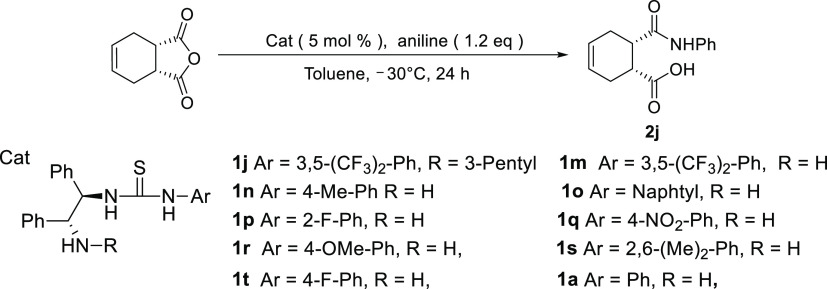
Asymmetric Ring-Opening Aminolysis of an Anhydride Using a Thiourea Catalyst with Various Substituents and Aniline

In the previous experiment (Scheme 4), the optimal conditions were established for the ring-opening reaction of *cis*-1,2,3,6-tetrahydrophthalic anhydride using benzyl mercaptan and the thiourea catalyst. Using these optimized conditions, the substrate scope of the reaction was investigated by employing various anhydrides and thiols (Scheme 4).^12^ With bicyclic anhydrides, the products were afforded in high yields and enantioselectivities. However, for tricyclic anhydrides, the reaction either did not proceed or the yields and selectivities were inferior to those of the bicyclic anhydrides (Schemes 4 and 2b). In addition, reactions were carried out using *cis*-1,2,3,6-tetrahydrophthalic anhydride with various thiols (Schemes 4 and 2f–i). Better enantioselectivities were observed with aliphatic thiols compared to that obtained with aromatic thiols. The functional groups on the thiourea catalyst were once again examined, this time with respect to the reaction between a cyclic anhydride and aniline. The reactions were carried out with relatively morphologically fixed tetrahydrophthalic anhydride and aniline in the presence of the thiourea catalyst bearing a range of substituents (Scheme 5). The results are summarized in Table 3.

**Table 3:** Effect of Different Catalyst Substituents on the Yield of the Ring-Opening Reaction and ee of the Products

entry	catalyst	solvent	temp (°C)	time (h)	yield (%)^a^	head (%)^b^^,^^c^
1	1j	toluene	–30	24	87	30
2	1m	toluene	–30	24	98	92
3	1o	toluene	–30	24	87	30
4	1n	toluene	–30	24	82	13
5	1o	toluene	–30	24	83	6
6	1o	toluene	–30	24	83	33
7	1p	toluene	–30	24	87	30
8	1q	toluene	–30	24	85	17
9	1r	toluene	–30	24	87	6
10	1s	toluene	–30	24	87	60
11	1a	toluene	–30	24	90	12
12	1t	toluene	–30	24	90	25
13	1m	CH_2_Cl_2_	–30	24	90	69
14	1m	hexane	–30	24	93	49
15	1m	toluene/CCl_4_	–30	24	84	27
16	1m	THF	–30	24	84	27
17	1m	diethyl ether	–30	24	85	29

a Isolated yield of products.

b Determined by GC using the Agilent HP-1 column (19091Z-413, 30 m × 0.32 mm × 0.25 μm); conditions: initial temp, 50 °C; initial time, 3 min; 25.0 °C/min; final temp, 280 °C; 17 psi; retention time, 10.76 14.12 min.

c Absolute configuration.^8^

The functional groups of the catalyst were categorized as electron-withdrawing or electron-donating, and the effect thereof on the stereoselectivity was examined. The best yield and enantioselectivity were obtained in the presence of electron-withdrawing fluorine in the 3,5-(CF_3_)_2_-Ph moiety of catalyst **1k** (entry 2). This example demonstrated the importance of the bond between the carbonyl groups of the catalyst and the cyclic anhydride. In stark contrast, a dramatic decrease in the stereoselectivity of the reaction was observed with the methyl- or methoxy-substituted catalysts (entries 3 and 7, respectively); it was speculated that this was due to the reduced polarity of the carbonyl group. Even in the case of an electron-attracting substituent, nitrogen itself may affect the hydrogen bond between the catalyst and the cyclic anhydride, resulting in reduced stereoselectivity. In addition, the position of the substituent seemed to have an effect on the stereoselectivity of the reaction. Fluorine in the para position showed better stereoselectivity compared to the ortho position. This could be attributed to the steric hindrance of the fluorine atom in the ortho position. The effect of several organic solvents on the enantioselectivity of the reaction was examined, and a high yield and the highest enantioselectivity were once again observed with the nonpolar solvent toluene. In addition, this reaction displayed high enantioselectivity only when it was carried out at a low temperature, as a dramatic decrease in the enantioselectivities was observed at room temperature and 0 °C (entries 18 and 19, respectively).

**Scheme 6:**
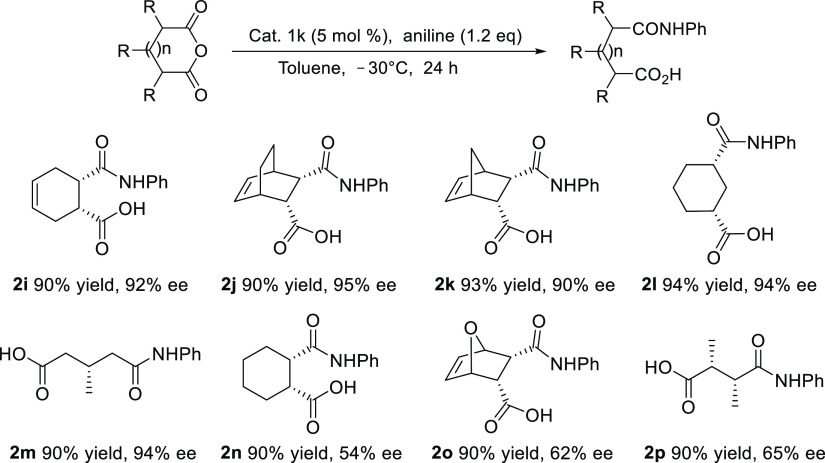
Ring-Opening Aminolysis Using Various Cyclic Anhydrides

Having established the optimal catalyst and conditions for the aminolysis reaction, asymmetric experiments of mesocyclic anhydrides such as single, double, and triple rings were then studied using the thiourea catalyst (Scheme 6).^13^ All products were obtained in excellent yields and enantioselectivities (Scheme 6). Slight variations in the enantioselectivities were attributed to the flexibility and ring size of the cyclic anhydrides. Flexible or large rings in the R portion interfered with the hydrogen bonds of the anhydride to the catalyst, resulting in reduced stereoselectivity. In addition, it was found that the presence of oxygen in the ring of cyclic anhydride (**2j**) reduced the stereoselectivity because it affected the hydrogen bond between the catalyst and the cyclic anhydride.

**Figure 1:**
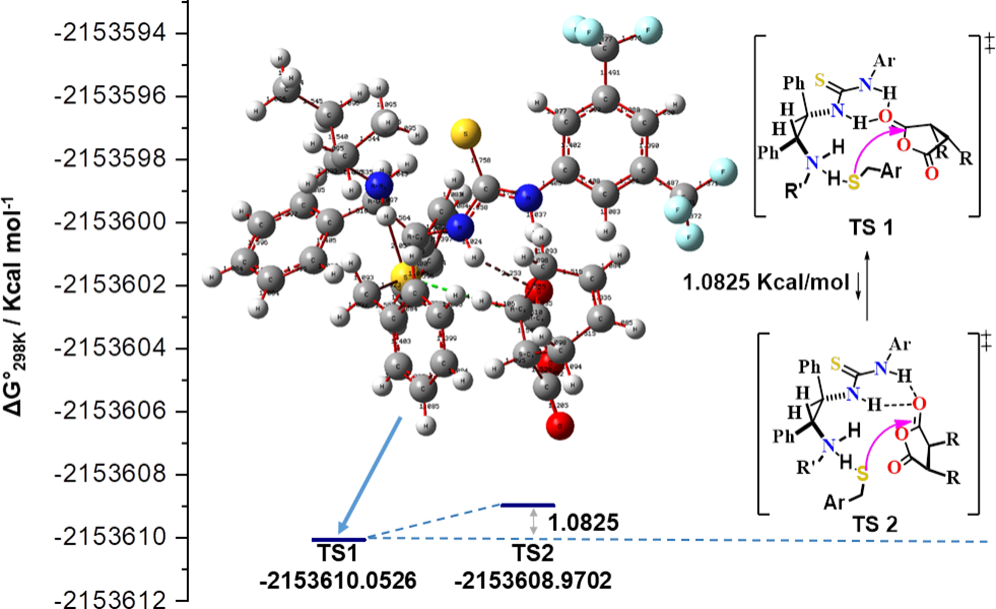
Proposed transition state for asymmetric addition using chiral (*R*,*R*)-1,2-diphenylethylenediamine-derived thiourea. B3LYP/6-31G(d,p)-calculated transition state of DPEN-thiourea-catalyzed enantioselective thiolysis. Transition-state (TS) structures for the C–S bond formation, through which TS 1 is possibly formed, are also shown.

**Scheme 7:**
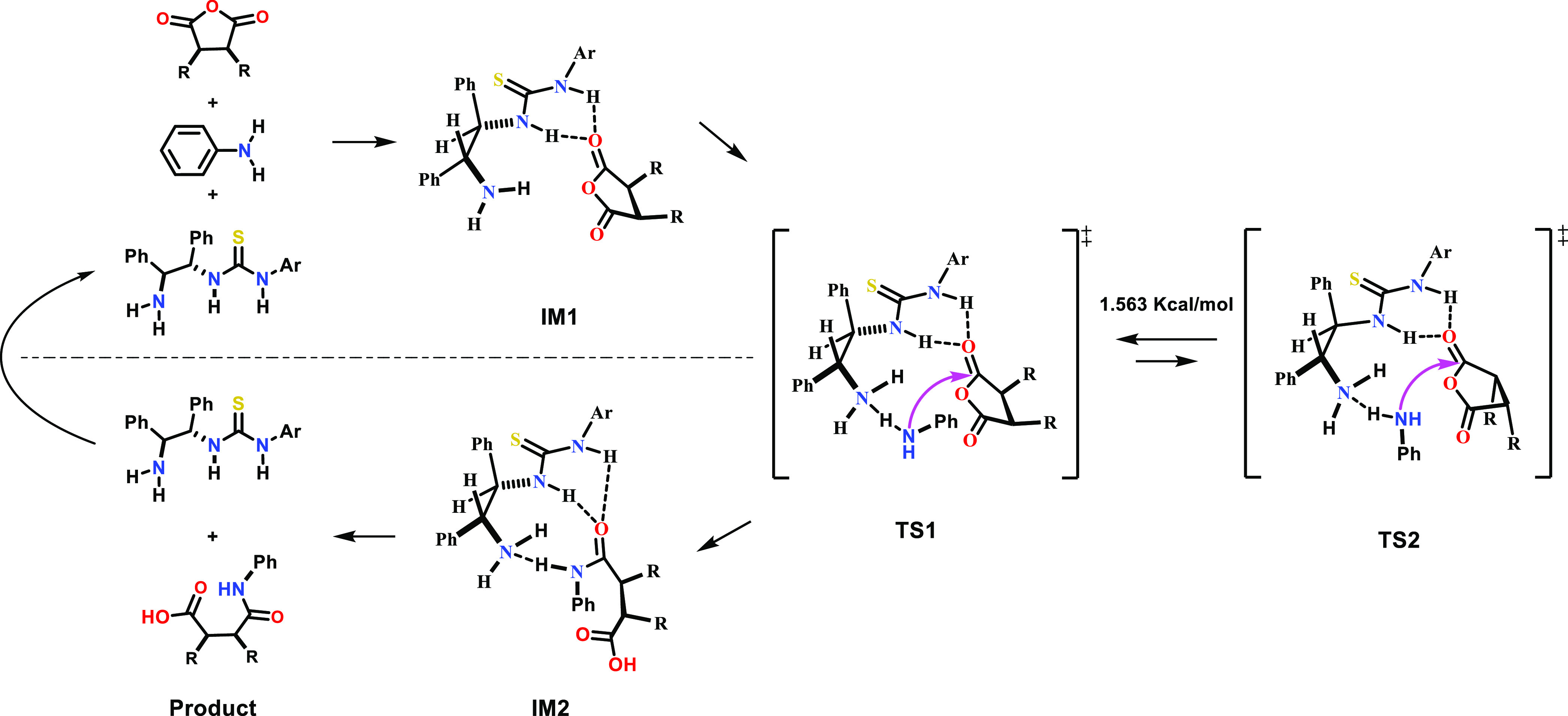
Proposed Reaction Mechanisms of Enantioselective Aminolysis

The following reaction mechanisms were proposed based on the results obtained from the thiolysis and aminolysis experiments (Figure 1), respectively: The formation of TS 2 would be difficult due to steric hindrance between the catalyst and R substituents of the anhydride. In the case of BnSH in TS 1, the reaction would proceed as stabilization may occur as a result of overlapping due to the neighboring σ* orbital when the nonbinding electrons of the thiol attack the π* orbitals of the carbonyl group. In this reaction, the transition state is thought to increase the reactivity of the electrophile by forming a double hydrogen bond with the hydrogen on the thiourea side of the catalyst and the oxygen of the cyclic anhydride, while a hydrogen bond forms between the alkylated amine and the thiol group, blocking the lower space. Here, the substituent of the hydrogen-bonded anhydride is positioned on the side where steric hindrance is relatively small and the nucleophile approaches upward, leading to the formation of products with high enantioselectivity (Scheme 7).

**Figure 2:**
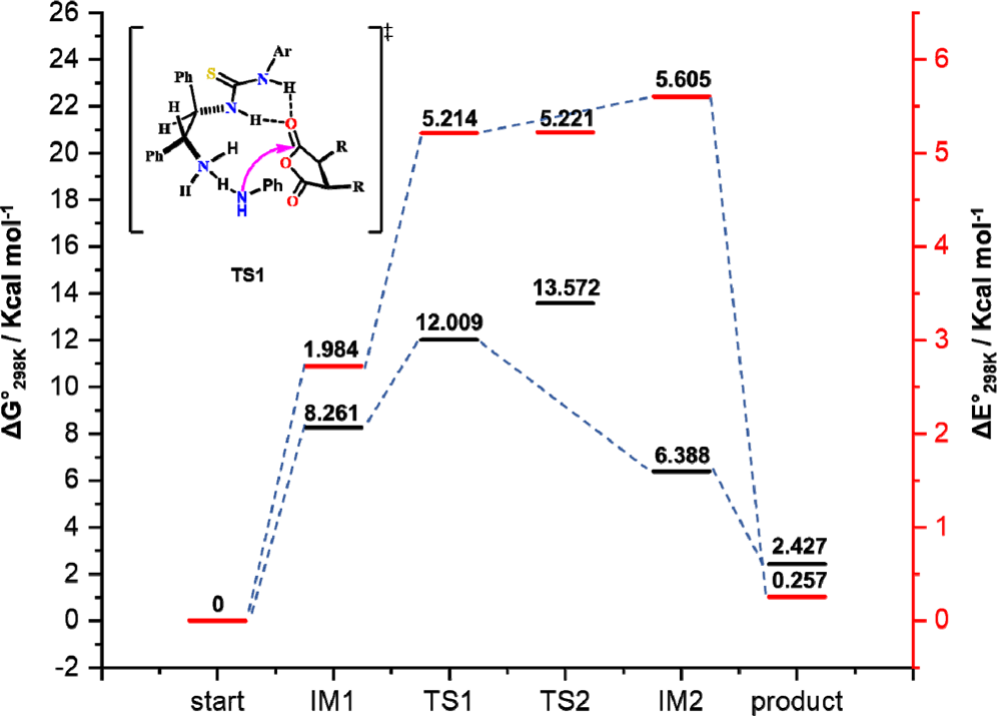
B3LYP/6-31G(d,p)-calculated transition state of the DPEN-thiourea-catalyzed enantioselective aminolysis. A comparison by the transition-state structures for the formation of C–N bonds from which major products can be formed is also shown.

TS 1 is an acceptable transition state because it would afford the expected product. In TS 2, the alkyl group of the anhydride is thought to sterically hinder the formation of a hydrogen bond between the catalyst and the anhydride. For TS 1, steric hindrance is thought to exist between the cyclic anhydride of the amine and the catalyst during the introduction of aniline. A comparison of the first and third transition states shows that it is in TS 1 that the LUMO can be stabilized by the σ orbital of the neighboring carbon when the entering nucleophile attacks the π* orbital of the carbonyl group; therefore, it was believed that the reactions would proceed via the TS 1 transition state (Figure 2).

**Figure 3:**
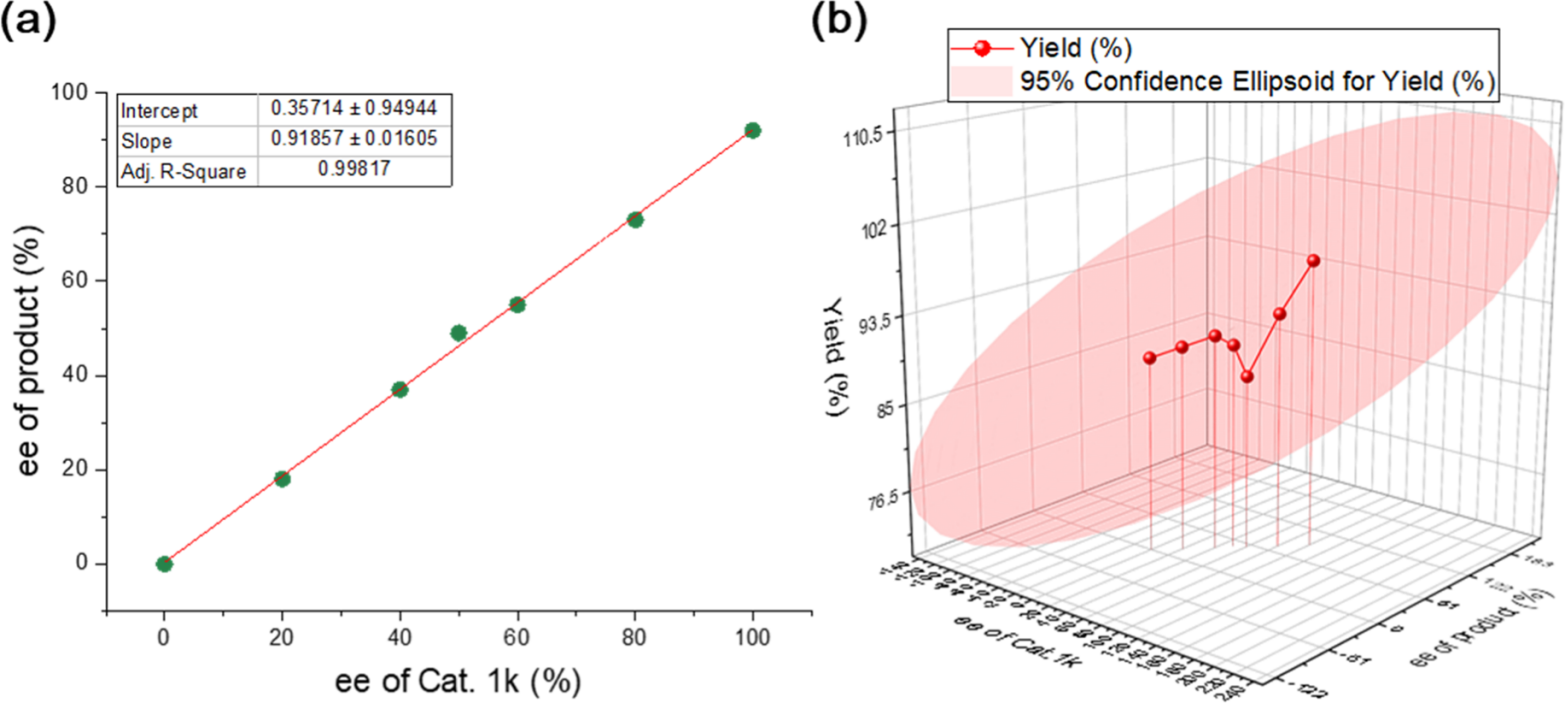
Relationship between (a) enantioselectivity and (b) yield of the aminolysis product and the catalyst.

**Scheme 8:**
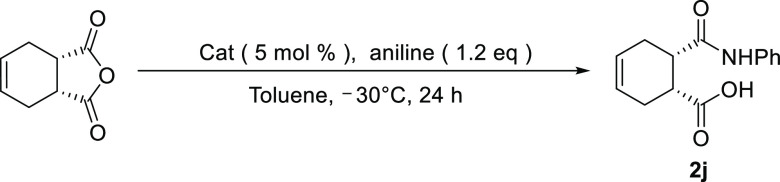
Aminolysis between Tetrahydrophthalic Anhydride and Aniline

The nonlinear effect experiment was carried out to establish the mechanism and the accompanying transition state of the reaction (Table 4). The reaction between aniline and tetrahydrophthalic anhydride using 5 mol % catalyst was examined (Scheme 8), and the results displayed an upward curve trend (Figure 3). We performed a nonlinear effect experiment to determine how the catalyst binds to the anhydride because the catalyst can form hydrogen bonds as monomers or dimers with the carbonyl groups of the anhydride. Based on the upward curves observed in the present study, it was confirmed that the catalyst acted as a monomer and precluded catalyst aggregation by showing no nonlinear effect (Table 4 and Figure 3).

**Table 4:** Nonlinear Effect Experiment

entry	1	2	3	4	5	6	7
ee of cat 1k (%)	0	20	40	50	60	80	100
ee of product (%)^b^	0	18	37	49	55	73	92
yield (5)^a^	89	90	91	90	87	93	98

a Yield of isolated products.

b Determined by the GC HP-1 column (30 m × 0.32 mm × 0.25 μm). Conditions: initial temperature, 50 °C; initial time, 3 min; 25.0 °C/min; final temperature, 280 °C; 17 psi; retention time, 10.76, 14.12 min.

**Figure 4:**
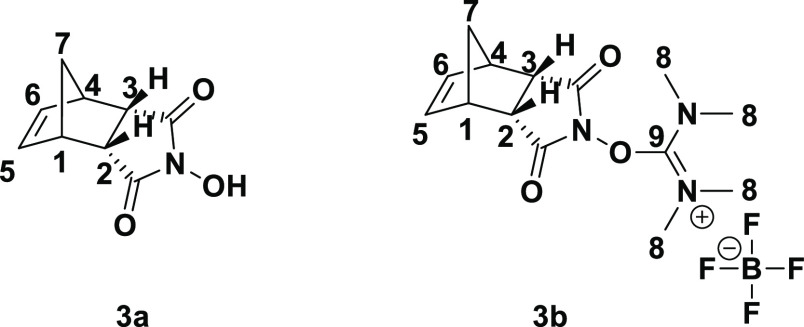
Numbering of *N*-hydroxy-5-endo-norbornene-2,3-dicarboximide (**3a**) and TNTU (**3b**).

**Scheme 9:**
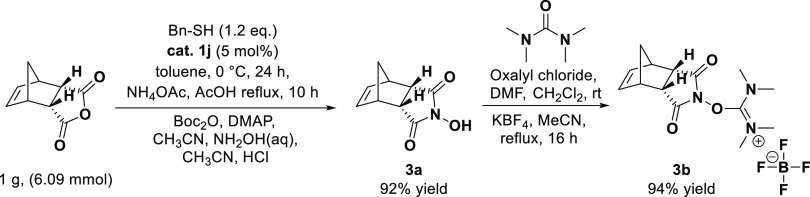
Reaction between Tetrahydrophthalic Anhydride and Aniline

As shown in Scheme 9, the transformation of the meso compound ring anhydride gram-scale synthesis was carried out through the set optimization conditions. The endo-isomer 3a of *N*-hydroxy-5-norbornene-2,3-dicarboximide has been obtained as a white solid according to the literature. The synthesis started from the commercially available exo- and endo-anhydrides using 1j catalyst. As a result of the synthesis, a yield of 92% was confirmed, and one step further, the peptide coupling reagent, tetramethyl-*O*-(*N*-succinimidyl)uronium tetrafluoroborate (TNTU), was synthesized as the final application compound. We compared the literature values of parameters for the exo- and endo compounds through NMR and quantum chemical calculations. For further evidence of the structures of **3a** and **3b,** DFT calculation spectra (^1^H, ^13^C NMR, and Tables S1 and S2) are shown. The results confirmed the suggested endo form structures. The *N*-hydroxy imides (**3a**) and TNTU (**3b**) present needed cross-peaks (Figure 4).

## Conclusions

Good yields and enantioselectivities were obtained in the enantioselective, organic, catalytic reaction between various cyclic anhydrides and thiols or aniline. In this process, the products of thiolysis and aminolysis were formed by using the monothiourea catalyst of (*R*,*R*)-(+)-diphenylethylenediamine (DPEN). Favorable reaction conditions were established using low catalyst loadings, relatively short reaction times, and low temperatures. In the case of the thiolysis reaction, the final compound was obtained in good yields and stereoselectivity in a single step, without the need for purification of any intermediate. In addition, it could be seen that high stereoselectivities were obtained by the double activation of hydrogen bonds between the anhydrides and thiourea, directed by steric factors. These exceptional results warrant further studies in the future to extend the use of this catalyst to various reactions.

## Experimental Section

### Synthesis of *N*-Mono Thiourea Catalyst

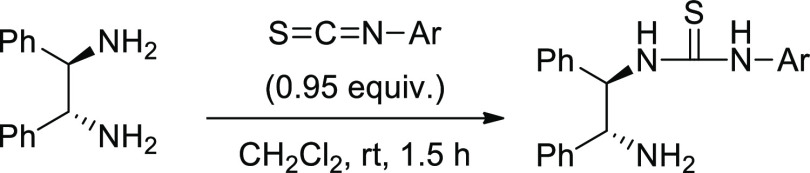



(*R*,*R*)-1,2-diphenylethylenediamine (DPEN, 1.0 equiv) was dissolved in CH_2_Cl_2_ (0.2 M) under argon. Isothiocyanate (0.95 equiv) was added, and the reaction mixture was stirred at room temperature. After 1.5 h, the reaction was terminated with water and extracted three times with CH_2_Cl_2_. The combined organic fractions were dried with anhydrous MgSO_4_, filtered, and concentrated under reduced pressure. The product was purified by column chromatography (SiO_2_, EtOAc/HX = 1:1).

### Synthesis of *N*-Monoalkylated Thiourea Catalyst

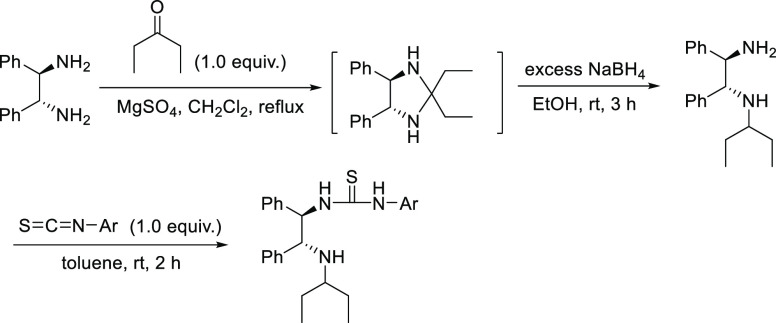



DPEN (1.0 equiv) was dissolved in CH_2_Cl_2_ (0.1 M), and MgSO_4_ and 3-pentanone (1.0 equiv) were added. The reaction mixture was heated to reflux for 48 h. CH_2_Cl_2_ was added and MgSO_4_ was filtered off, following which the solvent was removed under reduced pressure. The resulting diaminoacetal was dissolved in ethanol and excess NaBH_4_ was added, and the reaction mixture was stirred for 3 h at room temperature. After terminating the reaction with 1 N NaOH aqueous solution, the extraction was performed three times with CH_2_Cl_2_. The combined organic fractions were dried with anhydrous MgSO_4_, filtered, and the solvent was removed under reduced pressure. The product was purified by column chromatography (SiO_2_, CH_2_Cl_2_/MeOH/NH_3_ = 300:10:1). After dissolving the monoalkylated DPEN (1.0 equiv) in toluene (0.2 M) under argon, isothiocyanate (1.1 equiv) was added, and the reaction was stirred at room temperature for 2 h. The reaction mixture was added to water and extracted three times with 100 mL of CH_2_Cl_2_. The combined organic layers were dried with anhydrous MgSO_4_, filtered, and concentrated under reduced pressure. The product was purified by column chromatography (SiO_2_, EA/hexane = 1:5) to afford the desired product.

### Asymmetric Ring-Opening Reaction Using Thiourea Catalyst

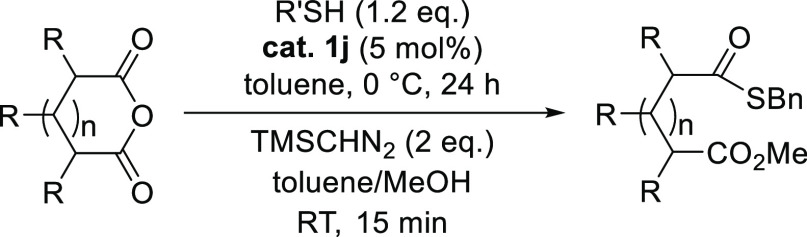



The cyclic meso-anhydride (0.33 mmol) and catalyst (5 mol %) were added to a reaction vessel at room temperature and then dissolved in toluene (0.2 M). The reaction vessel was placed in a thermostat set to 0 °C and stirred for 10 min before adding BnSH (1.2 equiv). After 24 h, the product was stirred with methanol (0.04 M) and TMSCHN_2_ (2.0 equiv). After 20 min, the residue was concentrated under reduced pressure and purified by column chromatography (SiO_2_, EA/HX = 1:10) to afford the purified product.

### Asymmetric Ring-Opening Reaction Using Chiral Thiourea Catalyst

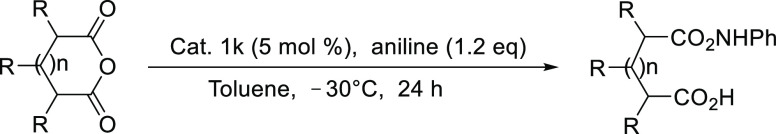



After dissolving the cyclic meso-anhydride (0.33 mmol, 50 mg) and catalyst (5 mol %, 6.5 mg) in toluene (2 mL) at −30 °C, aniline (1.2 equiv, 0.32 mL) was added. After 24 h, the reaction was terminated with 1 N HCl, and the extraction was performed with MC (methylene chloride). After the extraction, MgSO_4_ was added to dry the solution and then was removed by filtration. After the removal of the solvent under reduced pressure, column chromatography (230–400 mesh SiO_2_, CH_2_Cl_2_/methanol = 20:1) afforded the product in 90% yield.

### Methyl-(1*S*,6*R*)-6-((benzylthio)carbonyl)cyclohex-3-ene-1-carboxylate (**2a**)^12^

[α]_D_^25^ −0.116 (*c* 0.100, CHCl_3_); ^1^H NMR (300 MHz, CDCl_3_): 7.32–7.19 (m, 5H), 5.74–5.64 (m, 2H), 4.16 (d, *J* = 13.7 Hz, 1H), 4.10 (d, *J* = 13.7 Hz, 1H), 3.65 (s, 3H), 3.22–3.15 (m, 1H), 3.10–3.03 (m, 1H), 2.66–2.31 (m, 4H); ^13^C NMR (400 MHz, CDCl_3_): 200.07, 173.64, 137.75, 129.09, 128.80, 127.45, 125.81, 124.70, 52.07, 48.17, 40.43, 33.22, 26.45, 26.23; IR(KBr): 2919.8, 1702.9, 1438.7, 1247.8, 1207.3, 919.9, 709.7 cm^–1^; HRMS(FAB+) for C_16_H_18_O_3_S: [M + H]^+^ calcd 291.3904; found, 291.1055 cm^–1^, HPLC analysis (Chiralpak OD-H column, λ = 254 nm, hexane/*i*-PrOH = 95/5, flow rate 1.0 mL/min): *t*R = 9.3 min (major), 11.7 min (minor).

### Methyl-(1*S*,2*R*,3*S*,4*R*)-3-((benzylthio)carbonyl)bicyclo[2.2.1]hept-5-ene-2-carboxylate (**2b**)

[α]_D_^25^ −0.046 (*c* 0.100, CHCl_3_); ^1^H NMR (300 MHz, CDCl_3_): 7.41–7.20 (m, 5H), 6.52 (m, 1H), 6.01 (m, 1H), 4.12–3.97 (m, 2H), 3.62 (s, 1H) 3.47 (s, 3H), 3.19 (m, 1H), 1.58–1.25 (m, 5H); ^13^C NMR (400 MHz, CDCl_3_): 137.38, 133.01, 129.13, 128.79, 127.43, 56.71, 51.61, 49.62, 48.80, 45.93, 45.79, 33.60, 29.29; IR (KBr): 2977.7, 2372.1, 1741.5, 1454.1, 1338.4, 1180.3, 1020.2, 929.6, 721.3 cm^–1^; HRMS(FAB+) for C_17_H_18_O_3_S: [M + H]^+^ calcd 303.4015; found, 303.1055 cm^–1^, HPLC analysis (Chiralpak OD-H column, λ = 254 nm, hexane/*i*-PrOH = 95/5, flow rate 1.0 mL/min): *t*R = 10,1 min (major), 11.5 min (minor).

### Methyl-(1*S*,2*R*)-2-((benzylthio)carbonyl)cyclohexane-1-carboxylate (**2c**)^12^

[α]_D_^25^ −0.109 (*c* 0.100, CHCl_3_); ^1^H NMR (300 MHz, CDCl_3_): 7.36–7.18 (m, 5H), 4.09 (d, *J* = 2.4 Hz, 2H), 3.60 (s, 3H), 2.99–2.83 (m, 2H), 2.03–1.23 (m, 8H); ^13^C NMR (400 MHz, CDCl_3_): 201.69, 181.05, 137.72, 128.98, 128.84, 127.43, 53.07, 44.96, 33.26, 33.16, 33.39, 30.39, 29.27, 29.44, 25.34; IR(KBr): 2933.4, 1735.7, 1450.3, 1191.9, 962.4, 704.9 cm^–1^, HRMS(FAB+) for C_16_H_20_O_3_S: [M + H]^+^ calcd 293.4064; found, 293.1211; HPLC analysis (Chiralpak OD-H column, λ = 254 nm, hexane/*i*-PrOH = 95/5, flow rate 1.0 mL/min): *t*R = 8.0 min (major), 9.6 min (minor).

### Methyl (1*R*,2*S*)-2-((Benzylthio)carbonyl)cyclopentane-1-carboxylate (**2d**)

[α]_D_^25^ −0.149 (*c* 0.100, CHCl_3_); ^1^H NMR (300 MHz, CDCl_3_): 7.29–7.22 (m, 5H), 4.17–4.03 (q, *J* = 13.8 Hz, 2H), 3.53 (s, 3H), 3.31–3.24 (q, *J* = 7.7 Hz 1H), 3.07–3.00 (q, *J* = 7.7 Hz, 1H), 2.15–1.87 (m, 5H), 1.72–1.60 (m, 1H); ^13^C NMR (400 MHz, CDCl_3_): 199.98, 174.06, 138.02, 120.08, 128.78, 127.40, 55.36, 51.82, 47.77, 33.39, 30.08, 28.66, 24.15; IR(KBr): 2960.5, 2924.5, 2852.0, 1740.0, 1681.8, 1564.7, 1446.0 cm^–1^, HPLC analysis (Chiralpak OD-H column, λ = 254 nm, hexane/*i*-PrOH = 95/5, flow rate 1.0 mL/min): *t*R = 9.3 min (major), 11.7 min (minor).

### Methyl-(*R*)-5-(benzylthio)-3-methyl-5-oxopentanoate (**2e**)^12^

[α]_D_^25^ −0.158 (*c* 0.100, CHCl_3_); ^1^H NMR (300 MHz, CDCl_3_): 7.31–7.20 (m, 5H), 4.11 (s, 2H), 3.65 (s, 3H), 2.65–2.18 (m, 5H), 1.01 (d, *J* = 6.4 Hz, 3H), IR (KBr): 3479.1, 1702.9, 1415.6, 1247.8, 919.9, 713.6, 566.9 cm^–1^, HRMS(FAB+) for C_14_H_18_O_3_S: [M + H]^+^ calcd 267.3684; found, 267.1055; HPLC analysis (Chiralpak OD-H column, λ = 254 nm, hexane/*i*-PrOH = 95/5, flow rate 1.0 mL/min): *t*R = 8.7 min (major), 10.3 min (minor).

### (1*R*,6*S*)-6-((Cyclohexylthio)carbonyl)cyclohex-3-ene-1-carboxylic Acid (**2f**)

[α]_D_^25^ −0.115 (*c* 0.100, CHCl_3_); ^1^H NMR (300 MHz, CDCl_3_): 5.68 (s, 2H), 3.50 (s, 1H), 3.18–3.15 (m, 1H), 3.04–3.01 (m, 1H), 2.67–2.33 (m, 4H), 1.90–1.25 (m, 10H); ^13^C NMR (400 MHz, CDCl_3_): 201.69, 181.05, 137.72, 128.98, 128.84, 127.43, 53.07, 44.96, 33.26, 33.16, 30.39, 29.27, 25.44, 25.34 cm^–1^, HPLC analysis (Chiralpak OD-H column, λ = 254 nm, hexane/*i*-PrOH = 95/5, flow rate 1.0 mL/min): *t*R = 9.3 min (major), 11.7 min (minor).

### Methyl-(1*R*,6*S*)-6-((isopropylthio)carbonyl)cyclohex-3-ene-1-carboxylate (**2g**)^12^

[α]_D_^25^ −0.217 (*c* 0.100, CHCl_3_); ^1^H NMR (300 MHz, CDCl_3_): 5.68 (s, 2H), 3.68 (s, 1H), 3.17–3.11 (m, 1H), 3.05–3.00 (m, 1H), 2.63–2.31 (m, 5H), 1.30 (d, *J* = 6.8 Hz, 6H); ^13^C NMR (400 MHz, CDCl_3_): 125.75, 124.79, 58.14, 52.02, 48.33, 40.37, 34.74, 29.94, 26.55, 23.23, 23.16; IR (KBr): 3027.6, 2964.7, 2997.9, 2870.6, 2838.9, 1738.5, 1670.5, 1434.0, 1387.3, 1366.2, 1240.4, 1203.6, 1115.3 cm^–1^, HPLC analysis (Chiralpak OD-H column, λ = 254 nm, hexane/*i*-PrOH = 95/5, flow rate 1.0 mL/min): *t*R = 9.3 min (major), 11.7 min (minor).

### Methyl-(1*R*,6*S*)-6-((allylthio)carbonyl)cyclohex-3-ene-1-carboxylate (**2h**)

[α]_D_^25^ −0.109 (*c* 0.100, CHCl_3_); ^1^H NMR (300 MHz, CDCl_3_): 5.69 (s, 2H), 5.26 (d, *J* = 17.0 Hz, 1H), 5.11–5.07 (d, *J* = 9.9 Hz, 1H), 3.69 (s, 1H), 3.55–3.53 (d, *J* = 6.9 Hz, 2H), 3.22–3.17 (m, 1H), 3.08–3.03 (m, 1H), 2.66–2.33 (m, 5H); ^13^C NMR (400 MHz, CDCl_3_): 133.29, 125.84, 124.68, 118.10, 52.10, 48.22, 40.39, 31.82, 26.56, 26.14; IR (KBr): 3028.6, 2963.1, 2927.6, 2851.8, 1732.9, 1677.6, 1560.9 cm^–1^, HPLC analysis (Chiralpak OD-H column, λ = 254 nm, hexane/*i*-PrOH = 95/5, flow rate 1.0 mL/min): *t*R = 9.3 min (major), 11.7 min (minor).

### (1*R*,6*S*)-6-(Phenylcarbamoyl)cyclohex-3-ene-1-carboxylic Acid (**2i**)^13^

[α]_D_^25^ 0.045 (*c* 1.00, CH_3_Cl), mp 78–81 °C, ^1^H NMR (300 MHz, DMSO): δ 12.17 (s, 1H), 9.77 (s, 1H), 7.62 (d, 2H, *J* = 8.2 Hz), 7.31 (t, 2H, *J* = 7.3 Hz), 7.05 (t, 1H, *J* = 7.2 Hz), 5.7 (s, 2H), 3.08 (d, 1H, *J* = 3.6 Hz), 2.93 (d, 1H, *J* = 3.6 Hz), 2.65 (dd, 2H, *J* = 6,4 Hz), 2.32 (dd, 2H, *J* = 5.5, 4.4 Hz), ^13^C NMR (100 MHz, DMSO): δ 175.03, 172.10, 139.50, 128.55, 125.71, 124.54, 122.77, 119.08, 40.05, 39.21, 26.71, 26.04, IR (KBr): 3349.75, 3029.62, 2921.63, 1708.62, 1546.63, 1209.15, 944.95, 754.03, 566.97 cm^–1^, HRMS (FAB+) for C_14_H_16_NO_3_: [M + H]^+^ calcd 246.2868; found, 246.1130.

### (1*S*,2*R*,4*R*)-3-(Phenylcarbamoyl)bicyclo[2.2.2]oct-5-ene-2-carboxylic Acid (**2j**)^13^

[α]_D_^25^ 7.1 (*c* 1.00, CH_3_Cl), mp 143–148 °C, ^1^H NMR (300 MHz, DMSO): δ 11.65 (s, 1H), 9.77 (s, 1H), 7.51 (d, 2H, *J* = 8.5 Hz), 7.24 (t, 2H, *J* = 5.0 Hz), 6.98 (t, 1H, *J* = 4.5 Hz), 6.26 (t, 1H, *J* = 7.3 Hz), 6.10 (t, 1H, *J* = 7.3 Hz), 3.13 (d, 1H, *J* = 5.4 Hz), 2.86 (d, 1H, *J* = 5.4 Hz), 2.76 (s, 2H), 1.55 (d, 2H, *J* = 3.15 Hz), 1.25–1.16 (m, 2H), ^13^C NMR (100 MHz, DMSO): δ 170.99, 139.75, 133.05, 132.65, 131.31, 128.44, 122.51, 118.97, 113.84, 49.38, 44.60, 33.59, 31.64, 31.32, 24.62, 24.42, 22.48, IR (KBr): 3139.54, 2940.91, 1731.76, 1566.27, 1442.49, 1292.07, 1083.79, 981.59, 782.96, 757.89, 514.90 cm^–1^, HRMS (FAB+) for C_16_H_18_NO_3_: [M + H]^+^ calcd 272.3284; found, 272.1287.

### (1*S*,2*R*,3*S*,4*R*)-3-(Phenylcarbamoyl)bicyclo[2.2.1]hept-5-ene-2-carboxylic Acid (**2k**)^13^

[α]_D_^25^ 7.1 (*c* 1.00, CH_3_Cl), mp 110–113 °C, ^1^H NMR (300 MHz, DMSO): δ 11.67 (s, 1H), 9.86 (s, 1H), 7.52 (d, 2H, *J* = 7.9 Hz), 7.24 (t, 2H, *J* = 7.8 Hz), 6.98 (t, 1H, *J* = 7.1 Hz), 6.21 (dd, 1H, *J* = 2.7, 2.7 Hz), 6.03 (dd, 1H, *J* = 3.1, 3.1 Hz), 3.22 (d, 1H, *J* = 3.1 Hz), 3.11 (d, 1H, *J* = 3.1 Hz), 3.07 (s, 1H), 3.00 (s, 1H), 1.30 (dd, 2H, *J* = 7.8, 8.3 Hz), ^13^C NMR (100 MHz, DMSO): δ 173.46, 170.05, 139.62, 135.02, 133.84, 128.53, 128.53, 122.64, 118.95, 49.19, 48.78, 48.19, 46.85, 45.44, IR (KBr): 3382.53, 3145.33, 2829.12, 1712.48, 1546.63, 1446.35, 1176.38, 927.59, 754.03, 694.25, 511.04 cm^–1^, HRMS (FAB+) for C_15_H_16_NO_3_: [M + H]^+^ calcd 258.2978; found, 258.1130.

### (1*R*,3*S*)-3-(Phenylcarbamoyl)cyclohexane-1-carboxylic Acid (**2l**)^13^

[α]_D_^25^ −0.21 (*c* 1.00, CH_3_Cl), mp 188–192 °C, ^1^H NMR (300 MHz, DMSO): δ 12.25 (br s, 1H), 9.88 (s, 1H), 7.60 (d, 2H, *J* = 7.4 Hz), 7.28 (t, 2H, *J* = 7.8 Hz), 7.01 (t, 1H, *J* = 7.3 Hz), 2.40 (q, 1H), 2.27 (q, 1H), 2.02–1.81 (m, 5H), 1.50–1.23 (m, 6H), ^13^C NMR (100 MHz, DMSO): δ 173.62, 139.39, 128.60, 122.92, 119.05, 43.95, 41.86, 31.45, 28.53, 28.19, IR (KBr): 3299.77, 3079.91, 1700.99, 1544.78, 1309.49, 1251.64, 989.35, 755.99, 507.21 cm^–1^, HRMS (FAB+) for C_14_H_18_NO_3_: [M + H]^+^ calcd 248.3027; found, 248.1287.

### (*S*)-3-Methyl-5-oxo-5-(phenylamino)pentanoic Acid (**2m**)^13^

[α]_D_^25^ −0.20 (*c* 1.00, CH_3_Cl), mp 110–113 °C, ^1^H NMR (300 MHz, DMSO): δ 12.17 (br s, 1H), 9.77 (s, 1H), 7.62 (d, 2H, *J* = 8.2 Hz), 7.31 (t, 2H, *J* = 7.3 Hz), 7.05 (t, 1H, *J* = 7.2 Hz), 5.7 (s, 2H), 3.08 (d, 1H, *J* = 3.6 Hz), 2.93 (d, 1H, *J* = 3.6 Hz), 2.65 (dd, 2H, *J* = 6, 4.6 Hz), 2.32 (dd, 2H, *J* = 5.5, 4.4 Hz), ^13^C NMR (100 MHz, DMSO): δ 175.03, 172.10, 139.50, 128.55, 125.71, 124.54, 122.77, 119.08, 40.05, 39.21, 26.71, 26.04, IR (KBr): 3309.41, 1658.56, 1535.14, 1187.99, 910.28, 755.99, 593.99 cm^–1^, HRMS (FAB+) for C_12_H_16_NO_3_: [M + H]^+^ calcd 222.2647; found, 222.1130.

### (1*R*,2*S*)-2-(Phenylcarbamoyl)cyclohexane-1-carboxylic Acid (**2n**)^13^

[α]_D_^25^ 0.045 (*c* 1.00, CH_3_Cl), mp 188–192 °C, ^1^H NMR (300 MHz, DMSO): δ 11.93 (br s, 1H), 9.70 (s, 1H), 7.56 (d, 2H, *J* = 7.7 Hz), 7.25 (t, 2H, *J* = 7.7 Hz), 6.98 (t, 1H, *J* = 7.0 Hz), 2.93 (d, 1H, *J* = 4.7 Hz), 2.57 (p, 1H, *J* = 4.6 Hz), 2.13–1.98 (m, 2H), 1.75–1.62 (m, 2H), 1.39 (m, 4H), ^13^C NMR (100 MHz, DMSO): δ 175.03, 172.10, 139.50, 128.55, 125.71, 124.54, 122.77, 119.08, 40.05, 39.21, 26.71, 26.04, IR (KBr): 3309.41, 2923.70, 1720.28, 1550.56, 1442.56, 1303.71, 1025.99, 887.14, 755.99 cm^–1^, HRMS (FAB+) for C_14_H_18_NO_3_: [M + H]^+^ calcd 248.3027; found, 248.1287.

### (1*S*,2*S*,3*R*,4*R*)-3-(Phenylcarbamoyl)-7-oxabicyclo[2.2.1]hept-5-ene-2-carboxylic Acid (**2o**)^13^

[α]_D_^25^ 0.90 (*c* 1.00, CH_3_Cl), mp 147–151 °C, ^1^H NMR (300 MHz, DMSO): δ 12.17 (br s, 1H), 9.77 (s, 1H), 7.62 (d, 2H, *J* = 8.2 Hz), 7.31 (t, 2H, *J* = 7.3 Hz), 7.05 (t, 1H, *J* = 7.2 Hz), 5.7 (s, 2H), 3.08 (d, 1H, *J* = 3.6 Hz), 2.93 (d, 1H, *J* = 3.6 Hz), 2.65 (dd, 2H, *J* = 6, 4.6 Hz), 2.32 (dd, 2H, *J* = 5.5, 4.4 Hz), ^13^C NMR (100 MHz, DMSO): δ 175.03, 172.10, 139.50, 128.55, 125.71, 124.54, 122.77, 119.08, 40.05, 39.21, 26.71, 26.04. IR (KBr): 3324.84, 2931.41, 1704.84, 1535.14, 1319.14, 964.28, 755.99 cm^–1^, HRMS (FAB+) for C_14_H1_4_NO_4_: [M + H]^+^ calcd 260.2701; found, 260.0923.

### (2*R*,3*R*)-2,3-Dimethyl-4-oxo-4-(phenylamino)butanoic Acid (**2p**)

[α]_D_^25^ −0.17 (*c* 1.00, CH_3_Cl), mp 130–135 °C, ^1^H NMR (300 MHz, DMSO): δ 10.03 (s, 1H), 9.93 (s, 1H), 7.60 (d, 2H, *J* = 7.4 Hz), 7.30 (t, 2H, *J* = 7.9 Hz), 7.04 (t, 1H, *J* = 8.4 Hz), 2.70–2.60 (m, 1H), 1.09 (t, 6H, *J* = 7.0 Hz), ^13^C NMR (100 MHz, DMSO): δ 173.64, 170.21, 139.24, 128.99, 123.08, 119.14, 43.06, 40.65, 27.39, 19.49, IR (KBr): 3286.27, 2923.70, 1704.85, 1535.14, 1434.85, 1303.71, 1164.85, 941.14, 740.57 cm^–1^, HRMS(FAB+) for C_12_H_16_NO_3_: [M + H]^+^ calcd 222.2647; found, 222.1130.

### Synthesis of (3*aR*,4*S*,7*R*,7*aS*)-2-Hydroxy-3*a*,4,7,7*a*-tetrahydro-1*H*-4,7-methanoisoindole-1,3(2*H*)-dione (*N*-Hydroxy-5-endo-norbornene-2,3-dicarboximide) (**3b**)

The cyclic meso-anhydride (0.33 mmol) and catalyst (5 mol %) were added to a reaction vessel at room temperature and then dissolved in toluene (0.2 M). The reaction vessel was placed in a thermostat set to 0 °C and stirred for 10 min before the addition of BnSH (1.2 equiv). After 24 h, the solvent was removed under reduced pressure, and column chromatography (SiO2, CH_2_Cl_2_/methanol = 20:1) afforded the product. Suspension of the appropriate crude product and excess ammonium acetate (10 g) in glacial acetic acid (25 mL) was refluxed with stirring for 10 h. The reaction mixture was cooled and poured into ice-cold water (100 mL); the precipitated yellow solid was filtered and recrystallized from chloroform to give the pure imide. To a suspension of the imide (10 mmol) in 5 mL of acetonitrile at room temperature was added di*tert*-butyl dicarbonate (20 mmol), followed by DMAP (10 mol %). Hydroxylamine aqueous solution (50 wt % aqueous solution, 10 mmol) was added. After the mixture was stirred at room temperature for 12 h, 10 mL of ether was added to precipitate most of the hydroxylammonium salt of *N*-hydroxyimide. The solid was filtered off, washed thoroughly with ether, and dried. Then, it was dispersed in 15 mL of water, and diluted HCl was added until pH 1 was reached. The aqueous phase was saturated with NaCl and extracted several times with ethyl acetate. The combined organic extracts were dried over Na_2_SO_4_, and the solvent was removed under reduced pressure. The precipitated solid was recrystallized from ethyl acetate to give the pure solids.

### (3*aR*,4*S*,7*R*,7*aS*)-2-Hydroxy-3*a*,4,7,7*a*-tetrahydro-1*H*-4,7-methanoisoindole-1,3(2*H*)-dione (*N*-Hydroxy-5-endo-norbornene-2,3-dicarboximide) (**3a**)^14^

^1^H NMR (300 MHz, DMSO): δ 10.52 (s, −OH), 6.10–6.02 (m, 2H, H-5, H-6), 3.28–3.26 (m, 2H, H-2, H-3), 3.24–3.21 (m, 2H, H-1, H-4), 1.57 (d, *J* = 8.65 Hz, 1H, H-7exo), 1.49 (d, *J* = 8.65 Hz, 1H, H-7endo); ^13^C NMR (100 MHz, DMSO): δ 173.37 (C=O, 2 C), 134.83 (CH, C-5, C-6), 51.45 (CH2, C-7), 44.30 (CH, C-1, C-4), 42.55 (CH, C-2, C-3); LRMS(FAB+) for C_9_H_10_NO_3_: [M + H]^+^ calcd 180; found, 180.

### Synthesis of *O*-(5-Endo-norbornene-2,3-dicarboximido)-*N*,*N*,*N*,*N*-tetramethyluronium Tetrafluoroborate (**3b**)

To a solution of 1,1,3,3-tetramethylurea (20 mmol) and DMF (0.3 mL) in CH_2_Cl_2_ (20 mL) was added oxalyl chloride (24 mmol), dropwise, at room temperature. The solution was refluxed for 3 h. The solvent was evaporated, and the resulting solid was stirred with some CH_2_Cl_2_ (2 × 10 mL), and the organics were evaporated after each treatment. The obtained crude chlorouronium salt was dissolved in MeCN (15 mL), and KBF4 (24 mmol) was added. The mixture was stirred at room temperature for 1 h, and to the resulting suspension was added **3a** (20 mmol). Triethylamine (24 mmol) was added dropwise while maintaining the temperature below 25 °C. The resulting suspension was stirred at 85 °C for 16 h. The solution was filtered through a plug of celite, and the solvent was evaporated (15 Torr) and crystallized from MeOH/2-propanol to give the uronium salts **3b**.

### *O*-(5-Endo-norbornene-2,3-dicarboximido)-*N*,*N*,*N*,*N*-tetramethyluronium Tetrafluoroborate (**3b**)

^1^H NMR (300 MHz, DMSO): δ 6.29 (m, 2H, H-5, H-6), 3.60–3.59 (m, 2H, H-2, H-3), 3.37 (m, 2H, H-1, H-4), 3.09 (s, 12H, H-8), 1.65 (d, *J* = 8.65 Hz, 1H, H-7exo), 1.60 (d, *J* = 8.65 Hz, 1H, H-7endo), ^13^C NMR (100 MHz, DMSO): δ 173.37 (C=O, 2 C),134.83 (CH, C-5, C-6), 51.45 (CH2, C-7), 44.30 (CH, C-1, C-4), 42.55 (CH, C-2, C-3); LRMS(FAB+) for C_14_H_20_N_3_O_3_^+^ [M]^+^: calcd 278; found, 278.

## Supporting Information Available

Characterization of products (copies of ^1^H, ^13^C, and HPLC, GC spectra) (PDF)
