# Supplementary material for: Enantioselective Thiolysis and Aminolysis of Cyclic Anhydrides Using a Chiral Diamine-Derived Thiourea Catalyst
Source: ACS Omega. 2021 Nov 24;6(50):34501–11. doi: 10.1021/acsomega.1c04741 (PMC8697410; doi:10.1021/acsomega.1c04741)
Supplement: Supplementary file 1 — ao1c04741_si_001.pdf [file ao1c04741_si_001.pdf]

# Supporting Information

# Enantioselective Thiolysis and Aminolysis of Cyclic Anhydrides Using a Chiral-Diamine-Derived Thiourea Catalyst

*Jae Ho Shim,* Sung Joo Park, Byung Kook Ahn, Ji Yeon Lee, Hyeon Soo Kim and*

*Deok-Chan Ha**

*Department of Anatomy, Korea University College of Medicine, 46, Gaeunsa 2-gil, Seongbuk-gu, Seoul, 02842, Republic of Korea*

*E-mail: shimjh3000@korea.ac.kr*

*Department of Chemistry, Korea University , 145 Anam-ro Seongbuk-gu , Seoul 02841, Korea*

*E-mail: dechha@korea.ac.kr*

## Table of Contents

**1. Instruments and reagents…………………………………………………………… S-2**

**2 Compound Characterization Data…………………………………………………… S-2**

**3. Copy of NMR and MASS Spectra…………………………………………………... S-9**

**4. Copy of HPLC Chromatograms………………………………………………………S-47**

**5. Copy of GC Chromatograms…………………………………………………………S-56**

**6. Computational Results of DFT Calculations for all Calculated Structures………. S-70**

# 1. Instruments and reagents

IR spectrum was recorded using Bomen MB-104 spectrophotometer, and optical rotation was measured using Rudolph Research Autopol III polarimeter. 1H NMR and 13C NMR spectrum were obtained using Varian Germini 300 (300, 75 MHz) and Bruker AMX 400 (100, 75 MHz) using DMSO and TMS or $CDCl_3$ internal standards. HRMS spectra were obtained using a JEOL JMS-AX505WA mass spectrometer. For GC analysis, Hewlett Packard 5890 series Ⅱ plus Gas Chromatograph was used. All reactions were carried out under an argon environment in well-dried flasks in an oven. Toluene ($CaH_2$), THF (Na, benzophenone), and $CH_2Cl_2$ ($CaH_2$) reaction solvents were purified before use. The reagents used in this study were products such as Aldrich, Acros, Sigma, Merck, Fluka, TCI, Lancaster, and Kankyo chemical, and if necessary, purified or dried by a known method. Merck's silica gel 60 (230-400mesh) was used as a stationary phase for column chromatography.

# 2. Compound Characterization Data

### 1-(2-Amino-1,2-diphenylethyl)-3-phenylthiourea (1a)

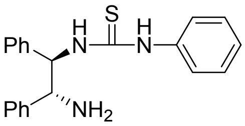


$[\alpha]_D^{25}$ = 0.153 (c = 1.00, $CH_3Cl$); m.p. 130-132 ℃; $^1$H NMR (300 MHz, $CDCl_3$) δ 7.76 (s, 1H), 7.54-7.19 (m, 15H), 5.54 (s, 1H), 4.42 (d, 1H, $J$ = 5 Hz), 1.35 (br s, 1H); $^{13}$C NMR (100 MHz, DMSO) δ 182.09, 134.48, 133.93, 129.89, 128.70, 128.10, 127.91, 127.15, 126.94, 126.82, 126.74, 126.23, 125.59, 125.24, 122.98, 63.07, 59.09; FTIR (KBr) 3287.86, 3027.84, 1521.63, 1241.99, 1072.28,939.20, 698.13 $cm^{-1}$; HRMS (FAB+) for $C_{21}H_{22}N_3S$ $[M+H]^+$ Calcd: 348.4918, Found: 348.1534.

### 1-[2-(1-Ethylpropylamino)-1,2-diphenylethyl]-3-phenylthiourea (1b)

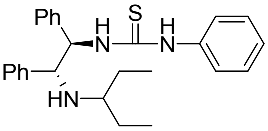


$[\alpha]_D^{24}$ = ＋73.9 ($c$ = 0.105, $CHCl_3$); $^1$H NMR (300 MHz, DMSO-$d_6$) δ 9.86 (s, 1H), 8.03 (d, $J$ = 7.4 Hz, 1H), 7.46 (d, $J$ = 8.0 Hz, 2H), 7.36 (t, $J$ = 7.4 Hz, 3H), 7.30~7.13 (m, 10H), 5.5 (t, $J$ = 5.8 Hz, 1H), 4.09 (d, $J$ = 5.2 Hz, 1H), 2.03 (br s, 1H), 0.94 (br s, 1H), 1.21~1.10 (m, 4H), 0.60 (t, $J$ = 7.4 Hz, 3H), 0.44 (t, $J$ = 7.4 Hz, 3H); $^{13}$C NMR (400 MHZ, DMSO-$d_6$) δ 180.31, 141.23, 140.78, 128.75, 127.96, 127.85, 127.81, 126.87, 126.72, 124.54, 123.31, 63.69, 63.06, 55.34, 26.02, 23.31, 10.30, 7.73; IR (KBr) 3164.6, 2960.2, 2345.0, 1949.7, 1735.6, 1508.1, 1378.9, 1240.0, 1027.9, 912.2, 507.1 $cm^{-1}$; HRMS(FAB+) for $C_{26}H_{31}N_3S[M+H]^+$ Calcd: 418.2239, Found: 418.2317.

### 1-[2-(1-Methylethylamino)-1,2-diphenylethyl]-3-(4-nitrophenyl)thiourea (1c)

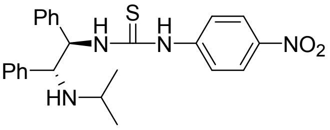

$[\alpha]_D^{24}$ = +48.1 (*c* = 0.105, $CHCl_3$); $^1$H NMR (300 MHz, DMSO-$d_6$) δ 10.5 (br s, 1H), 8.15 (dd, *J* = 14.0, 9.0 Hz, 2H), 7.93 (d, *J* = 8.8 Hz, 2H), 7.78 (d, *J* = 8.5 Hz, 1H), 7.28~7.12 (m, 10H), 5.43 (d, *J* = 6.3 Hz, 1H), 4.14 (d, *J* = 6.9 Hz, 1H), 2.45~2.37 (m, 1H), 0.94 (d, *J* = 6.3 Hz, 3H), 0.87 (d, *J* = 6.3 Hz, 3H); $^{13}$C NMR (400 MHZ, DMSO-$d_6$) δ 179.57, 146.41, 127.89, 127.39, 126.91, 126.70, 124.52, 120.01, 63.83, 63.34, 45.09, 24.00, 21.51; IR(KBr) 3322.9, 2960.3, 1594.9, 1519.7, 1342.3, 1110.9, 852.4, 698.1, 513.0 $cm^{-1}$; HRMS(FAB+) for $C_{24}H_{27}O_2N_4S[M+H]^+$ Calcd : 435.1855, Found : 435.1848.

### (*R*,*R*)-1-[2-(1-Ethylpropylamino)-1,2-diphenylethyl]-3-(4-fluorophenyl) thiourea (1d)

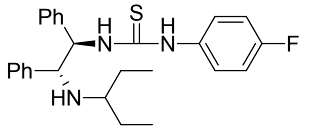

$[\alpha]_D^{25}$ = +17.88 (c = 0.02, $CHCl_3$); $^1$H NMR (300 MHz, $CD_3OD$) 9.86 (s, 1 H), 8.01 (d, *J* = 6.7 Hz, 1 H), 7.46~7.18 (m, 14 H), 5.47 (s, 1 H), 4.10 (d, *J* = 5.5 Hz, 1 H), 2.04 (t, *J* = 5.4 Hz, 2 H), 1.47 (s, 1 H), 1.23~1.10 (m, 4 H), 0.70 (t, *J* = 10.1, 3 H), 0.44 (t, *J* = 7.0 Hz, 3 H); $^{13}$C NMR (400 MHz, $CD_3OD$) 180.72, 160.34, 157.94, 141.24, 140.75, 135.29, 127.97, 127.87, 127.83, 126.83, 126.96, 126.89, 126.75, 125.76 ,63.71, 63.08, 26.04, 23.36, 10.30, 7.74; IR (KBr) 3193.7, 2962.3, 1889.9, 1511.9, 1218.8, 848.6, 701.9, 555.42 $cm^{-1}$; HRMS (FAB+) for $C_{26}H_{31}FN_3S$ $[M+H]^+$ Calcd : 436.6172, Found : 436.2223. pattern 436.5, 349.3, 266.4, 176.3, 106.1

### (*R*,*R*)-*N*-[2-(1-Ethylpropylamino)-1,2-diphenylethyl]-*N′*-(4-methoxyphenyl) thiourea (1e)

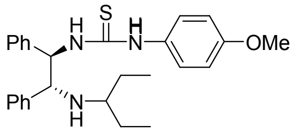

$[\alpha]_D^{25}$ = +307.8 (c 0.100, $CHCl_3$); $^1$H NMR (300 MHz, DMSO-$d_6$) 9.64 (s, 1H), 7.73 (s, 1H), 7.43~7.21 (m, 13H), 6.98 (d, *J* = 6.7 Hz, 2H), 5.41 (s, 1H), 4.05 (d, *J* = 5.7 Hz, 1H), 3.75 (s, 3H), 1.99 (m, 1H), 1.12 (m, 4H), 0.65 (m, 3H), 0.39 (m, 3H); IR(KBr) 3168.6, 2958.4, 1731.8, 1510.1, 1297.9, 1243.9, 1029.9, 831.2, 700.1, 570.9 $cm^{-1}$; HRMS(FAB+) for $C_{27}H_{34}N_3SO$ [M+H]+ Calcd : 448.6530, Found : 448.2423

### (*R*,*R*)-1-[2-(1-Ethylpropylamino)-1,2-diphenylethyl]-3-(4-methylphenyl)thiourea (1f)

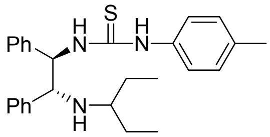

$[\alpha]_D^{24}$ = +42.9 (*c* = 0.02, $CHCl_3$); $^1$H NMR (300 MHZ, $CD_3OD$) δ 9.74 (br s, 1 H), 7.88 (d, *J* = 7.1 Hz, 1 H),

7.31~7.18 (m, 13 H), 5.43 (m, 1 H), 4.07 (d, *J* = 3.6 Hz, 1 H), 2.91 (s, 3 H), 2.00 (br s, 1 H), 1.39 (br s, 1 H), 1.15~1.09 (m, 4 H), 0.67 (t, *J* = 7.1 Hz, 3 H), 0.41 (t, *J* = 7.1 Hz, 3 H); $^{13}$C NMR (400 MHZ, $CD_3OD$) δ 180.34, 141.18, 140.89, 136.05, 134.19, 129.35, 127.99, 127.86, 126.97, 126.82, 126.72, 123.98, 63.60, 63.10, 55.17, 26.03, 23.37, 20.52, 10.26, 7.64; IR (KBr) 3180.2, 2958.4, 1948.8, 1510.1, 1240.1, 821.6, 700.1, 565.1 cm$^{-1}$; HRMS (FAB+) for $C_{27}H_{34}N_3S$ $[M+H]^+$ Calcd: 432.2473, Found: 432.6537, pattern 432.5, 345.3, 266.4, 176.3, 106.01

**(*R*,*R*)-1-[2-(1-Ethylpropylamino)-1,2-diphenylethyl]-3-[4-(trifluorometh y)phenyl]thiourea (1g)**

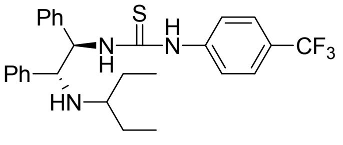


$[\alpha]_D^{24}$ = +45.5 (*c* = 0.02, $CHCl_3$); $^1$H NMR (300 MHz, DMSO-$d_6$) δ 10.2 (br s, 1H), 8.41 (br s, 1H), 7.79 (d, *J* = 8.0 Hz, 2H), 7.64 (d, *J* = 8.5 Hz, 2H), 7.35~7.15 (m, 10H), 5.53 (br s, 1H), 4.13 (d, *J* = 5.5 Hz, 1H), 2.07 (m, 1H), 1.30~1.15 (m, 4H), 0.73 (t, *J* = 7.1 Hz, 3H), 0.49 (t, *J* = 6.9 Hz, 3H); $^{13}$C NMR (400 MHZ, $CDCl_3$) δ 140.10, 128.98, 128.69, 128.50, 128.01, 127.91, 127.72, 127.28, 126.70, 125.05, 65.12, 64.34, 55.50, 26.65, 24.22, 10.53, 7.95; IR(KBr) 3205.3, 2962.3, 1945.9, 1741.5, 1517.8, 1324.9, 1245.9, 1066.5, 840.9, 700.1, 597.9 cm$^{-1}$; HRMS(FAB+) for $C_{27}H_{31}F_3N_3S[M+H]^+$ Calcd: 486.2191, Found: 486.2190.

**(*R*,*R*)-1-[2-(1-Ethylpropylamino)-1,2-diphenylethyl]-3-pentafluorophenylthiourea (1h)**

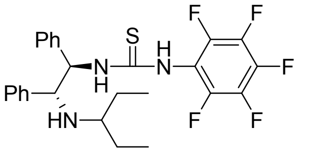


$[\alpha]_D^{24}$ = +80.4 (*c* = 0.02, $CHCl_3$); $^1$H NMR (300 MHz, $CD_3OD$) δ 9.47 (s, 1 H), 8.61 (s, 1 H), 7.30~7.15 (m, 10 H), 5.48 (br s, 1 H), 4.13 (d, *J* = 6.1 Hz, 1 H), 2.08 (m, 1 H), 1.54 (br, 1 H), 1.30~1.14 (m, 4 H), 0.73 (t, *J* = 7.4 Hz, 3 H), 0.55 (t, *J* = 6.3 Hz, 3 H); $^{13}$C NMR (400 MHZ, $CD_3OD$) δ 182.94, 145.63, 142.35, 141.21, 141.07, 140.06, 138.76, 137.76, 135.44, 128.04, 127.77, 126.94, 115.26, 65.04, 64.15, 26.10, 23.59, 10.62, 9.71; IR (KBr) 3299.8, 2964.2, 1525.5, 1344.2, 1145.6, 991.3, 912.2, 700.1, 605.6 cm$^{-1}$; HRMS (FAB+) for $C_{26}H_{27}F_5N_3S$ $[M+H]^+$ Calcd: 508.1846, Found: 508.1848.

**1-[2-(1-Propylbutylamino)-1,2-diphenylethyl]-3-(4-nitrophenyl)thiourea (1i)**

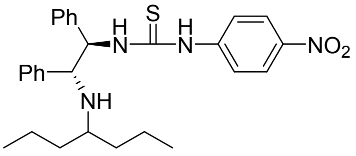


$[\alpha]_D^{24}$ = + 40.7 (*c* = 0.105, $CHCl_3$); $^1$H NMR (300 MHz, DMSO-$d_6$) δ 10.5 (br s, 1H), 8.17 (d, *J* = 9.1 Hz, 2H), 7.91 (d, *J* = 8.8 Hz, 2H), 7.36~7.17 (m, 10H), 5.53 (d, *J* = 4.4 Hz, 1H), 4.15 (d, *J* = 5.5 Hz, 1H), 2.16 (m, 1H), 1.32~1.12 (m, 8H), 1.03~0.82 (m, 4H), 0.70 (t, *J* = 6.9 Hz, 6H); IR(KBr) □ 3317.0, 2960.2, 2360.4, 2225.5,

1949.7, 1739.5, 1508.1, 1315.2, 1176.4, 1072.2, 837.0, 700.0, 545.8 $cm^{-1}$; HRMS(FAB+) for $C_{28}H_{35}O_2N_4S$[M+H]$^+$ Calcd : 491.2481, Found : 491.2501.

**(*R*,*R*)-1-[2-(1-Ethylpropylamino)-1,2-diphenylethyl]-3-[3,5-bis(trifluoromethyl)phenyl]-thiourea (1j)**

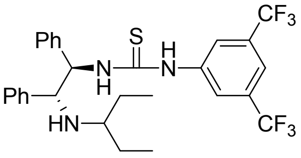


$[\alpha]_D^{25}$ = +49.9 (c = 0.02, $CHCl_3$); $^1$H NMR (300 MHz, $CD_3OD$) 10.5 (br, 1 H), 8.30 (s, 2 H), 7.74 (s, 1 H), 7.40~7.19 (m, 10 H), 5.57 (br, 1 H), 4.18 (d, *J* = 4.9 Hz, 1 H), 2.09 (m, 1 H), 1.24~1.20 (m, 4 H), 0.75 (t, *J* = 7.1 Hz, 3 H), 0.50 (t, *J* = 6.0 Hz, 3 H); $^{13}$C NMR (300 MHZ, $CDCl_3$) δ 140.06, 129.18, 128.89, 128.30, 128.16, 127.57, 126.89, 124.76, 119.71, 65.46, 56.57, 26.36, 24.46, 10.33, 8.44; IR (KBr) 3239.9, 2964.2, 1471.5, 1278.6, 1135.9, 885.2, 700.1 $cm^{-1}$; HRMS (FAB+) for $C_{28}H_{30}F_6N_3S$ [M+H]$^+$ Calcd: 554.2065, Found : 554.2065

**(*R*,*R*)-1-[2-(1-Ethylpropylamino)-1,2-diphenylethyl]-3-(4-nitrophenyl)thiourea (1k)**

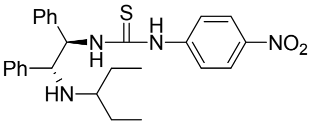


$[\alpha]_D^{24}$ = +37.7 (*c* = 0.02, $CHCl_3$); $^1$H NMR (300 MHz, $CD_3OD$) δ 10.5 (s, 1 H), 8.16 (m, 2 H), 7.90 (d, *J* = 9.1 Hz, 2 H), 7.37~7.15 (m, 10 H), 5.54 (br s, 1 H), 4.16 (d, *J* = 5.5 Hz, 1 H), 2.07 (m, 1 H), 1.30~1.15 (m, 4 H), 0.75 (t, *J* = 7.4 Hz, 3 H), 0.50 (t, *J* = 7.4 Hz, 3 H); $^{13}$C NMR (400 MHZ, $CD_3OD$) δ 179.85, 146.27, 141.82, 141.25, 140.25, 128.00, 127.86, 127.04, 126.98, 126.87, 124.60, 124.46, 120.28, 63.71, 63.14, 55.72, 26.04, 23.36, 10.32, 7.97; IR (KBr) 3330.5, 2960.2, 2599.6, 2456.4, 2345.0, 1951.6, 1743.3, 1496.5, 1346.1, 1110.8, 1072.2, 852.4, 700.0, 586.3 $cm^{-1}$; HRMS (FAB+) for $C_{26}H_{31}N_4O_2S$ [M+H]$^+$ Calcd: 463.2168, Found: 463.2165.

**(*R*,*R*)-1-[2-(1-Ethylpropylamino)-1,2-diphenylethyl]-3-(4-cyanophenyl)thiourea (1l)**

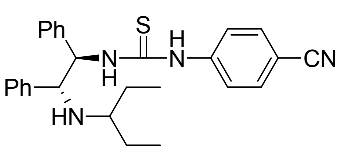


$[\alpha]_D^{24}$ = +55.5 (*c* = 0.02, $CHCl_3$); $^1$H NMR (300 MHz, DMSO-$d_6$) δ 10.3 (br s, 1 H), 8.54 (br s, 1 H), 7.84~7.72 (m, 4 H), 7.35~7.17 (m, 10 H), 5.54 (br s, 1 H), 4.14 (d, *J* = 5.2 Hz, 1 H), 2.07 (br s, 1 H), 1.56 (br s, 1 H), 1.21 (m, 4H), 0.74 (t, *J* = 7.4 Hz, 3 H), 0.49 (t, *J* = 6.9 Hz, 3H); $^{13}$C NMR (400 MHZ, DMSO-$d_6$) δ 180.16, 144.36, 141.39, 140.55, 132.98, 128.10, 127.24, 127.07, 121.34, 104.72, 63.97, 63.28, 55.91, 26.27, 23.56, 10.57, 8.16; IR (KBr) 3317.0, 2960.2, 2360.4, 2225.5, 1949.7, 1739.5, 1508.1, 1315.2, 1176.4, 1072.2, 837.0, 700.0, 545.8 $cm^{-1}$; HRMS (FAB+) for $C_{27}H_{31}N_4S$ [M+H]$^+$ Calcd: 443.2269, Found: 443.2271.

$C_{27}H_{34}N_3SO$ [M+H]$^+$ Calcd: 448.6530, Found : 448.2423

**1-[(1*R*,2*R*)-2-Amino-1,2-diphenylethyl]-3-[3,5-Bis(trifluoromethyl)phenyl]thiourea (1m)**

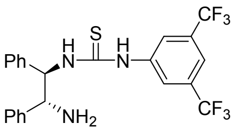

$[\alpha]_D^{25}$ = +13.5 (*c* 1.00, $CH_3Cl$); $^1$H NMR (300 MHz, DMSO-$d_6$) δ 10.70 (s, 1H), 8.32 (s, 2H), 7.71(s, 1H), 7.22~7.43(m, 13H), 5.57(d, *J* = 3 Hz, 1H), 4.44 (d, *J* = 3 Hz, 1H) ppm; $^{13}$C NMR (100 MHz, DMSO-$d_6$) δ 180.80, 143.41, 142.67, 130.94, 130.62, 128.81, 128.61 127.75, 127.57, 127.51, 125.25, 122.54, 121.68, 116.40, 63.86, 60.06 ppm; IR (KBr) 3305, 3032, 2963, 1652, 1601, 1557, 1383, 1277, 1262, 803, 700 $cm^{-1}$; HRMS ($FAB^+$) for $C_{22}H_{20}N_4S$ $[M+H]^+$ Calcd: 372.1487, Found: 372.1456

## 1-(2-Amino-1,2-diphenylethyl)-3-p-tolylthiourea (1n)

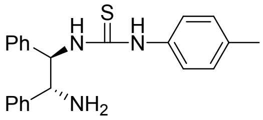

$[\alpha]_D^{25}$ = +0.087 (c = 1.00, $CH_3Cl$), m.p. 154-157 ℃; $^1$H NMR (300 MHz, $CDCl_3$) δ 7.58-7.07 (m, 15H), 5.53 (s, 1H), 4.42 (d, 1H, *J* = 2.7 Hz), 2.43 (s, 3H), 1.32 (br s, 3H); $^{13}$C NMR (100MHz, DMSO) δ 180.15, 143.12, 136.39, 133.64, 129.17, 128.11, 127.88, 126.98, 126.75, 123.28, 63.19, 59.28, 20.51; IR (KBr) 3301.69, 2861.98, 1889.99, 1527.42, 1342.28, 964.28, 701.99, 524.56 $cm^{-1}$; HRMS (FAB+) for $C_{22}H_{24}N_3S$ $[M+H]^+$ Calcd: 362.5188, Found: 362.1691.

## 1-(2-Amino-1,2-diphenylethyl)-3-naphthalen-1-ylthiourea (1o)

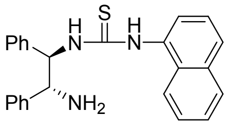

$[\alpha]_D^{25}$ = +0.157 (c = 1.00, $CH_3Cl$); m.p. 140-145℃; $^1$H NMR (300 MHz, $CDCl_3$) δ 7.99-6.94 (m, 18H), 5.49 (s, 1H), 4.27 (d, 1H, *J* = 2.7 Hz), 1.07 (br s, 1H); $^{13}$C NMR (100 MHz, DMSO) δ 181.93, 142.91, 141.46, 134.33, 133.97, 129.95, 128.16, 127.86, 126.99, 126.91, 126.76, 126.33, 125.66, 125.34, 122.95, 63.35, 59.18; FTIR (KBr) 3340.26, 3116.55, 1951.70, 1511.99, 1249.70, 941.13, 701.99, 632.56 $cm^{-1}$; HRMS (FAB+) for $C_{25}H_{24}N_3S$ $[M+H]^+$ Calcd: 398.5519,Found: 398.1691.

## 1-(2-Amino-1,2-diphenylethyl)-3-(2-fluorophenyl)thiourea (1p)

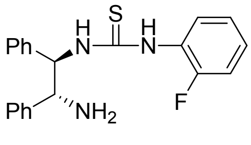

$[\alpha]_D^{25}$ = +0.189 (c = 1.00, $CH_3Cl$); m.p. 78-81℃; $^1$H NMR (300 MHz, $CDCl_3$) δ 7.51-7.12 (m, 14H), 5.43 (s, 1H), 4.40 (d, 1H, *J* = 3.3 Hz), 1.44 (br s, 3H); $^{13}$C NMR (100 MHz, DMSO) δ 180.56, 159.99, 157.61, 143.09, 141.42, 135.61, 128.09, 127.86, 127.00, 126.87, 126.79, 126.68, 125.08, 63.17, 59.34; FTIR (KBr) 3299.77, 3027.84, 1523.56, 1261.28, 1029.85, 937.28, 700.07 $cm^1$; HRMS (FAB+) for $C_{21}H_{21}FN_3S$ $[M+H]^+$ Calcd: 366.4822, Found: 366.1440.

## 1-(2-Amino-1,2-diphenylethyl)-3-(4-nitrophenyl)thiourea (1q)

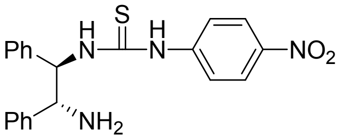

$[\alpha]_D^{25}$ = +0.157 (c = 1.00, $CH_3Cl$); m.p. 80-83 ℃; $^1$H NMR (300 MHz, $CDCl_3$) δ 7.63 (s, 1H), 7.40-7.17 (m, 14H), 5.50 (s, 1H), 4.42 (d, 1H, *J* = 3 Hz), 1.44 (br s, 3H); $^{13}$C NMR (100 MHz, DMSO) δ 160.01, 157.54, 143.07, 141.22, 128.07, 127.87, 127.09, 126.90, 126.73, 111.98, 111.76, 63.94, 59.41; FTIR (KBr) 3292.71,2933.52, 1951.71, 1589.14,1334.56, 848.57, 701.99 cm$^{-1}$; HRMS (FAB+) for $C_{21}H_{21}F_2N_4O_2S$ $[M+H]^+$ Calcd: 393.4892, Found: 393.1385

## 1-(2-Amino-1,2-diphenylethyl)-3-(4-methoxyphenyl)thiourea (1r)

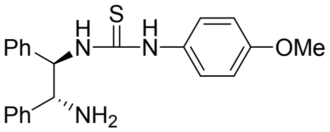

$[\alpha]_D^{25}$ = +0.327 (c = 1.00, $CH_3Cl$); m.p. 100-103℃; $^1$H NMR (300 MHz, $CDCl_3$) δ 7.38-6.96(m, 14H), 5.53 (s, 1H), 4.38 (d, 1H, *J* = 3.0 Hz), 1.30(br s, 3H); $^{13}$C NMR (100 MHz, DMSO) δ 181.93, 142.91, 141.46, 134.33, 133.97, 129.95, 128.16, 127.86, 126.99, 126.91, 126.76, 126.33, 125.66, 125.34, 122.95, 63.35, 59.18; FTIR (KBr) 3303.63, 3027.84, 1733.78, 1510.06, 1297.92, 1243.92, 1029.85, 831.21, 700.07, 568.93 cm$^{-1}$; HRMS (FAB+) for $C_{22}H_{24}N_3OS$ $[M+H]^+$ Calcd: 378.5181, Found: 378.1640.

13C nmr

## 1-(2-Amino-1,2-diphenylethyl)-3-(2,6-dimethylphenyl)thiourea (1s)

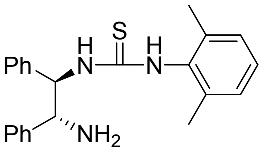

$[\alpha]_D^{25}$= +0.200 (c 1.00, $CH_3Cl$); m.p. 158-160 ℃; $^1$H NMR (300 MHz, $CDCl_3$) δ 7.39-6.81, 14H), 5.42 (d, 1H, *J* = 5.5 Hz), 4.32 (d, 1H, 2.8Hz), 2.40(s, 3H), 1.86 (s, 1H), 1.25 (br s, 1H); $^{13}$C NMR (100 MHz, DMSO) δ 179.82, 141.73, 137.33, 136.48, 128.59, 128.16, 126.72, 126.49, 63.31, 58.45, 17.95, 17.93; IR (KB, cm$^{-1}$) 3299.77, 3029.77, 1517.78, 1230.42, 1029.85, 912.01, 903.29, 701.99, 548.99 cm$^{-1}$; HRMS (FAB+) for $C_{23}H_{26}N_3S$ $[M+H]^+$ Calcd: 376.5458, Found: 376.1847.

## 1-(2-Amino-1,2-diphenylethyl)-3-(4-fluorophenyl)thiourea (1t)

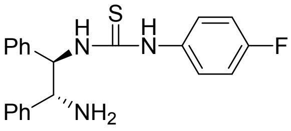

$[\alpha]_D^{25}$= + 0.132 (c = 1.00, $CH_3Cl$), m.p. = 78-81 ℃, $^1$H NMR (300 MHz, $CDCl_3$) δ 7.63 (s, 1H), 7.40-7.17 (m,

14H), 5.50 (s, 1H), 4.42 (d, 1H, $J$ = 3 Hz), 1.44 (br s, 3H), $^{13}$C NMR (100 MHz, DMSO) δ 180.56, 143.10, 141.42, 135.61, 128.09, 127.87, 127.01, 126.87, 126.69, 125.10, 115.25, 115.03, IR (KBr) 3301.69, 3029.77, 1874.56, 1527.42, 1342.27, 1218.85, 840.85, 701.99 cm$^{-1}$; HRMS (FAB+) for $C_{21}H_{21}FN_3S$ $[M+H]^+$, Calcd: 366.4822, Found: 366.1440.

## 3. Copy of NMR and MASS Spectra

### 1-(2-Amino-1,2-diphenylethyl)-3-phenylthiourea (1a)

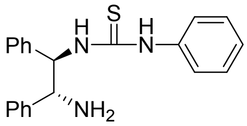


**Figure S1.** [1]**H nmr**

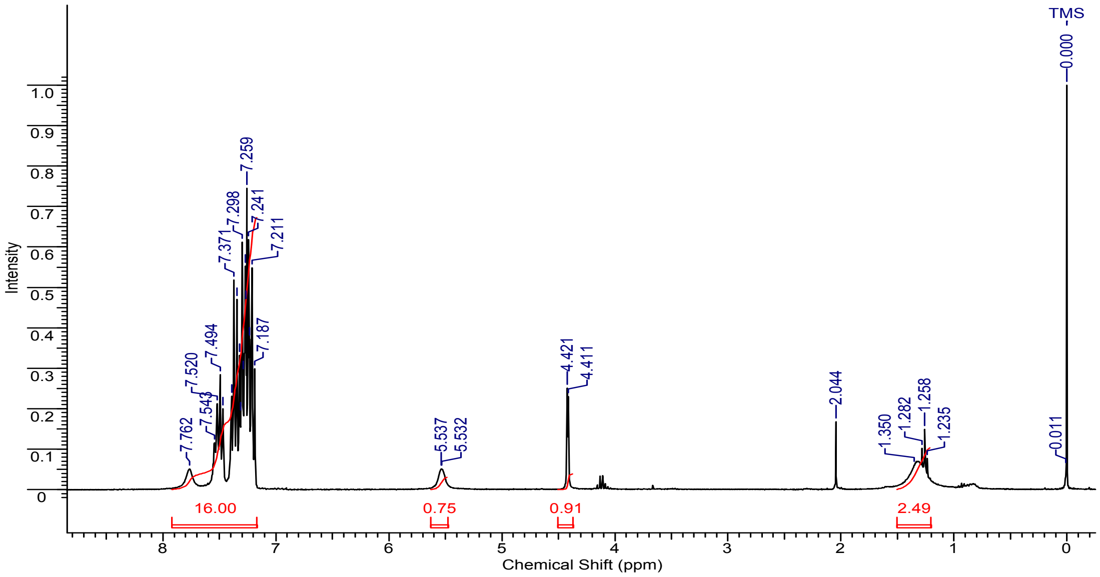


**Figure S2.** [13]**C nmr**

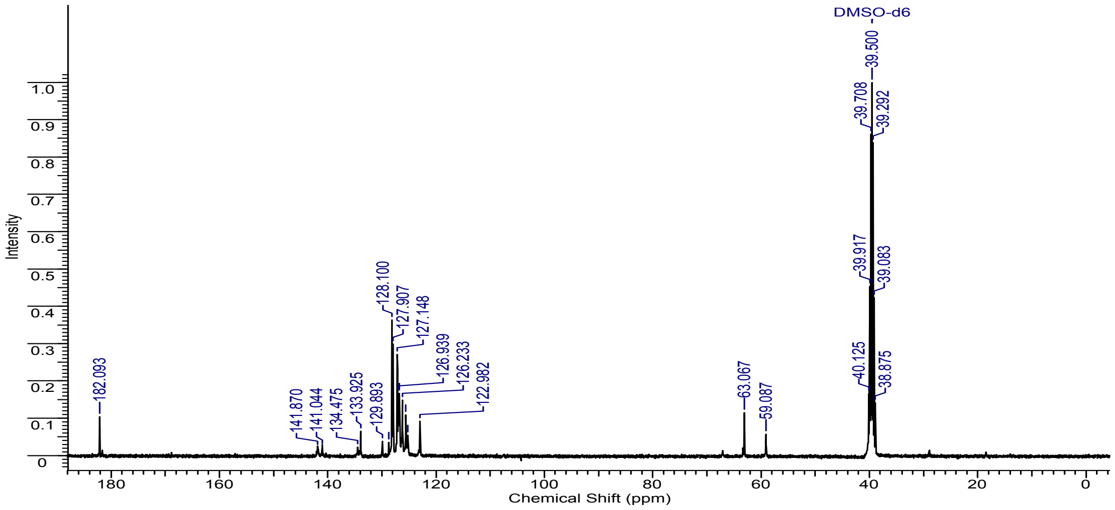

## 1-[2-(1-Ethylpropylamino)-1,2-diphenylethyl]-3-phenylthiourea (1b)

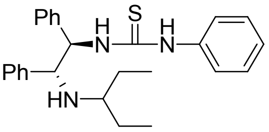


### Figure S3. $^{1}$H nmr

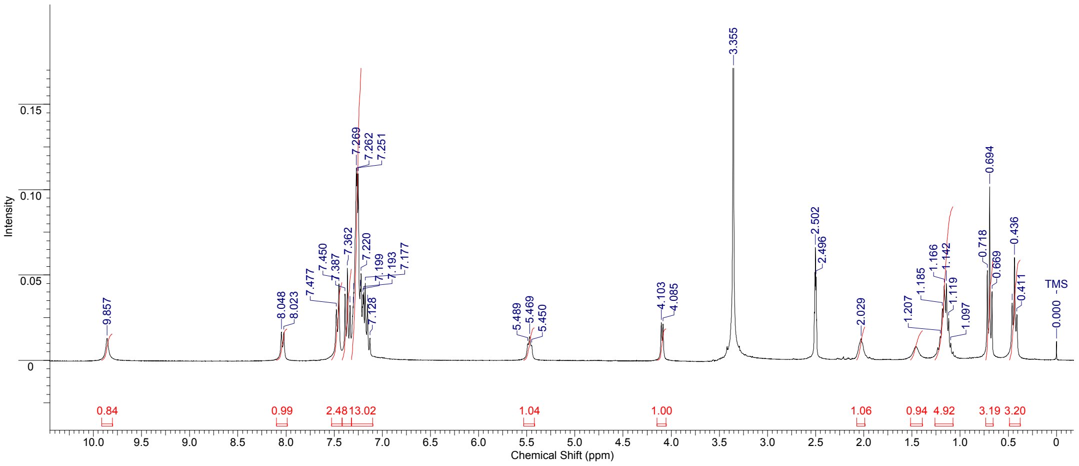


### Figure S4. $^{13}$C nmr

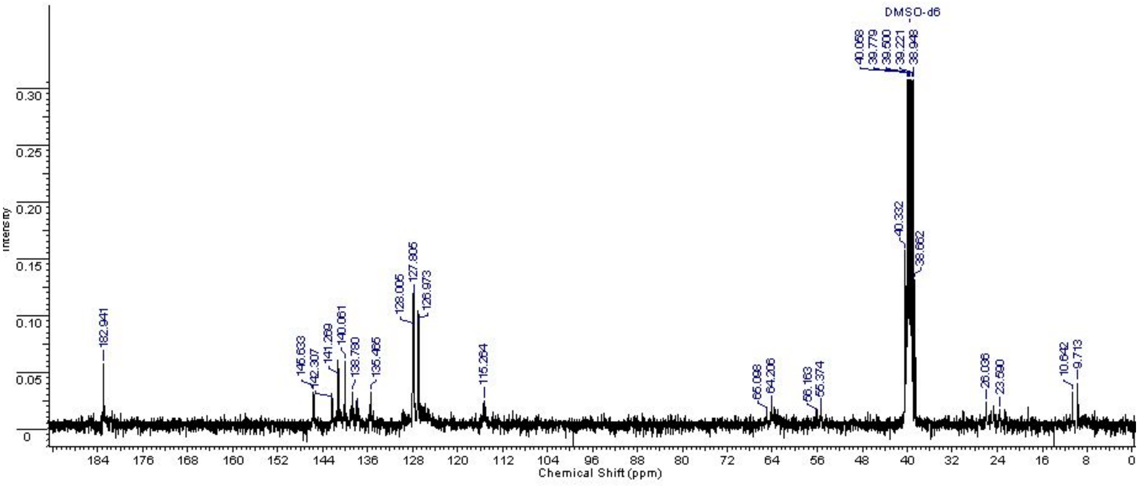

**1-[2-(1-Methylethylamino)-1,2-diphenylethyl]-3-(4-nitrophenyl)thiourea (1c)**

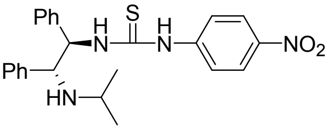


**Figure S5.** [1]**H nmr**

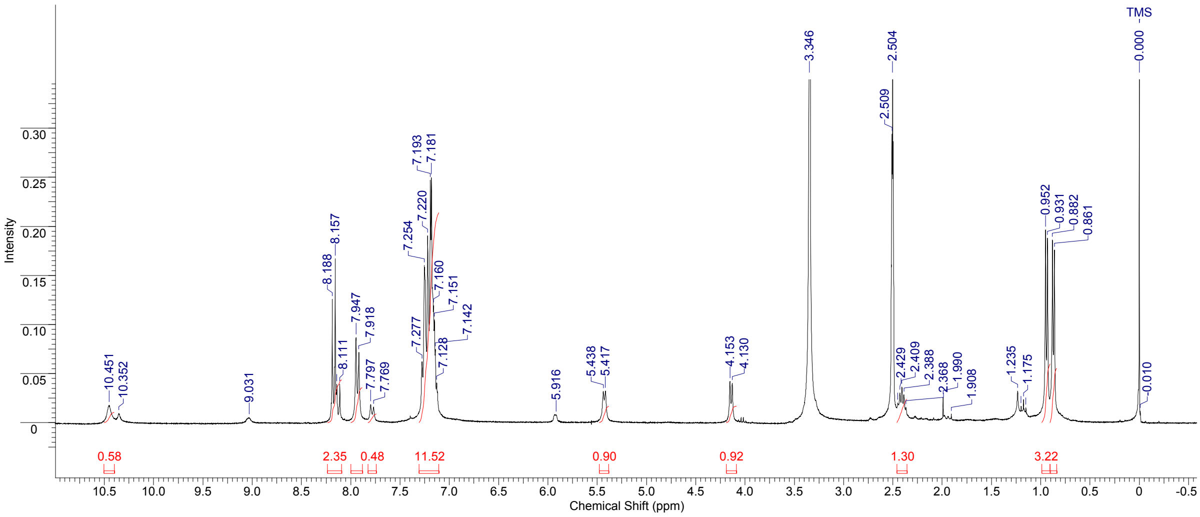

**Figure S6.** [13]**C nmr**

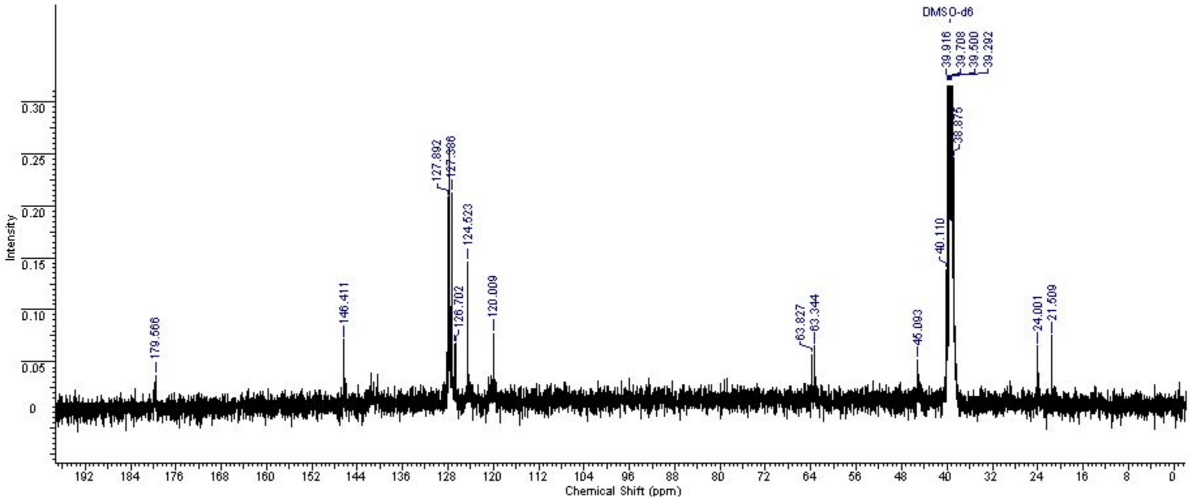

**(*R*,*R*)-1-[2-(1-Ethylpropylamino)-1,2-diphenylethyl]-3-(4-fluorophenyl) thiourea (1d)**

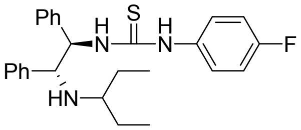


**Figure S7. $^{1}$H nmr**

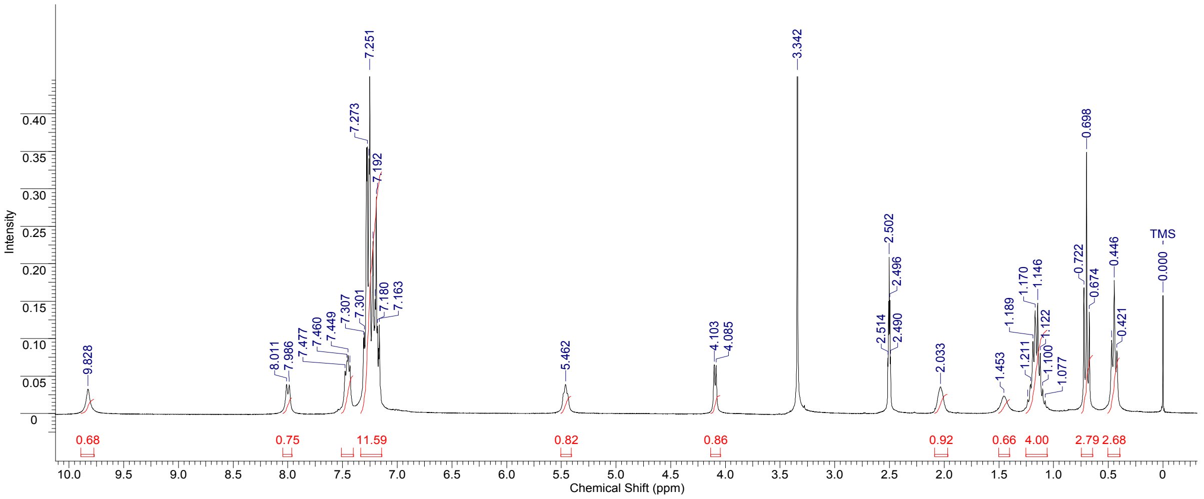


**Figure S8. $^{13}$C nmr**

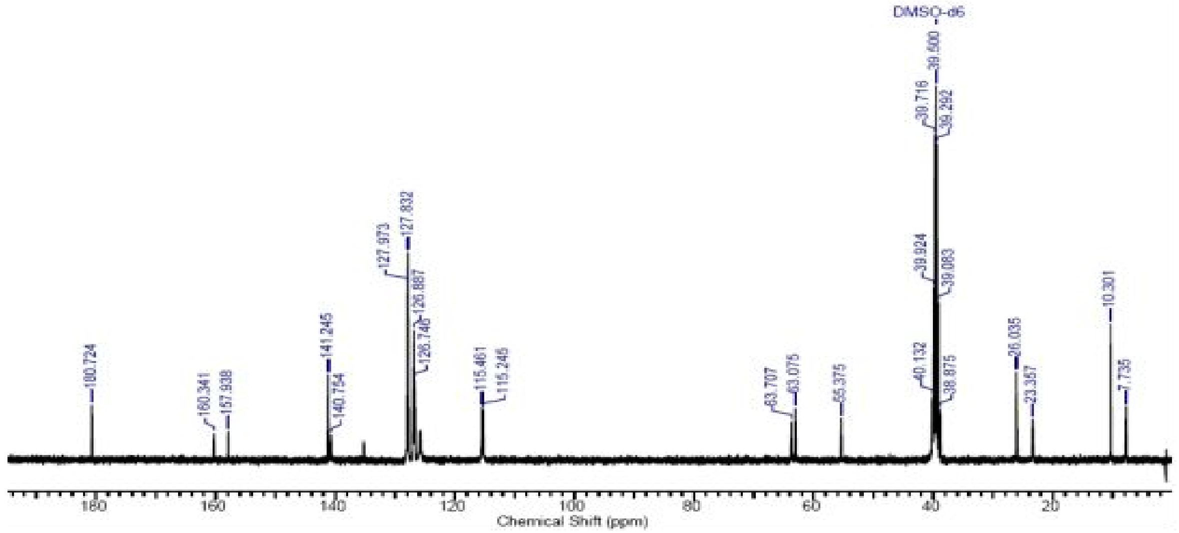

### (*R*,*R*)-*N*-[2-(1-Ethylpropylamino)-1,2-diphenylethyl]-*N′*-(4-methoxyphenyl) thiourea (1e)

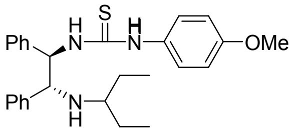


**Figure S9. [1]H nmr**

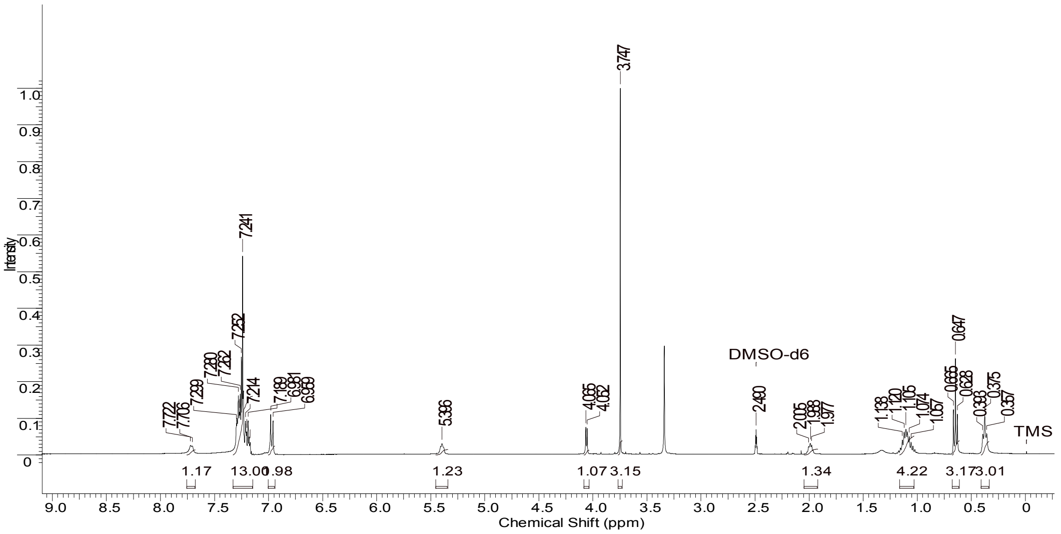


**Figure S10. [13]C nmr**

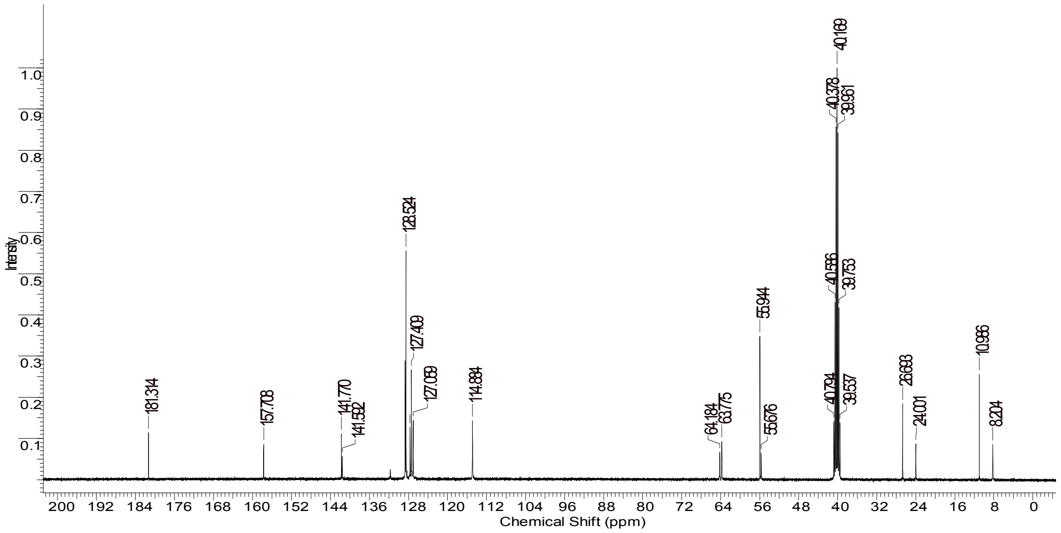

**(*R*,*R*)-1-[2-(1-Ethylpropylamino)-1,2-diphenylethyl]-3-(4-methylphenyl)thiourea (1f)**

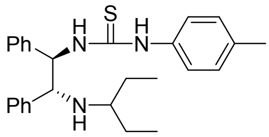


**Figure S11. $^{1}H$ nmr**

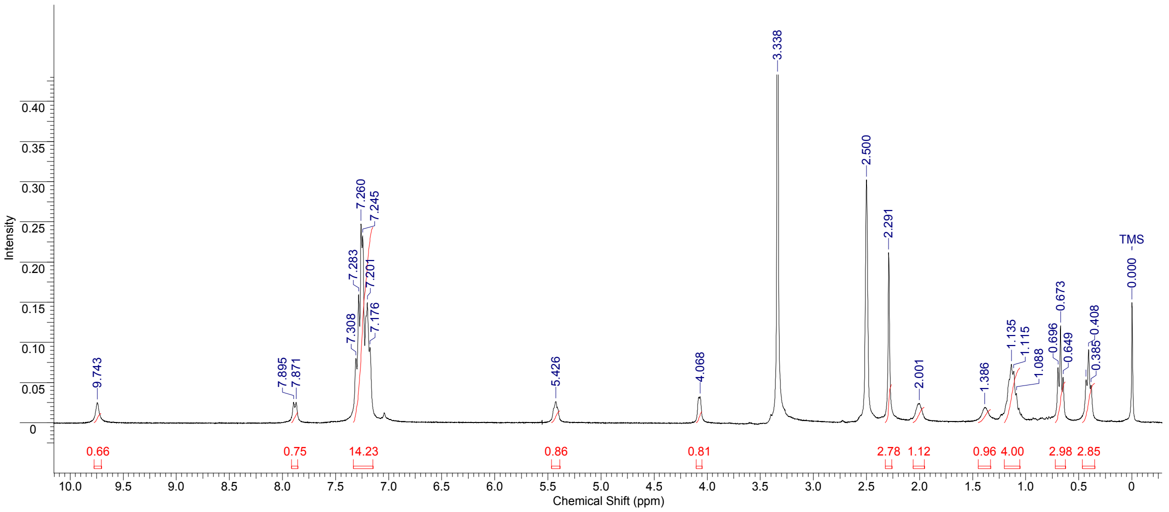


**Figure S12. $^{13}C$ nmr**

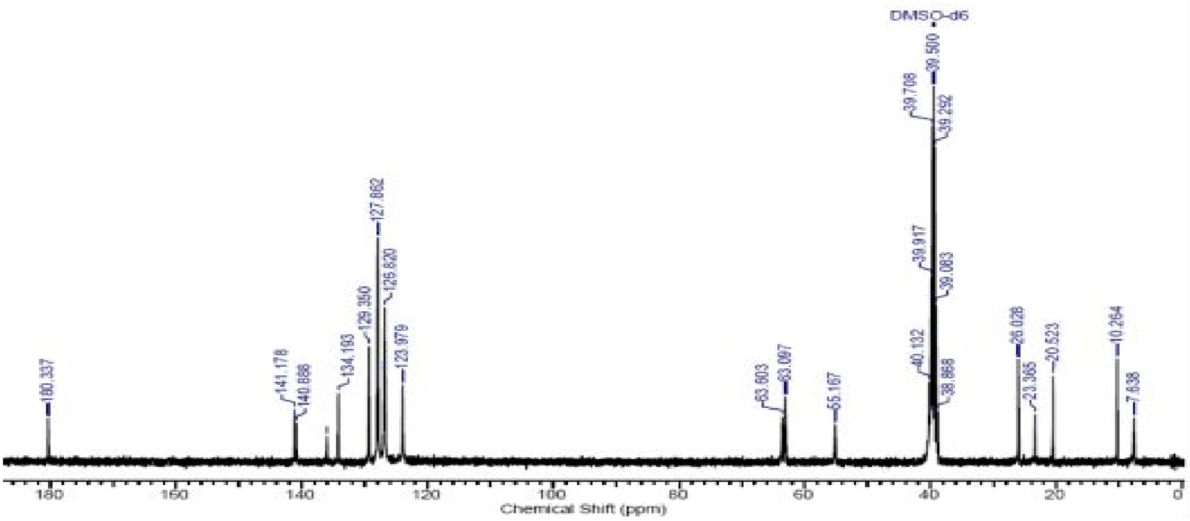

**(*R*,*R*)-1-[2-(1-Ethylpropylamino)-1,2-diphenylethyl]-3-[4-(trifluoromethy)phenyl]thiourea (1g)**

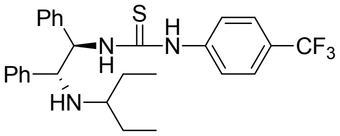


**Figure S13. $^{1}$H nmr**

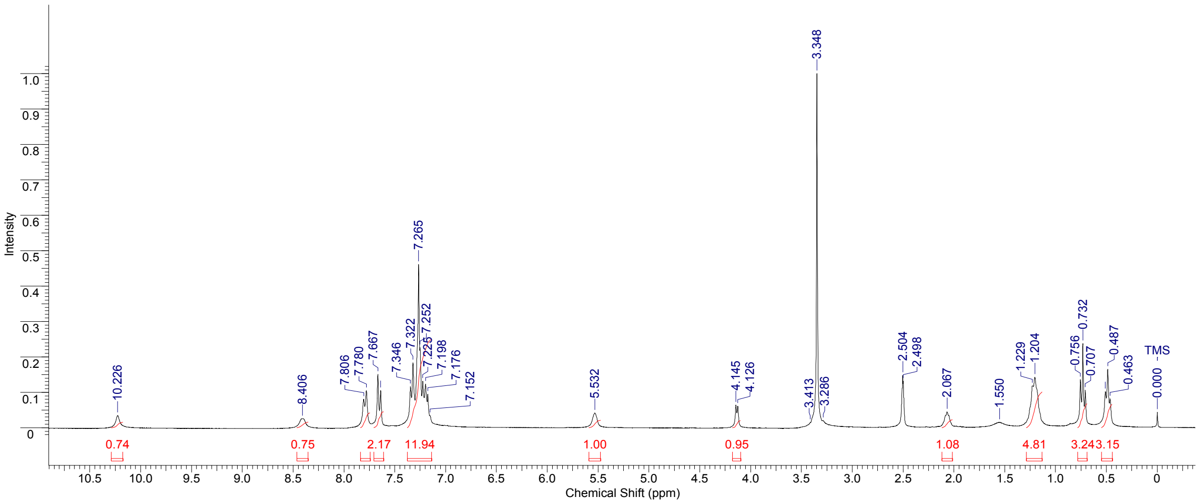

**Figure S14. $^{13}$C nmr**

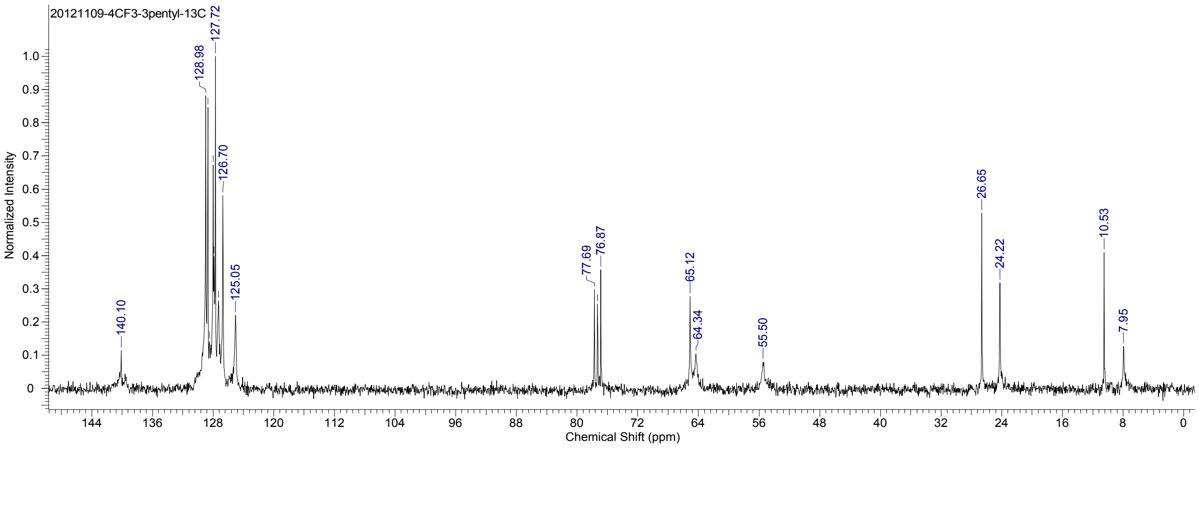

**(*R*,*R*)-1-[2-(1-Ethylpropylamino)-1,2-diphenylethyl]-3-pentafluorophenylthiourea (1h)**

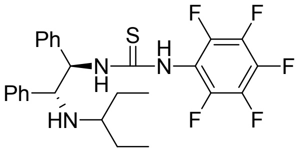

**Figure S15. $^{1}$H nmr**

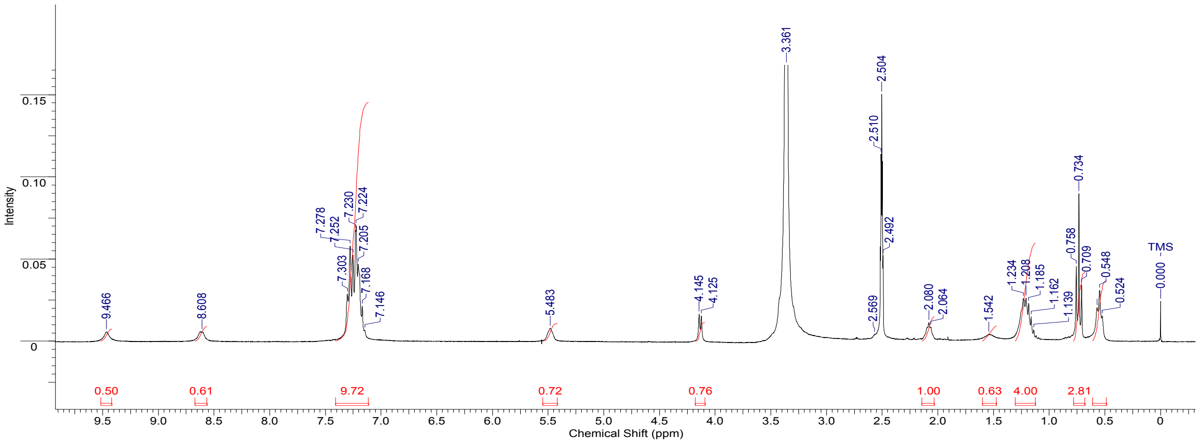

**Figure S16. $^{13}$C nmr**

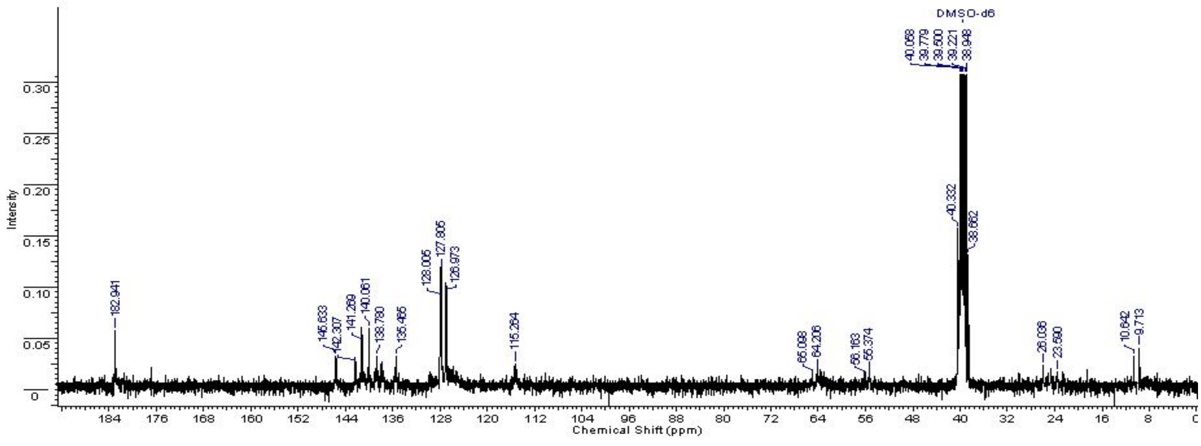

## 1-[2-(1-Propylbutylamino)-1,2-diphenylethyl]-3-(4-nitrophenyl)thiourea (1i)

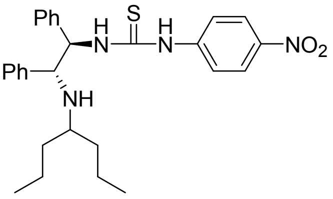


**Figure S17.** $^{1}H$ **nmr**

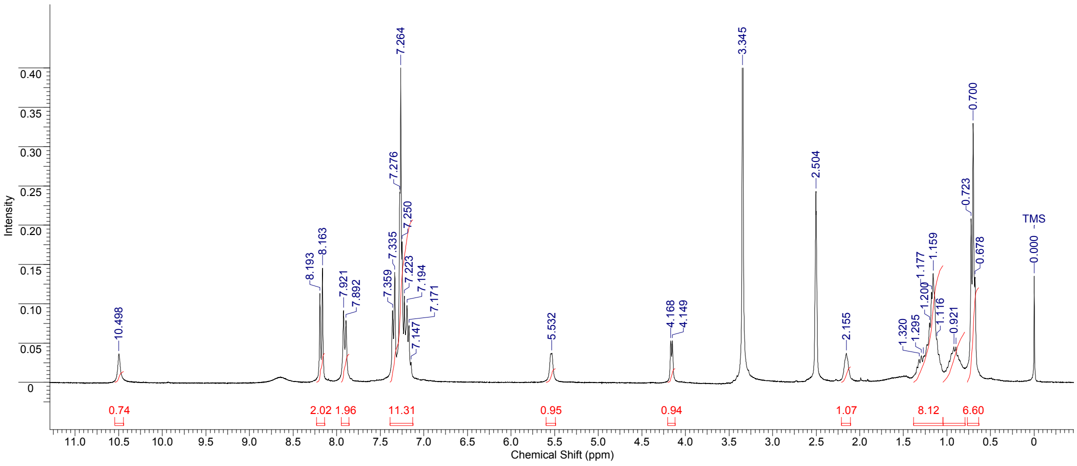

**(*R*,*R*)-1-[2-(1-Ethylpropylamino)-1,2-diphenylethyl]-3-[3,5-bis(trifluoromethyl)phenyl]-thiourea (1j)**

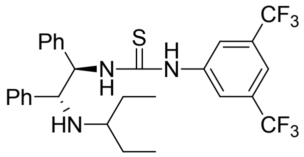

**Figure S18. [1]H nmr**

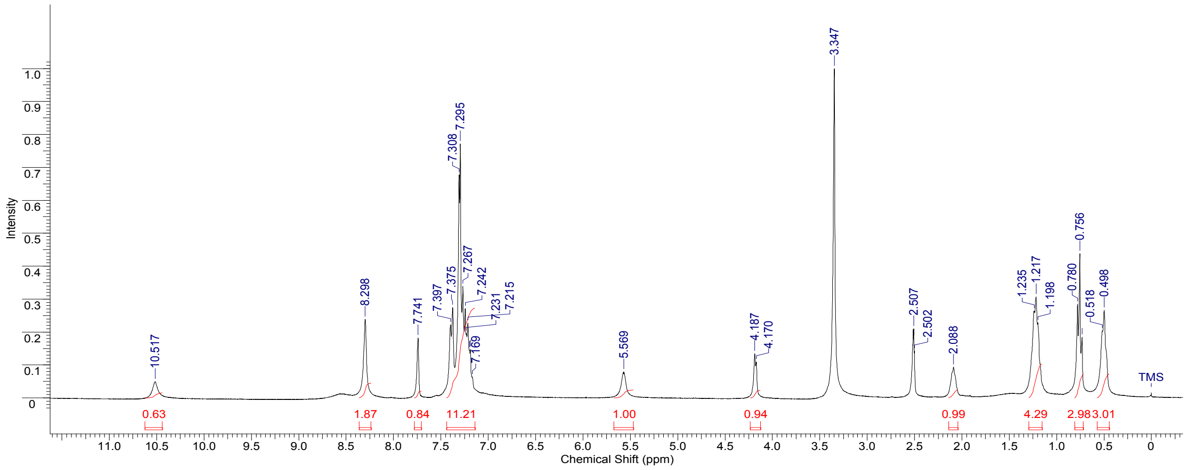

**Figure S19. [13]C nmr**

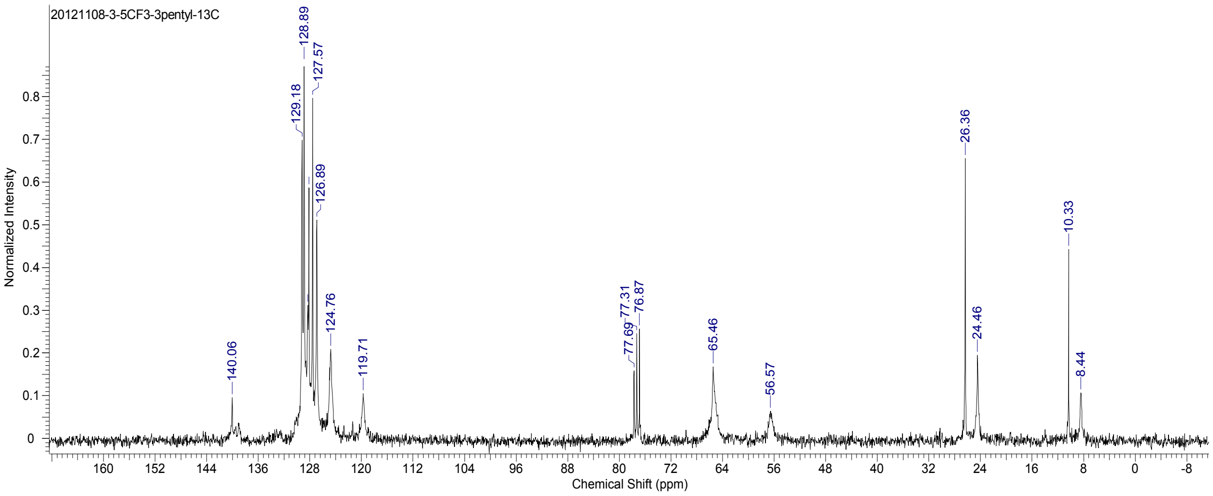

## (*R*,*R*)-1-[2-(1-Ethylpropylamino)-1,2-diphenylethyl]-3-(4-nitrophenyl)thiourea (1k)

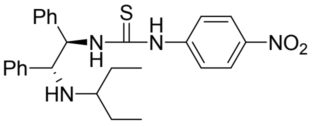


### Figure S20. [1]H nmr

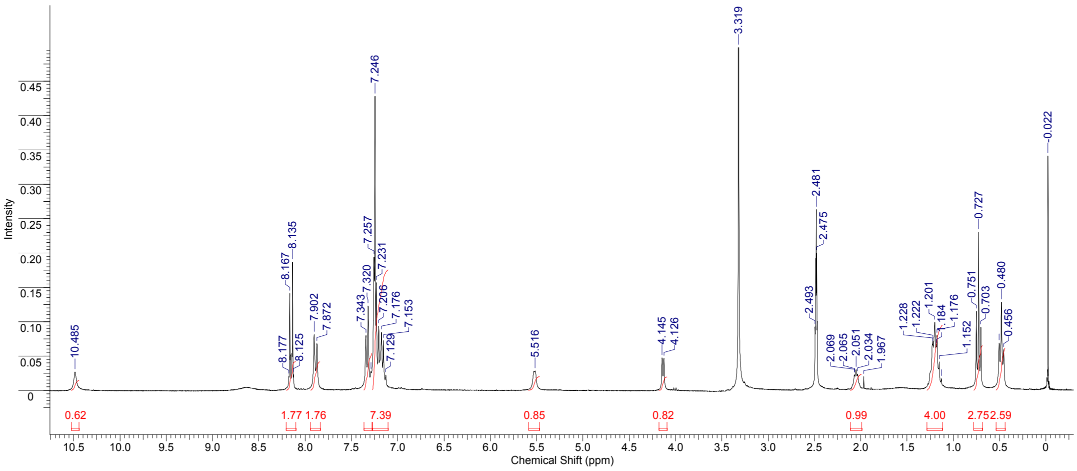


### Figure S21. [13]C nmr

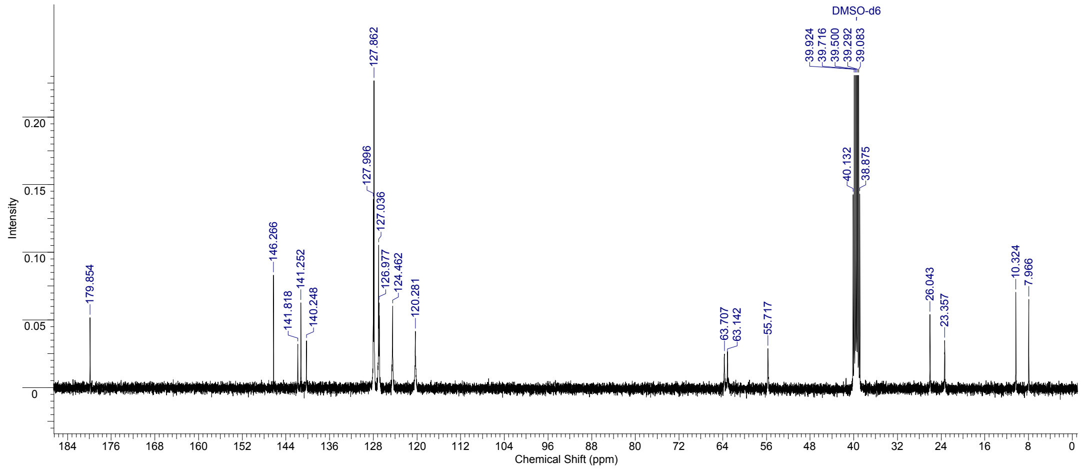

**(*R*,*R*)-1-[2-(1-Ethylpropylamino)-1,2-diphenylethyl]-3-(4-cyanophenyl)thiourea (1l)**

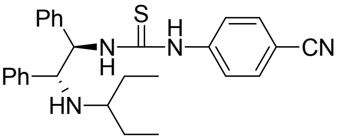


**Figure S22. $^{1}$H nmr**

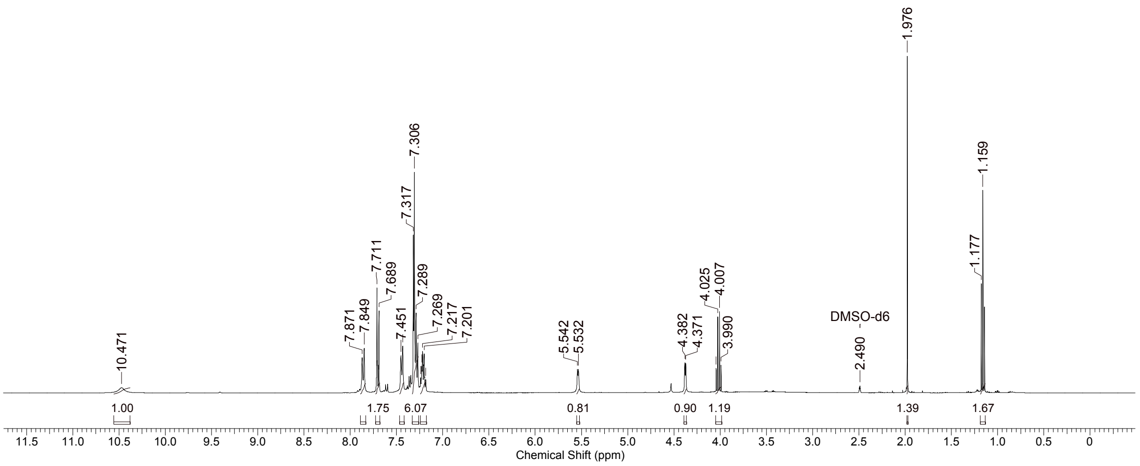


**Figure S23. $^{13}$C nmr**

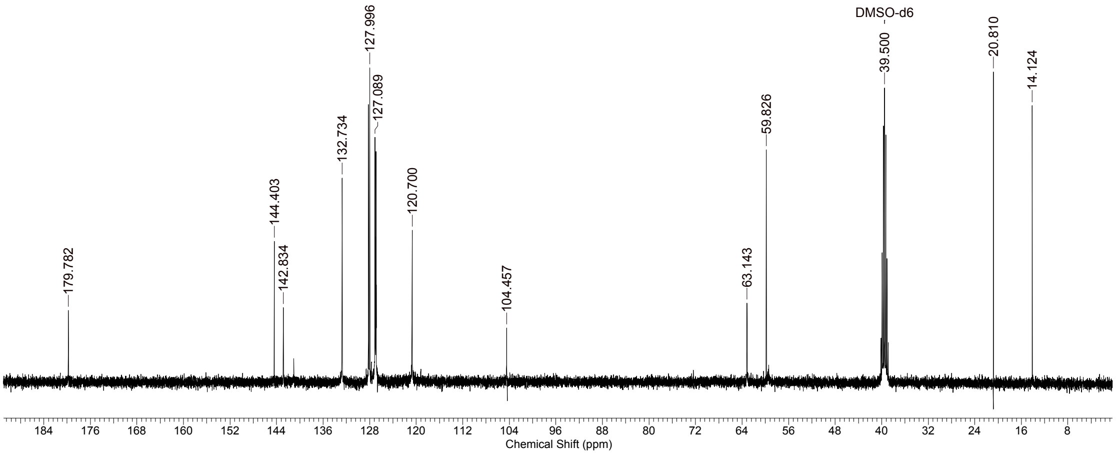

**1-[(1*R*,2*R*)-2-Amino-1,2-diphenylethyl]-3-[3,5-Bis(trifluoromethyl)phenyl]thiourea (1m)**

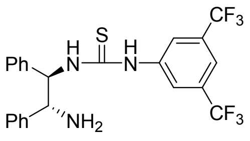


**Figure S24.** $^{1}$**H nmr**

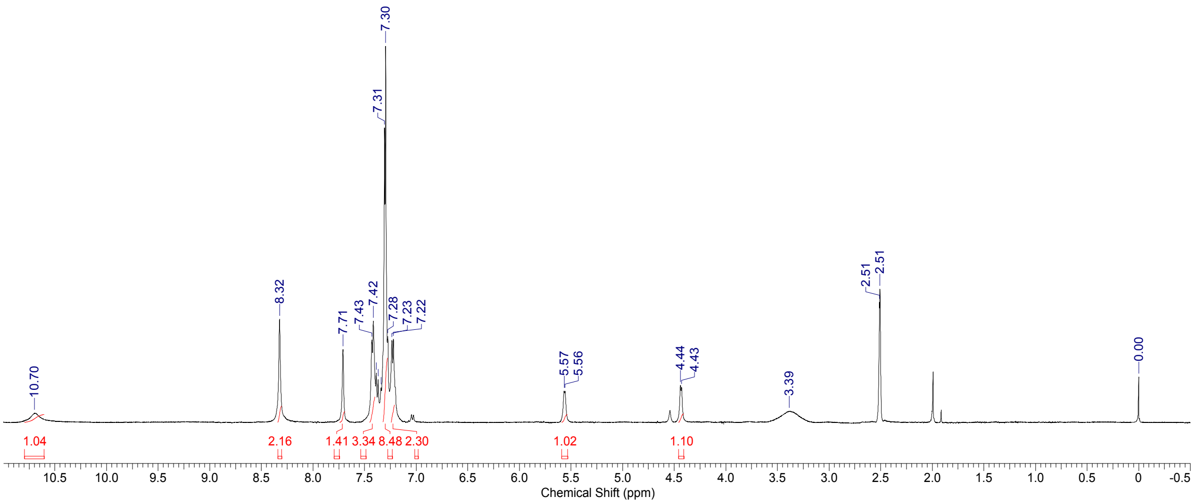


**Figure S25.** $^{13}$**C nmr**

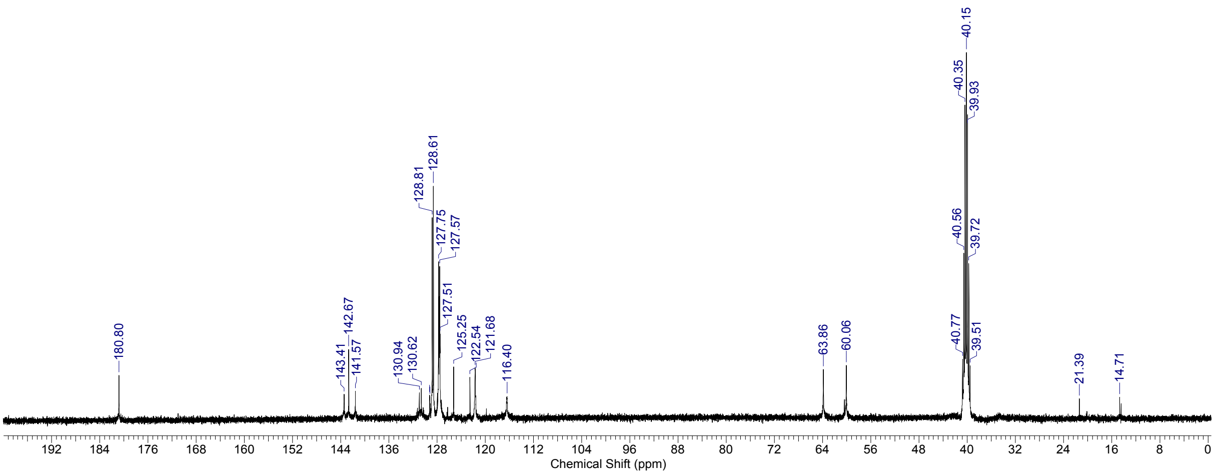

## 1-(2-Amino-1,2-diphenylethyl)-3-p-tolylthiourea (1n)

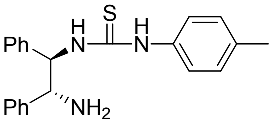


**Figure S26. [1]H nmr**

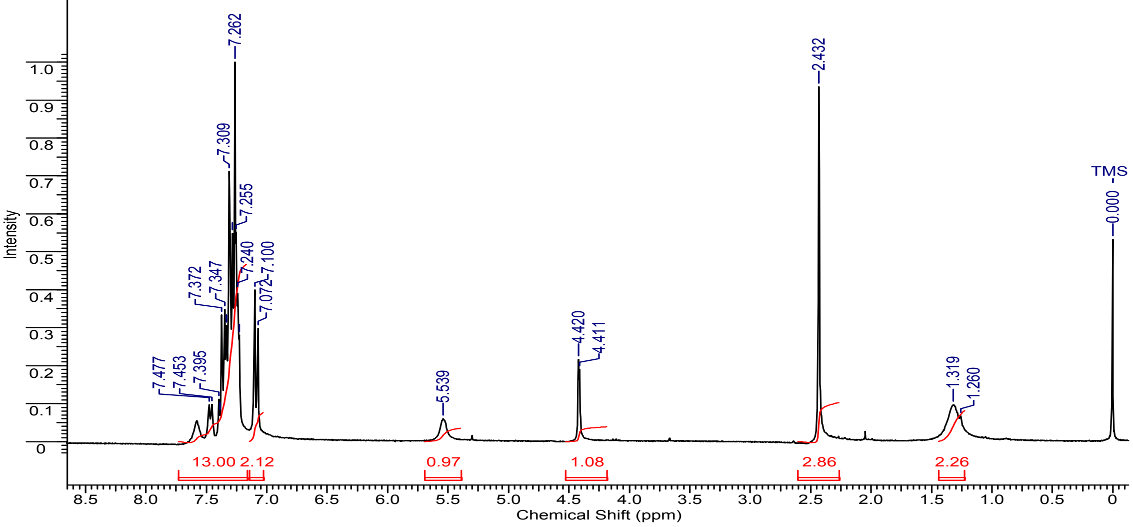


**Figure S27. [13]C nmr**

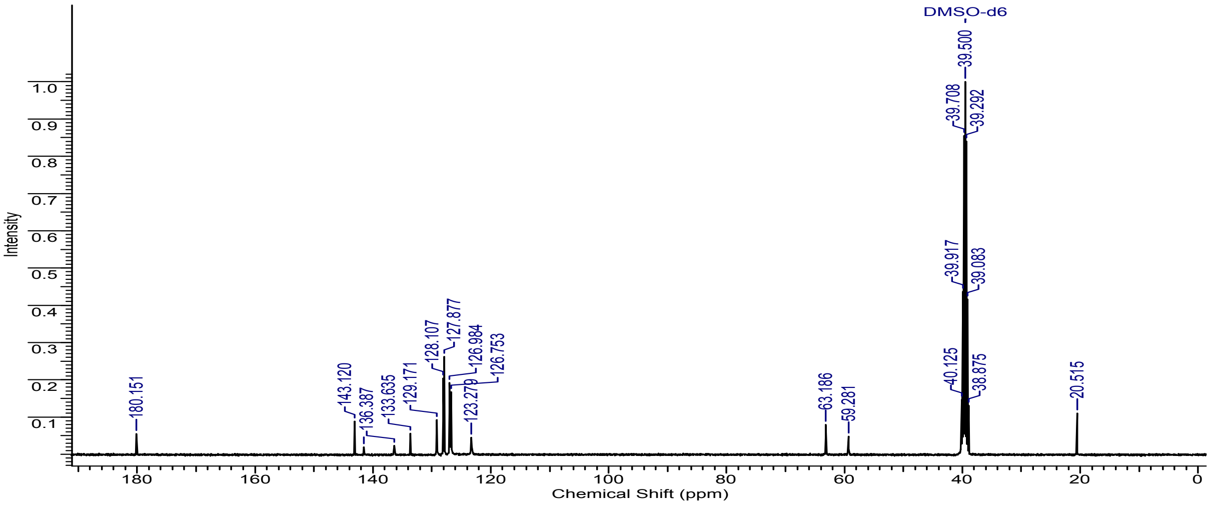

## 1-(2-Amino-1,2-diphenylethyl)-3-naphthalen-1-ylthiourea (1o)

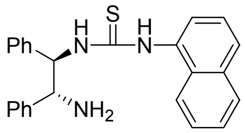


**Figure S28. $^{1}$H nmr**

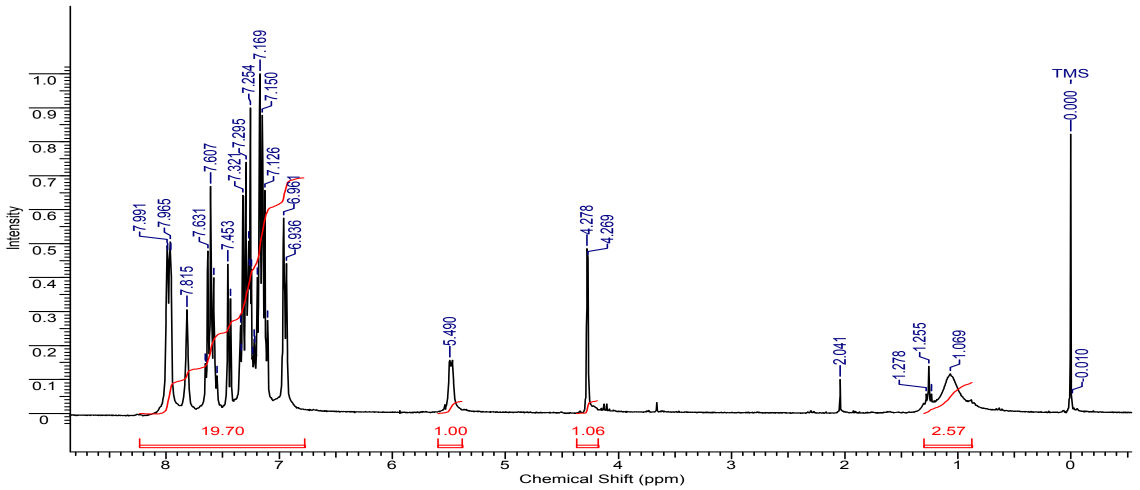


**Figure S29. $^{13}$C nmr**

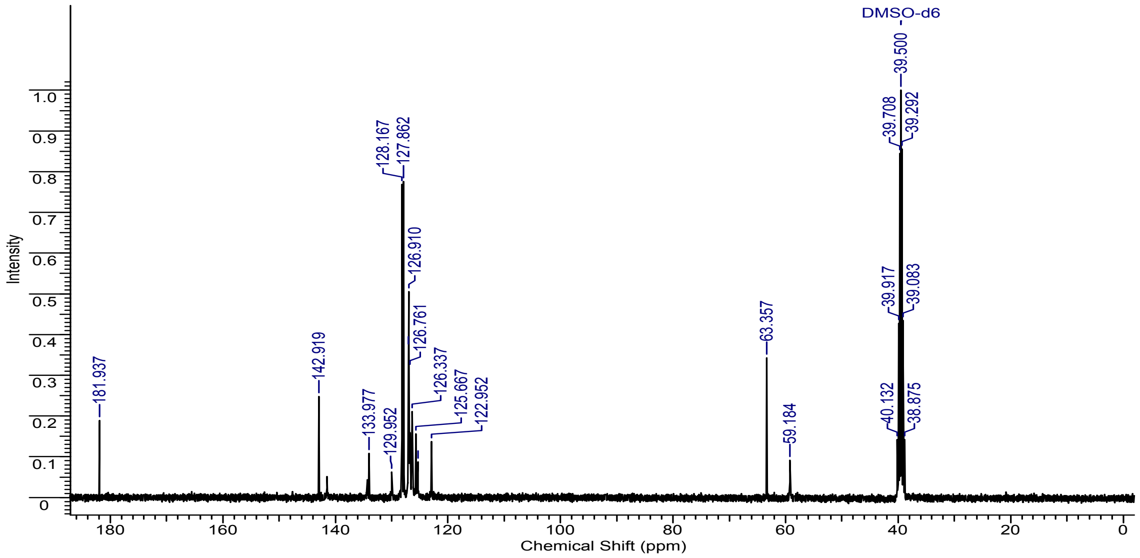

## 1-(2-Amino-1,2-diphenylethyl)-3-(2-fluorophenyl)thiourea (1p)

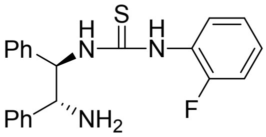


### Figure S30. $^{1}$H nmr

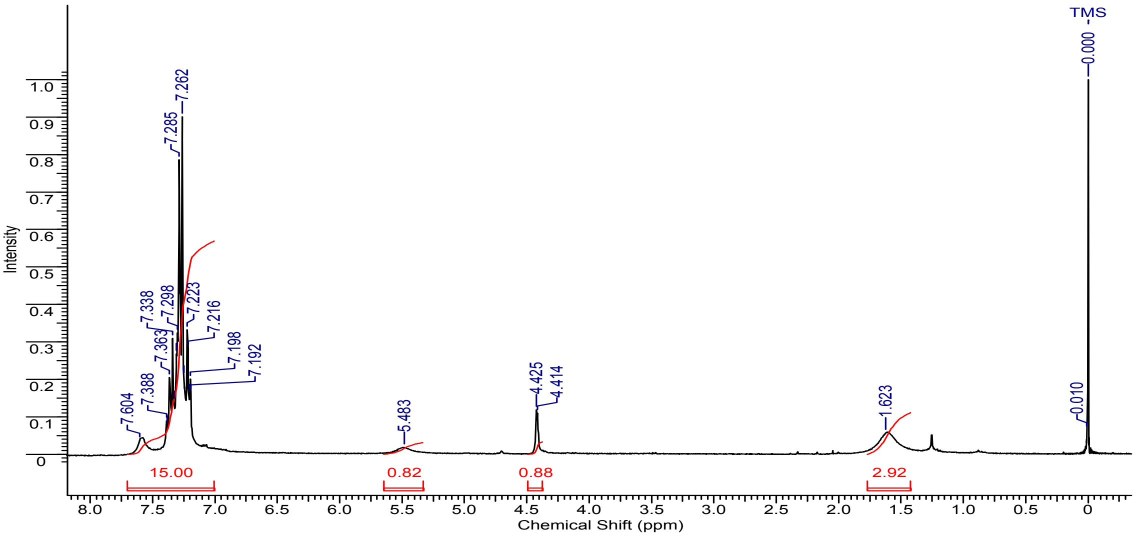


### Figure S31. $^{13}$C nmr

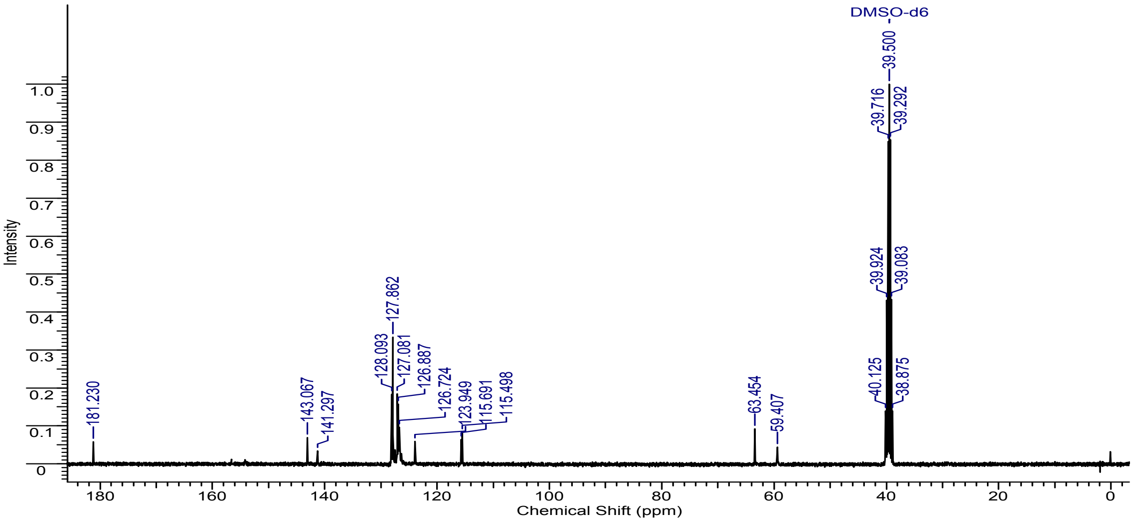

**1-(2-Amino-1,2-diphenylethyl)-3-(4-nitrophenyl)thiourea (1q)**

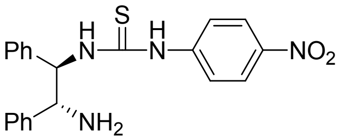

**Figure S32. [1]H nmr**

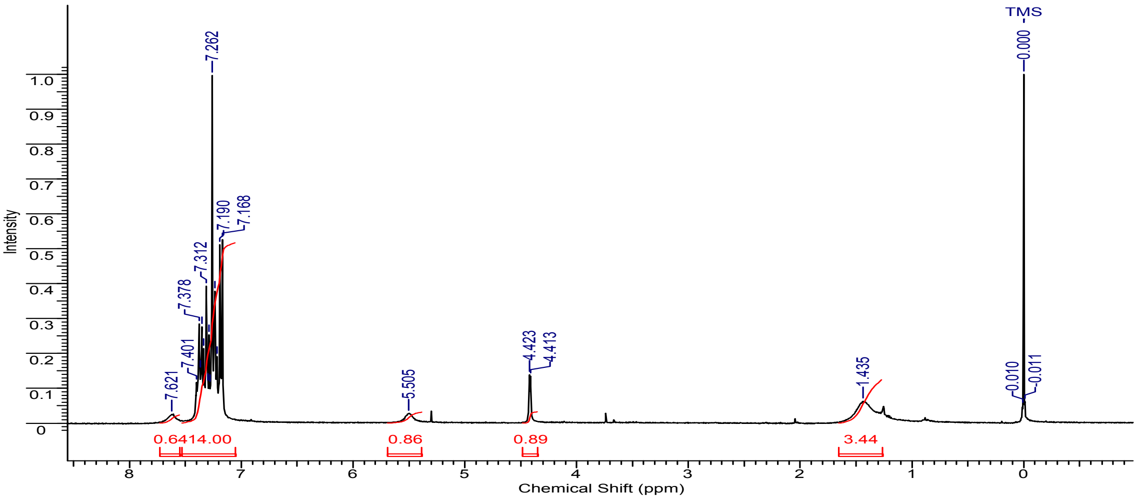

**Figure S33. [13]C nmr**

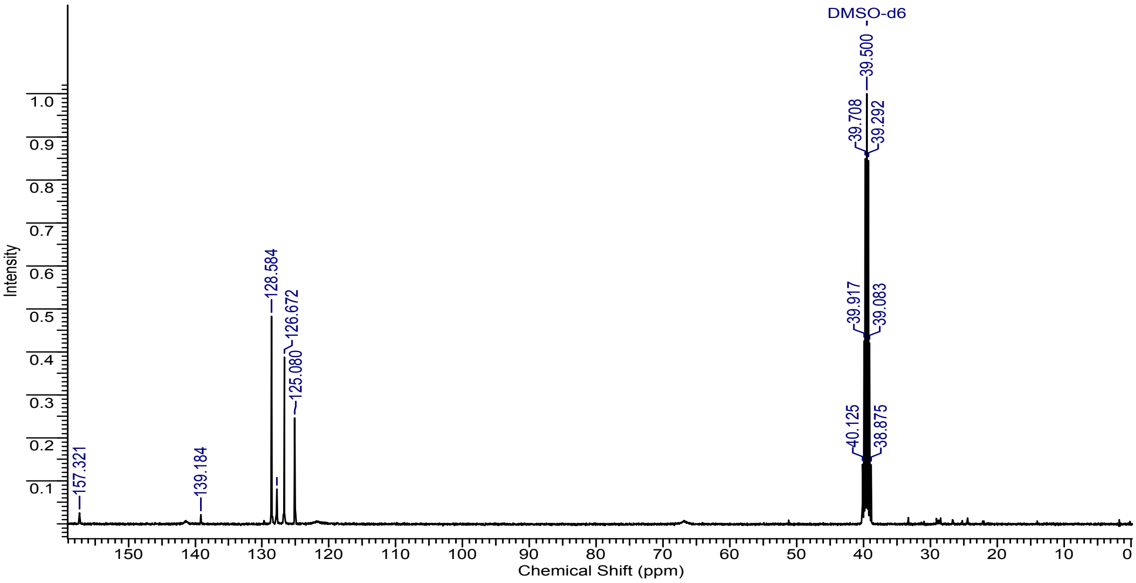

**1-(2-Amino-1,2-diphenylethyl)-3-(4-methoxyphenyl)thiourea (1r)**

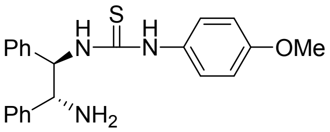


**Figure S34. [1]H nmr**

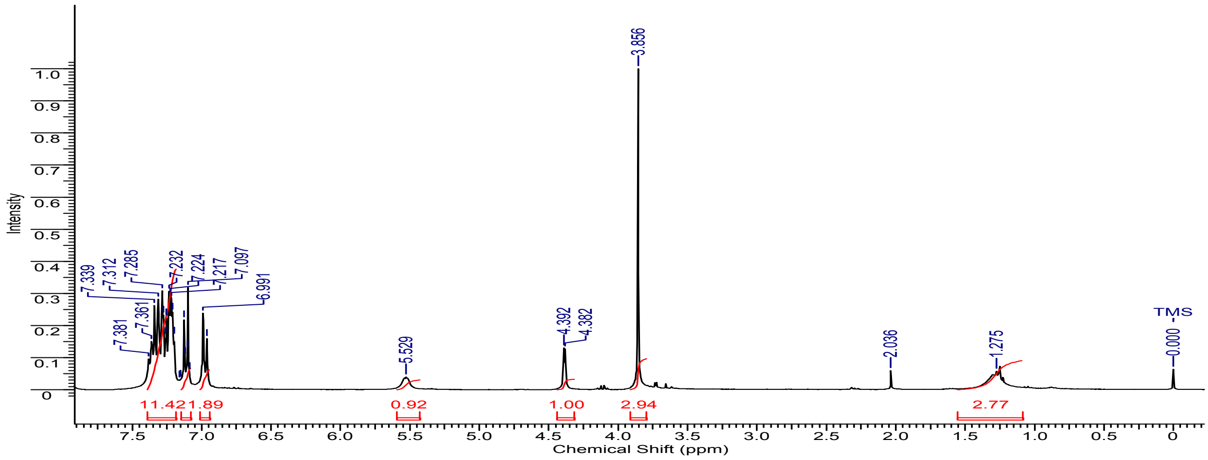


**Figure S35. [13]C nmr**

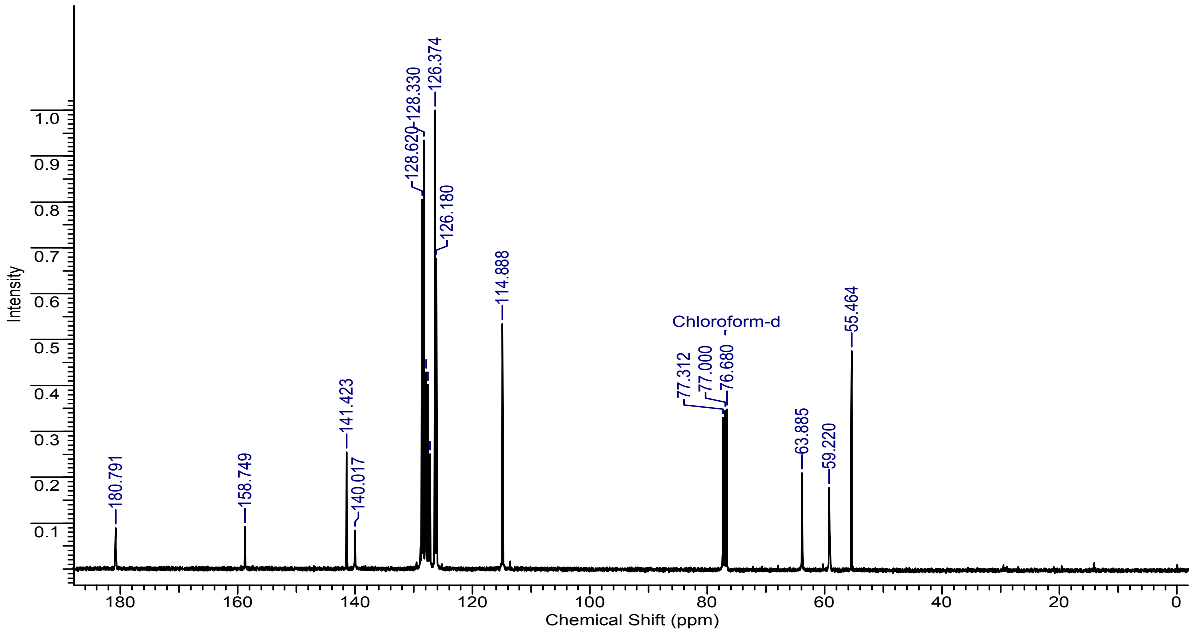

**1-(2-Amino-1,2-diphenylethyl)-3-(2,6-dimethylphenyl)thiourea (1s)**

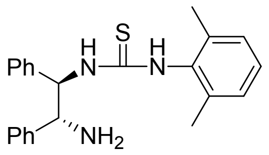


**Figure S36. $^{1}$H nmr**

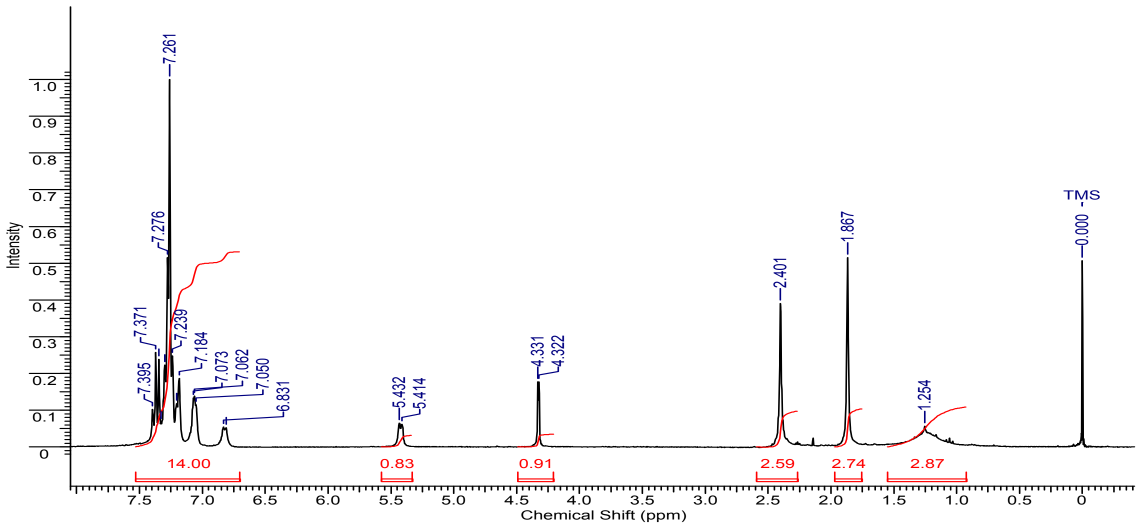


**Figure S37. $^{13}$C nmr**

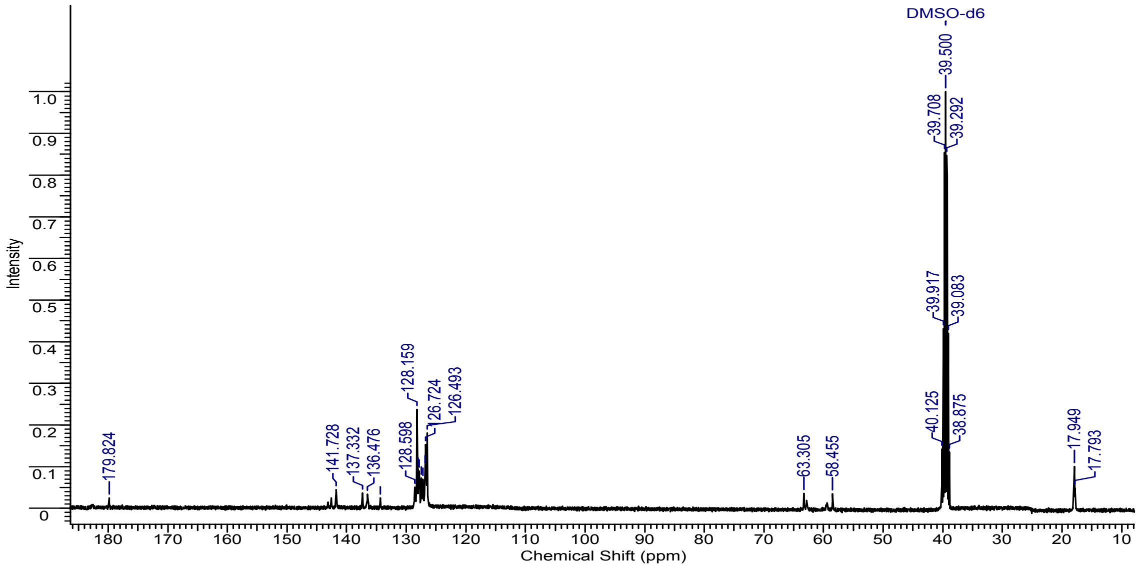

## 1-(2-Amino-1,2-diphenylethyl)-3-(4-fluorophenyl)thiourea (1t)

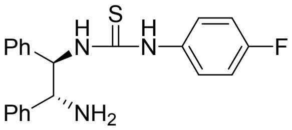


**Figure S38. [1]H nmr**

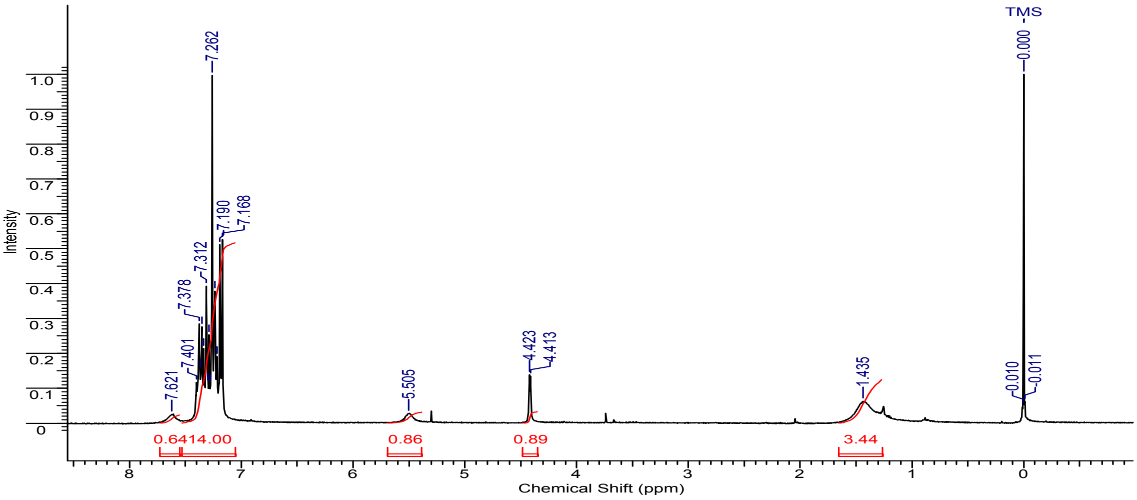


**Figure S39. [13]C nmr**

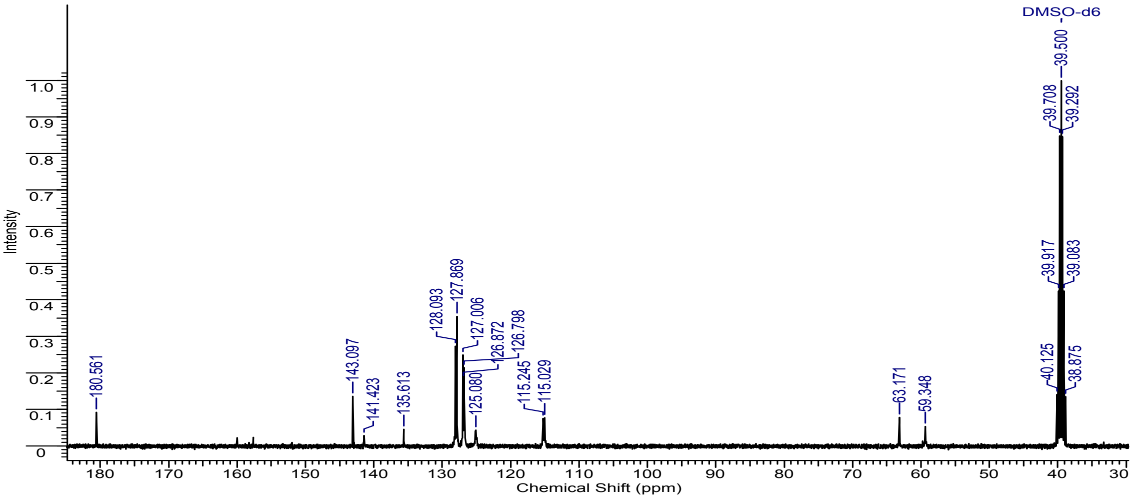

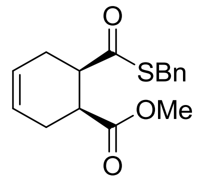


(2a)

**Figure S40.** $^{1}$H nmr

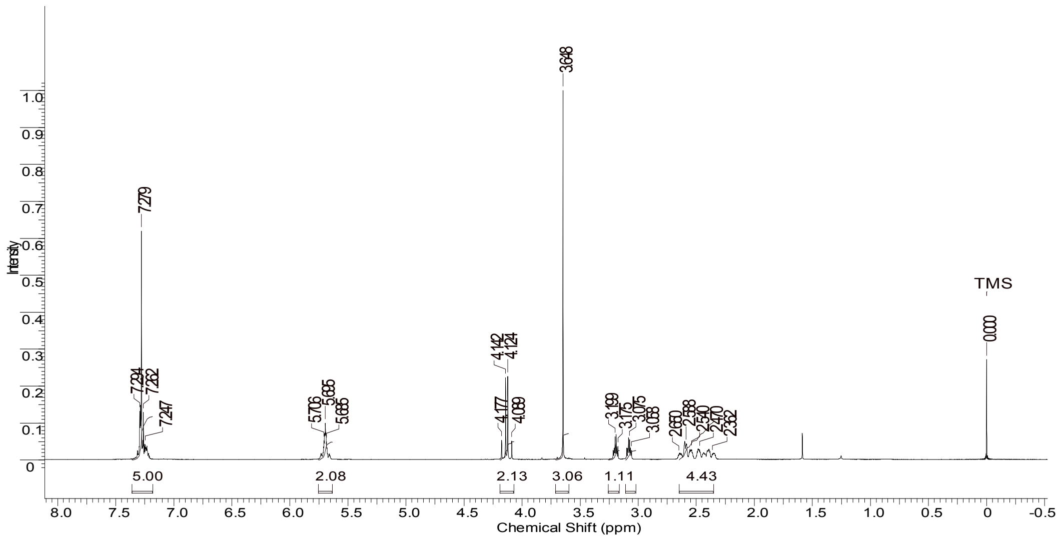


**Figure S41.** $^{13}$C nmr

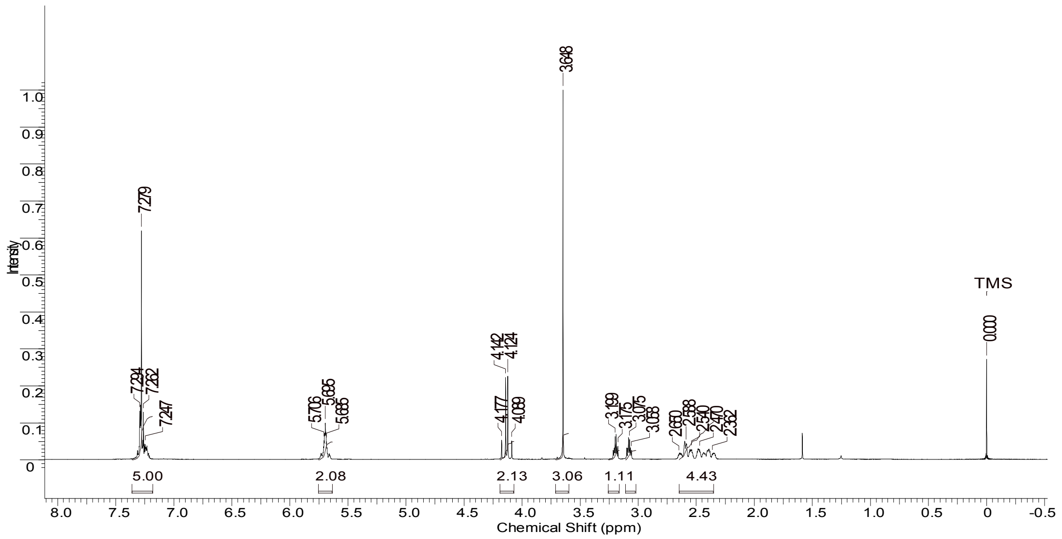


**Figure S42.** Mass

```
Inlet : Direct                        Ion Mode : FAB+
RT : 2.17 min                         Scan#: 27
Elements : C 16/0, H 19/0, O 3/0, S 1/0
Mass Tolerance      : 10mmu
Unsaturation (U.S.) : 0.0 - 100.0

Observed m/z   Int%
  291.1057     100.0
Estimated m/z  Error[ppm]  U.S.    C     H     O     S
  291.1055       +0.9       8.5    16    19    3     1
```

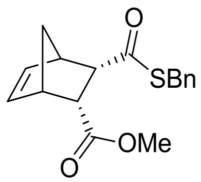


(2b)

**Figure S43.** $^{1}$H nmr

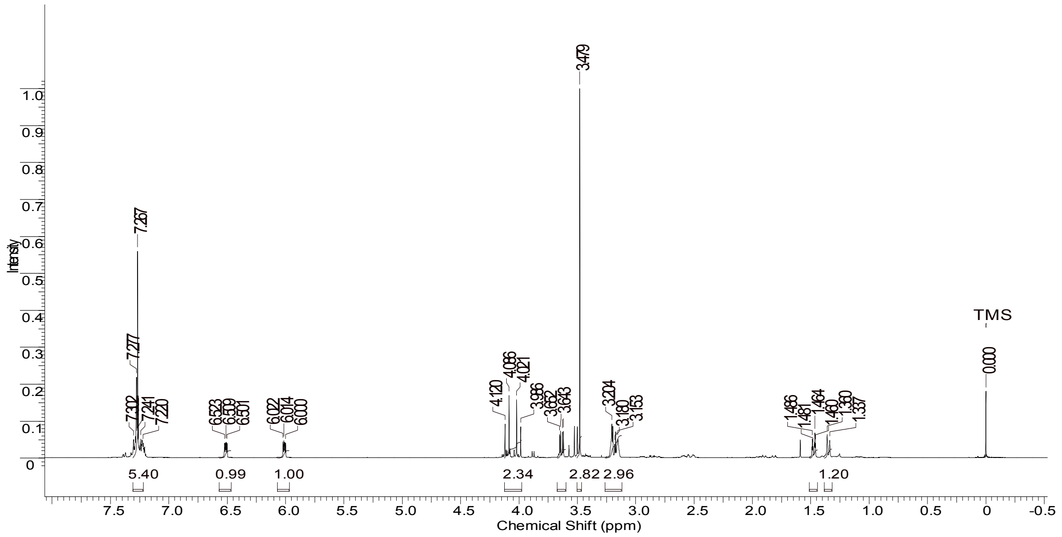

**Figure S44.** $^{13}$C nmr

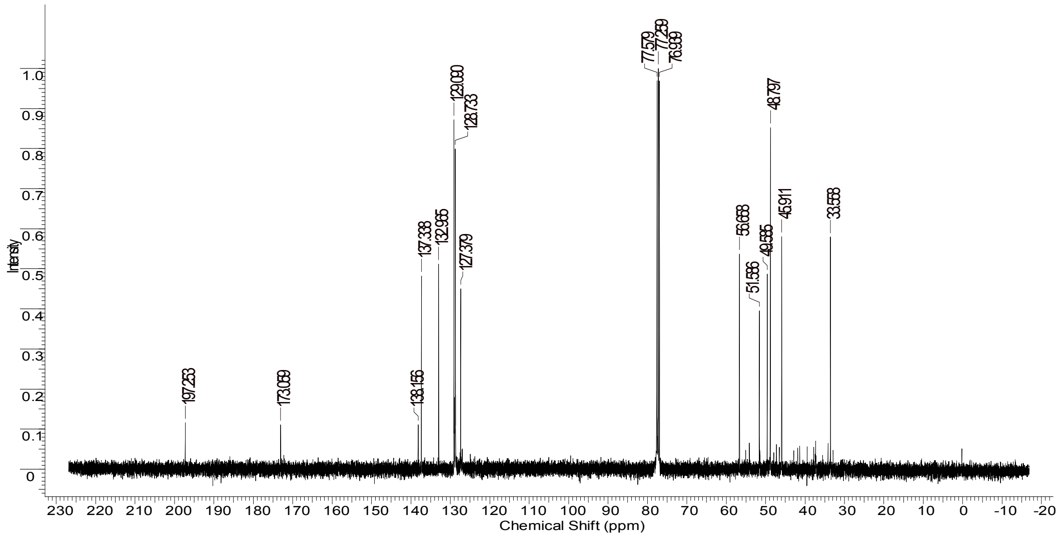

**Figure S45.** Mass

```
Inlet : Direct                          Ion Mode : FAB+
RT : 2.00 min                           Scan#: 25
Elements : C 17/0, H 19/0, O 3/0, S 1/0
Mass Tolerance       : 10mmu
Unsaturation (U.S.) : 0.0 - 100.0

Observed m/z    Int%
  303.1053      100.0
Estimated m/z   Error[ppm]   U.S.     C     H     O     S
  303.1055        -0.8        9.5    17    19     3     1
```

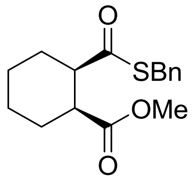

(2c)

**Figure S46.** $^{1}H$ nmr

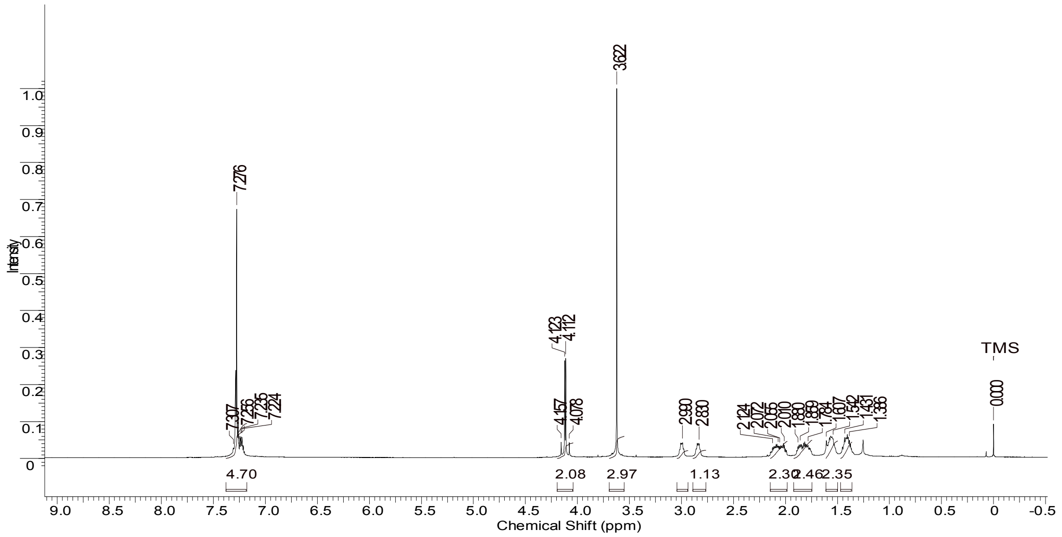

**Figure S47.** $^{13}C$ nmr

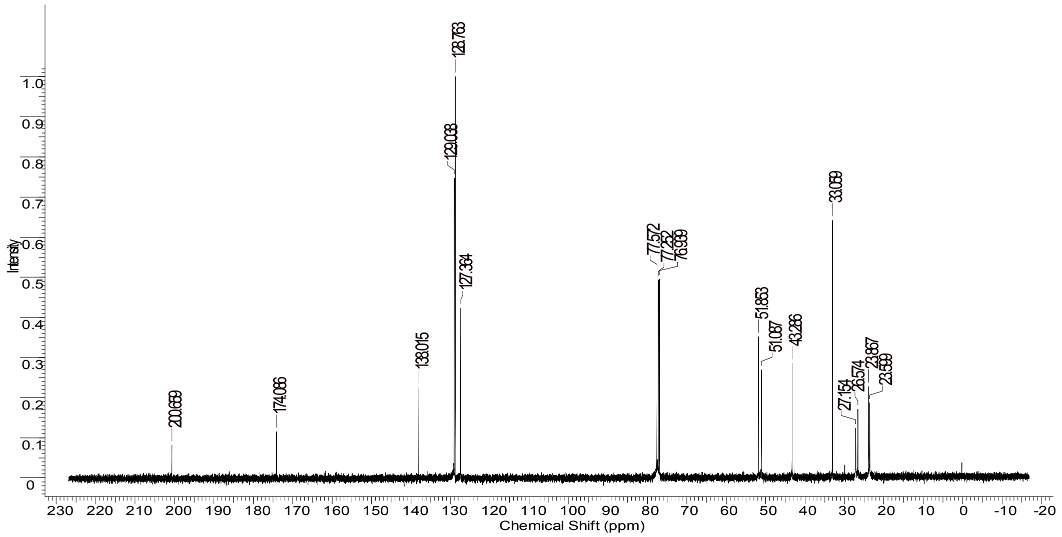

**Figure S48.** Mass

Inlet : Direct  Ion Mode : FAB+
RT : 2.84 min  Scan#: 35
Elements : C 16/0, H 21/0, O 3/0, S 1/0
Mass Tolerance : 10mmu
Unsaturation (U.S.) : 0.0 - 100.0

| Observed m/z | Int% | | | | | |
|---|---|---|---|---|---|---|
| 293.1211 | 100.0 | | | | | |
| Estimated m/z | Error[ppm] | U.S. | C | H | O | S |
| 293.1211 | -0.2 | 7.5 | 16 | 21 | 3 | 1 |

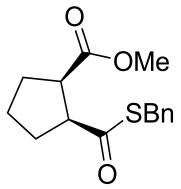

(2d)

**Figure S49.** $^{1}$H nmr

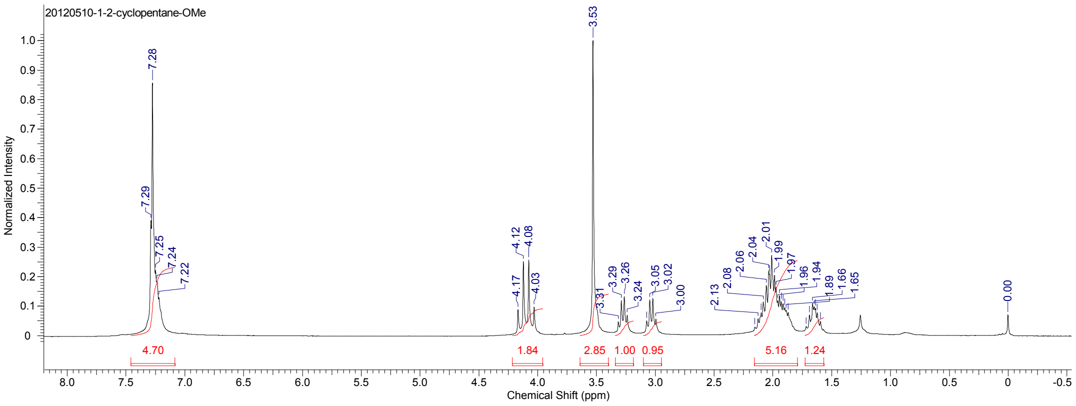


**Figure S50.** $^{13}$C nmr

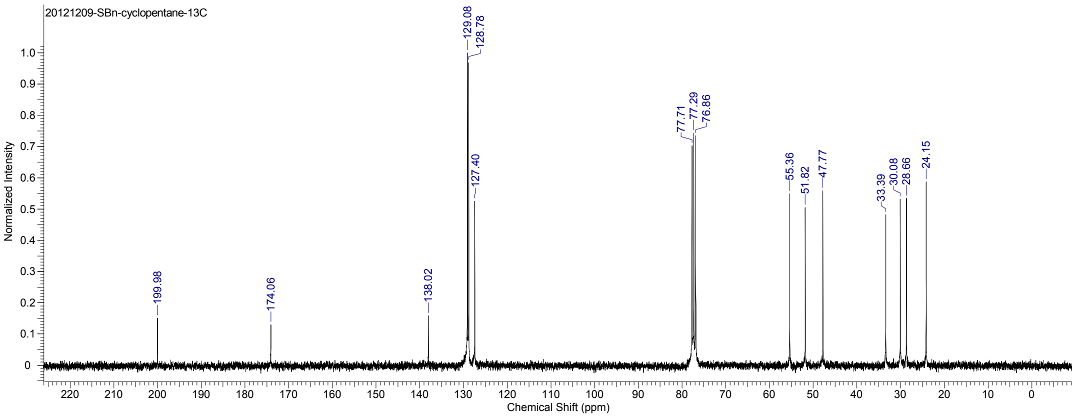

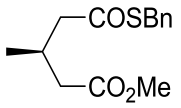


(2e)

**Figure S51.** $^{1}H$ nmr

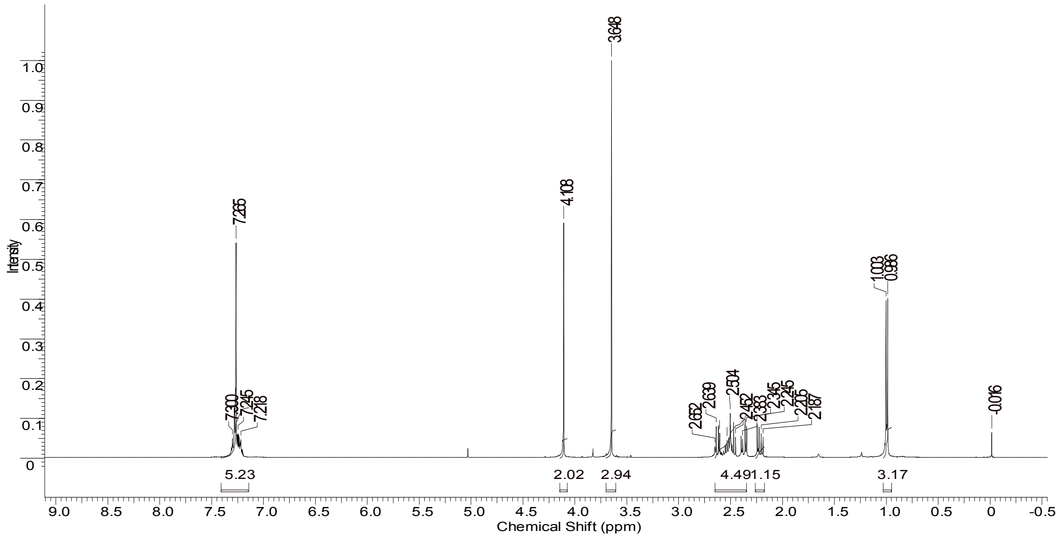


**Figure S52.** $^{13}C$ nmr

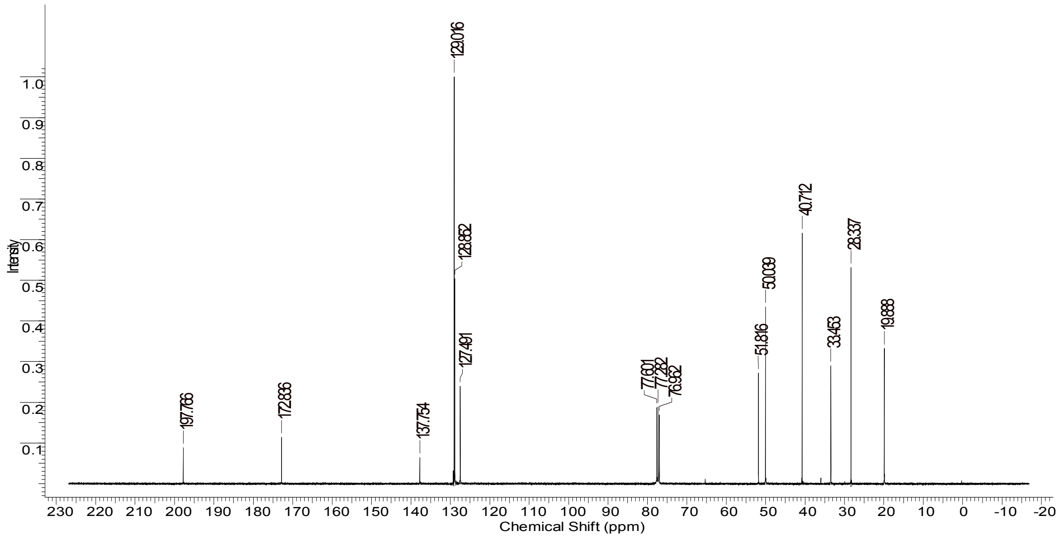


**Figure S53.** Mass

Inlet : Direct    Ion Mode : FAB+
RT : 2.00 min    Scan#: 25
Elements : C 14/0, H 19/0, O 3/0, S 1/0
Mass Tolerance : 10mmu
Unsaturation (U.S.) : 0.0 - 100.0

| Observed m/z | Int% | | | | | |
|---|---|---|---|---|---|---|
| 267.1051 | 100.0 | | | | | |
| **Estimated m/z** | **Error[ppm]** | **U.S.** | **C** | **H** | **O** | **S** |
| 267.1055 | -1.6 | 6.5 | 14 | 19 | 3 | 1 |

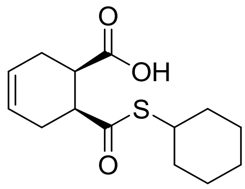

(2f)

**Figure S54.** $^{1}$H nmr

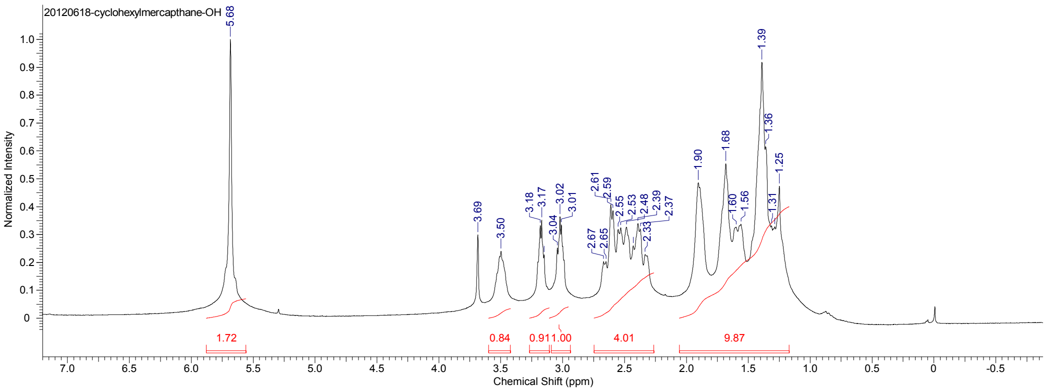

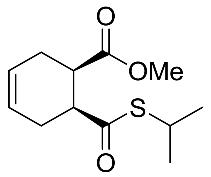


(2g)

**Figure S55.** $^{1}H$ nmr

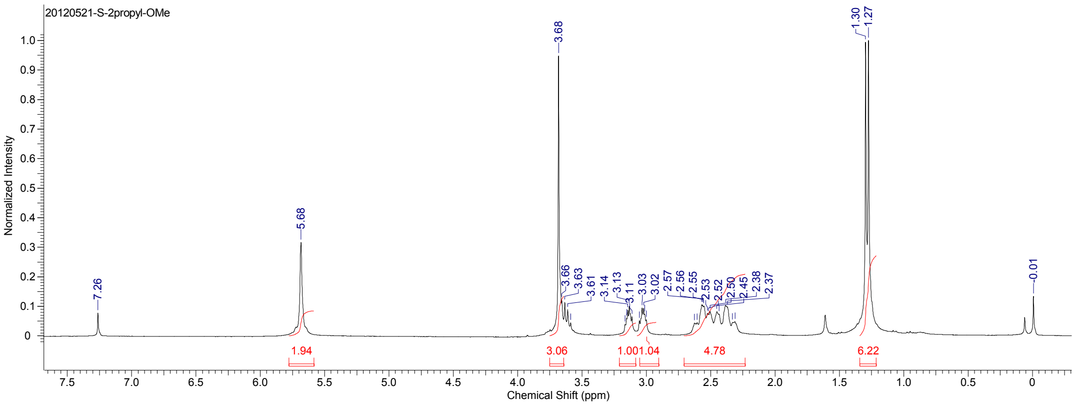


**Figure S56.** $^{13}C$ nmr

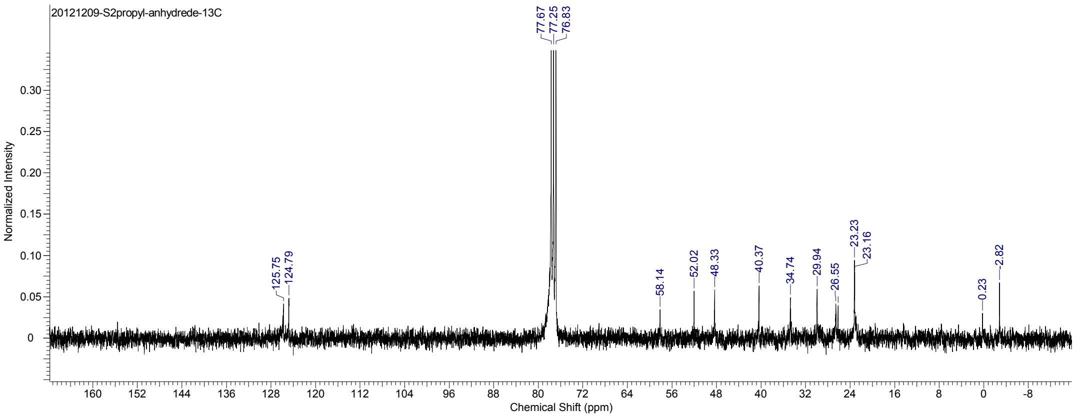

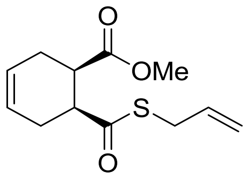


(2h)

**Figure S57.** $^1$H nmr

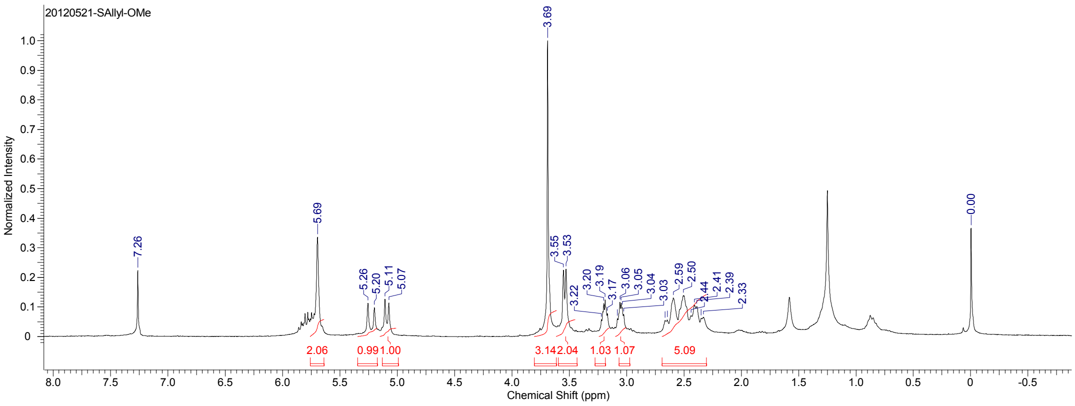


**Figure S58.** $^{13}$C nmr

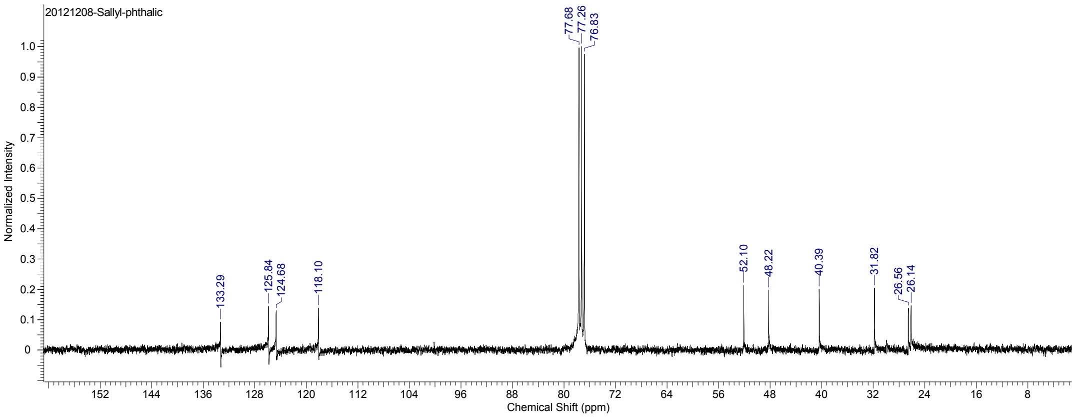

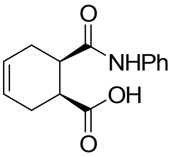


(2i)

**Figure S59.** $^{1}$H nmr

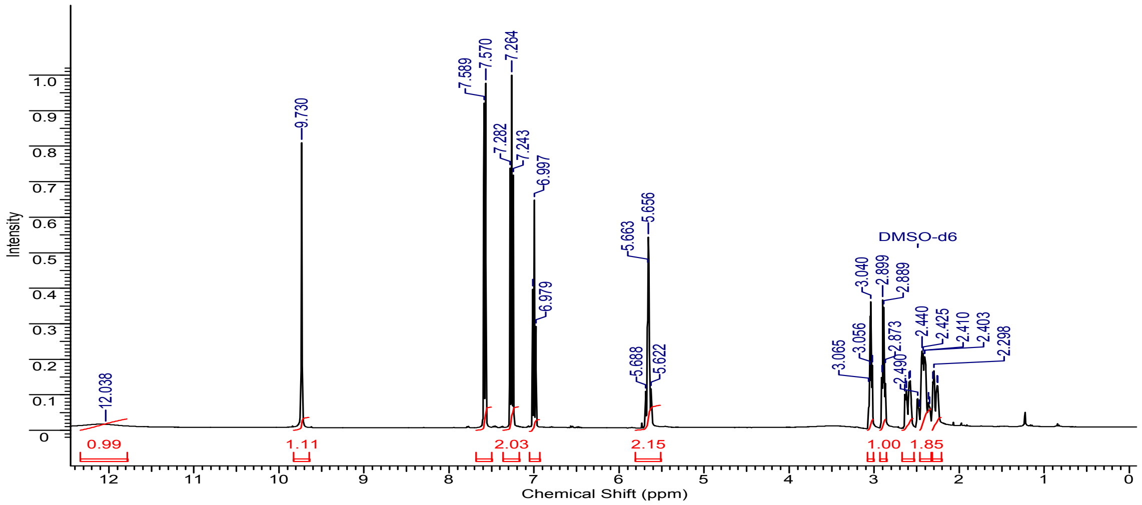


**Figure S60.** $^{13}$C nmr

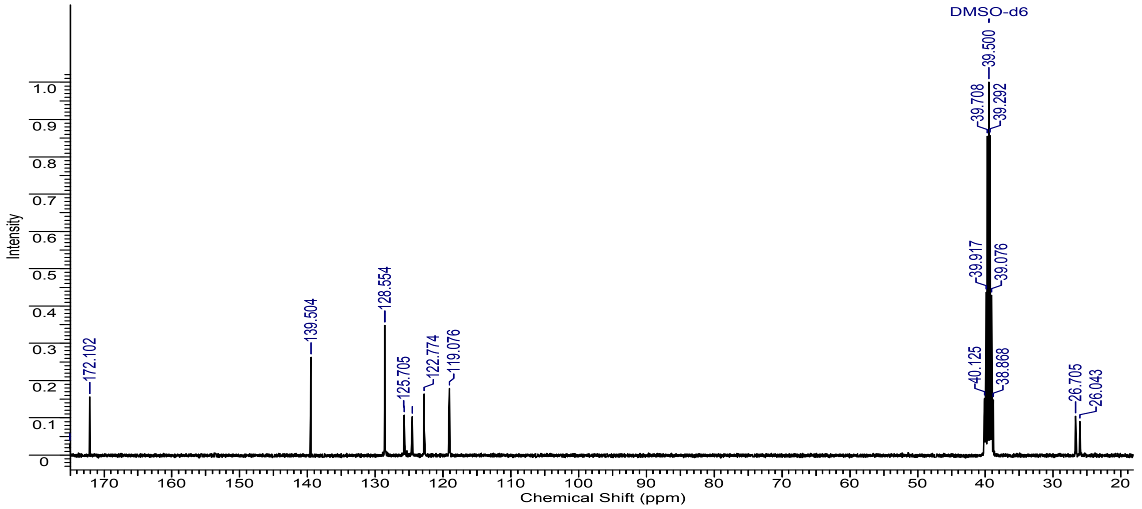


**Figure S61.** Mass

Inlet : Direct
RT : 2.71 min
Ion Mode : FAB+
Scan#: 29
Elements : C 14/0, H 16/0, O 3/0, N 1/0
Mass Tolerance : 20ppm, 50mmu if m/z > 2500
Unsaturation (U.S.) : 0.0 - 100.0

| Observed m/z | Int% | | | | | |
|---|---|---|---|---|---|---|
| 246.1111 | 94.7 | | | | | |
| **Estimated m/z** | **Error[ppm]** | **U.S.** | **C** | **H** | **O** | **N** |
| 246.1130 | -7.7 | 7.5 | 14 | 16 | 3 | 1 |

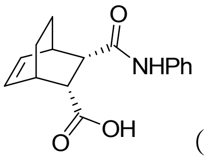


(2j)

**Figure S62.** $^{1}$H nmr

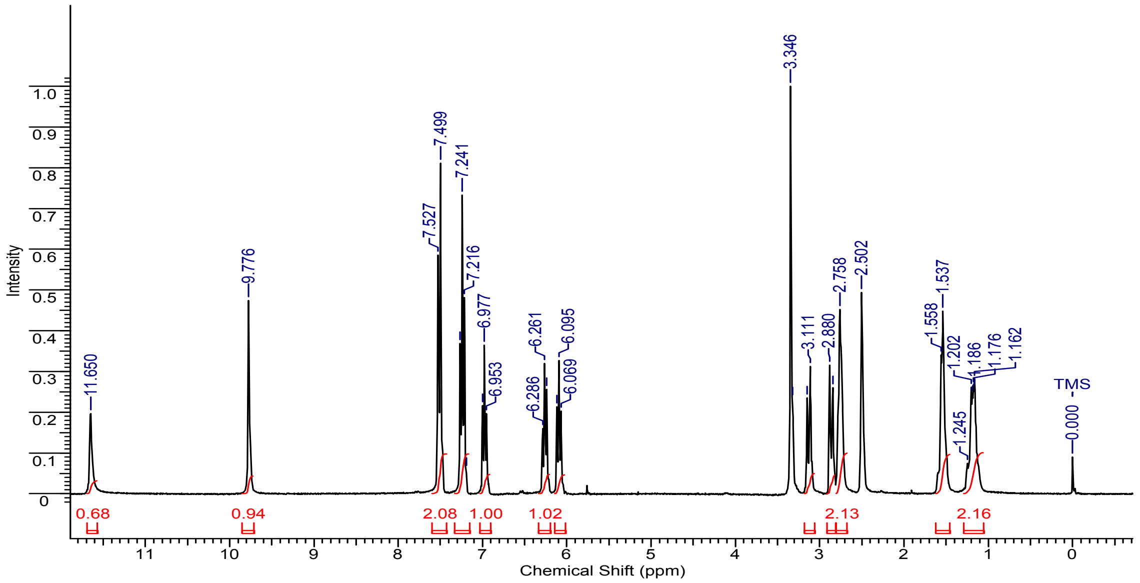


**Figure S63.** $^{13}$C nmr

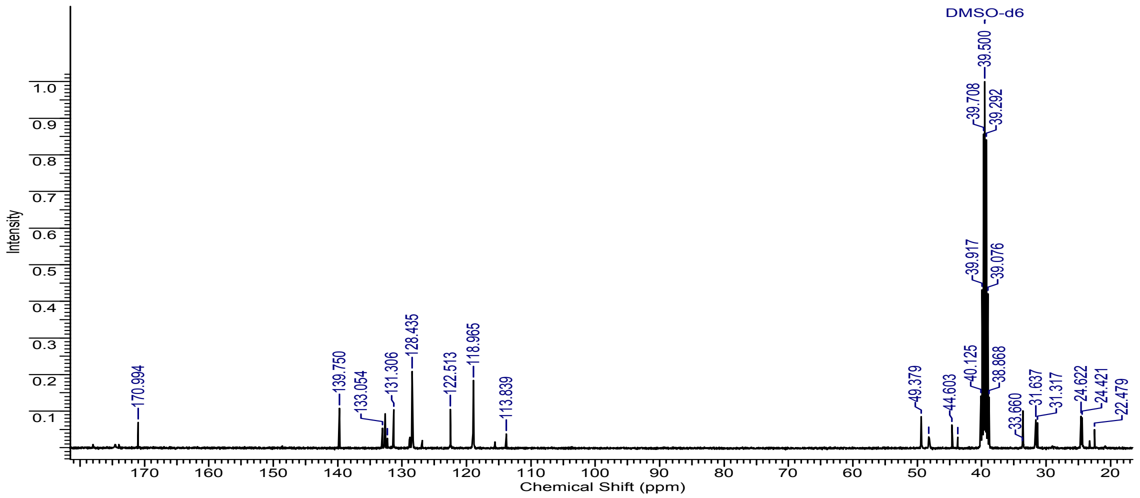


**Figure S64.** Mass

Inlet : Direct Ion Mode : FAB+
RT : 2.03 min Scan#: 22
Elements : C 16/0, H 18/0, O 5/0, N 3/0
Mass Tolerance : 5ppm, 10mmu if m/z > 2000
Unsaturation (U.S.) : 0.0 - 100.0

| Observed m/z | Int% | | | | | |
|---|---|---|---|---|---|---|
| 272.1276 | 31.6 | | | | | |
| Estimated m/z | Error[ppm] | U.S. | C | H | O | N |
| 272.1287 | -3.8 | 8.5 | 16 | 18 | 3 | 1 |

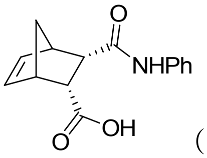


(2k)

**Figure S65.** [1]H nmr

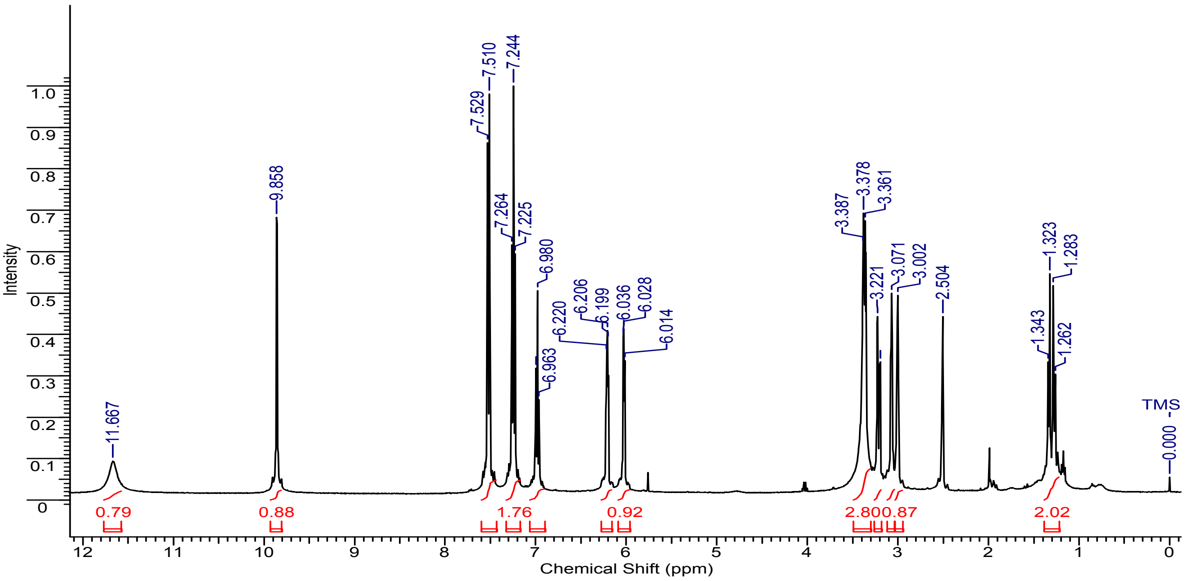


**Figure S66.** [13]C nmr

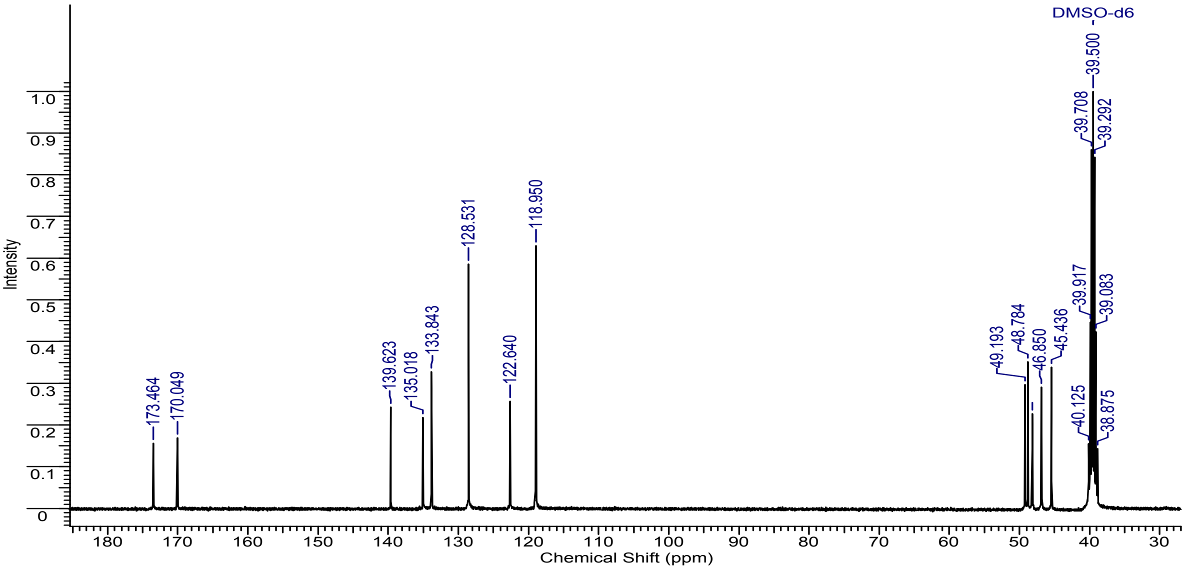


**Figure S67.** Mass

```
Inlet : Direct                              Ion Mode : FAB+
RT : 2.03 min                               Scan#: 22
Elements : C 15/0, H 16/0, O 3/0, N 1/0
Mass Tolerance        : 20ppm, 50mmu if m/z > 2500
Unsaturation (U.S.) : 0.0 - 100.0

Observed m/z    Int%
  258.1120       25.9
Estimated m/z  Error[ppm]  U.S.    C     H     O     N
  258.1130       -3.8       8.5    15    16    3     1
```

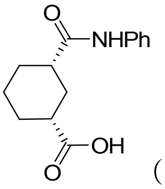


(21)

**Figure S68.** $^{1}$H nmr

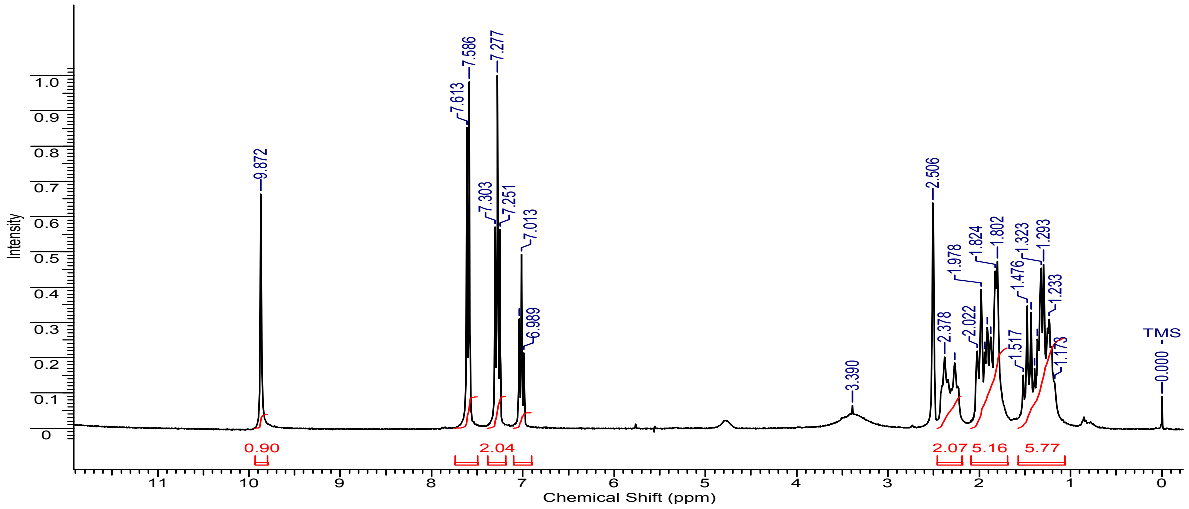


**Figure S69.** $^{13}$C nmr

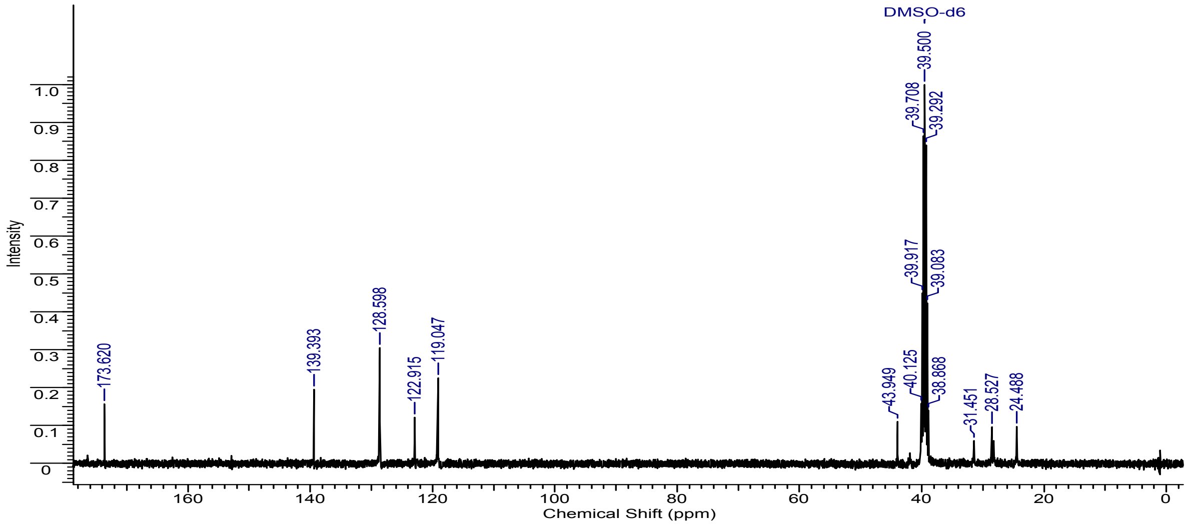


**Figure S70.** Mass

Inlet : Direct    Ion Mode : FAB+
RT : 2.13 min    Scan#: 23
Elements : C 14/0, H 18/0, O 3/0, N 1/0
Mass Tolerance : 5ppm, 10mmu if m/z > 2000
Unsaturation (U.S.) : 0.0 - 100.0

| Observed m/z | Int% |
|---|---|
| 248.1283 | 90.2 |

| Estimated m/z | Error[ppm] | U.S. | C | H | O | N |
|---|---|---|---|---|---|---|
| 248.1287 | -1.5 | 6.5 | 14 | 18 | 3 | 1 |

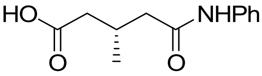


(2m)

**Figure S71.** $^{1}$H nmr

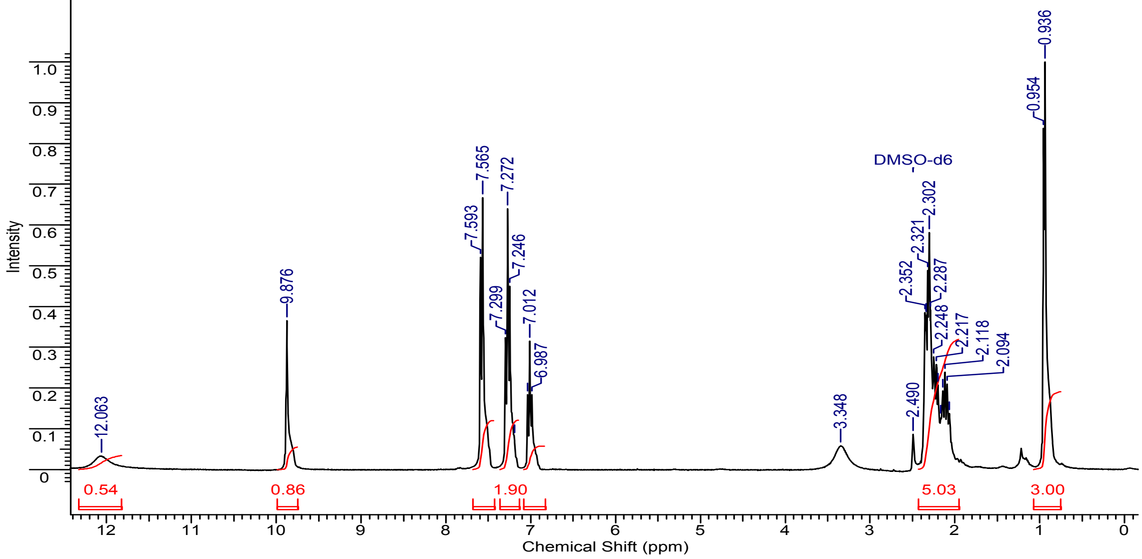


**Figure S72.** $^{13}$C nmr

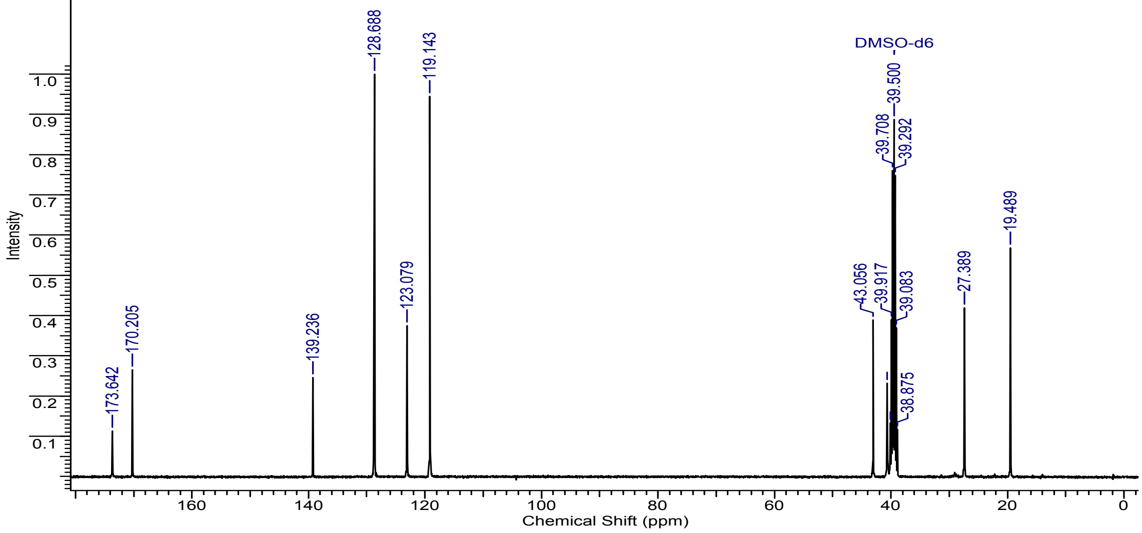


**Figure S73.** Mass

Inlet : Direct  Ion Mode : FAB+
RT : 3.00 min  Scan#: 32
Elements : C 12/0, H 16/0, O 3/0, N 1/0
Mass Tolerance : 20ppm, 50mmu if m/z > 2500
Unsaturation (U.S.) : 0.0 - 100.0

| Observed m/z | Int% | | | | | |
|---|---|---|---|---|---|---|
| 222.1128 | 45.6 | | | | | |
| Estimated m/z | Error[ppm] | U.S. | C | H | O | N |
| 222.1130 | -0.9 | 5.5 | 12 | 16 | 3 | 1 |

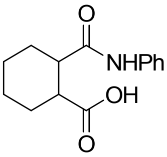


(2n)

**Figure S74.** $^{1}$H nmr

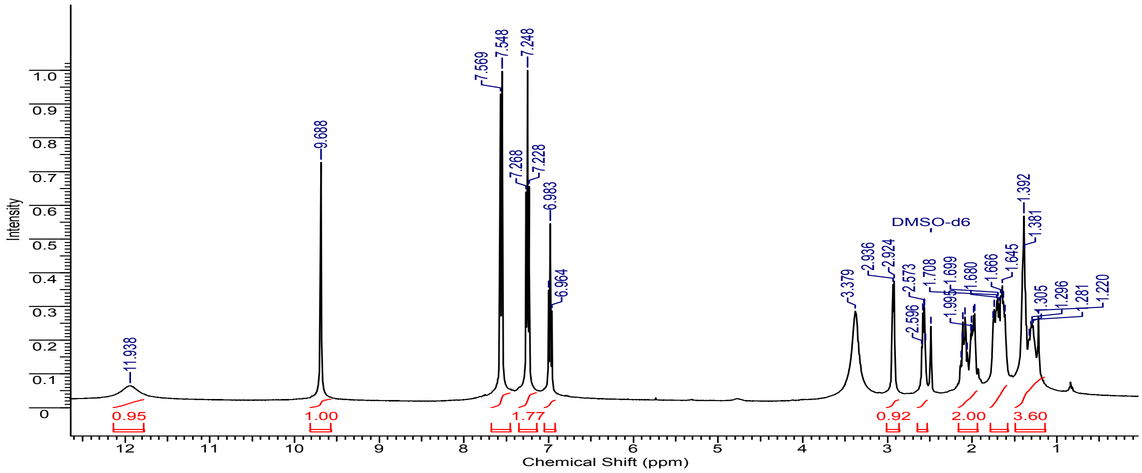


**Figure S75.** $^{13}$C nmr

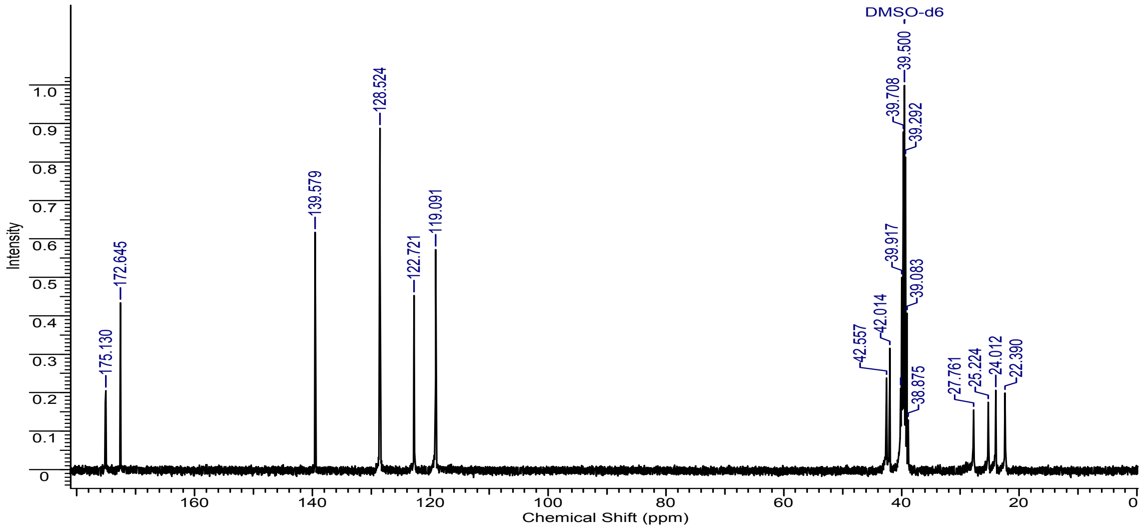


**Figure S76.** Mass

```
Inlet : Direct                                  Ion Mode : FAB+
RT : 2.32 min                                   Scan#: 25
Elements : C 48/0, H 56/0, O 5/0, N 3/0
Mass Tolerance        : 5ppm, 10mmu if m/z > 2000
Unsaturation (U.S.)  : 0.0 - 100.0

Observed m/z   Int%
  248.1279     100.0
Estimated m/z  Error[ppm]   U.S.    C     H     O     N
  248.1287       -3.0        6.5   14    18     3     1
```

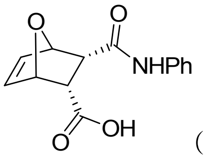


(2o)

**Figure S77.** [1]H nmr

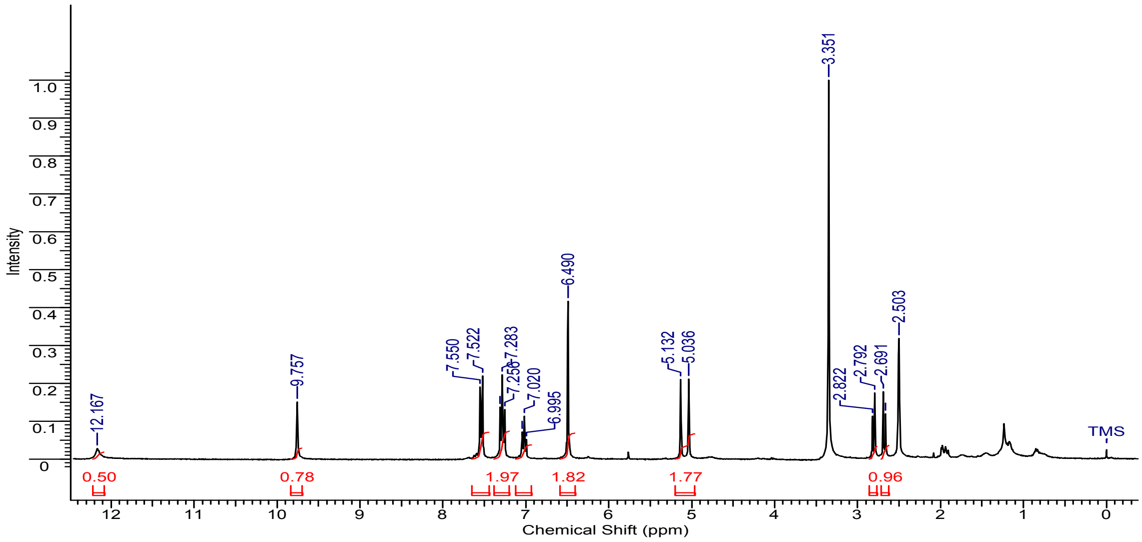


**Figure S78.** [13]C nmr

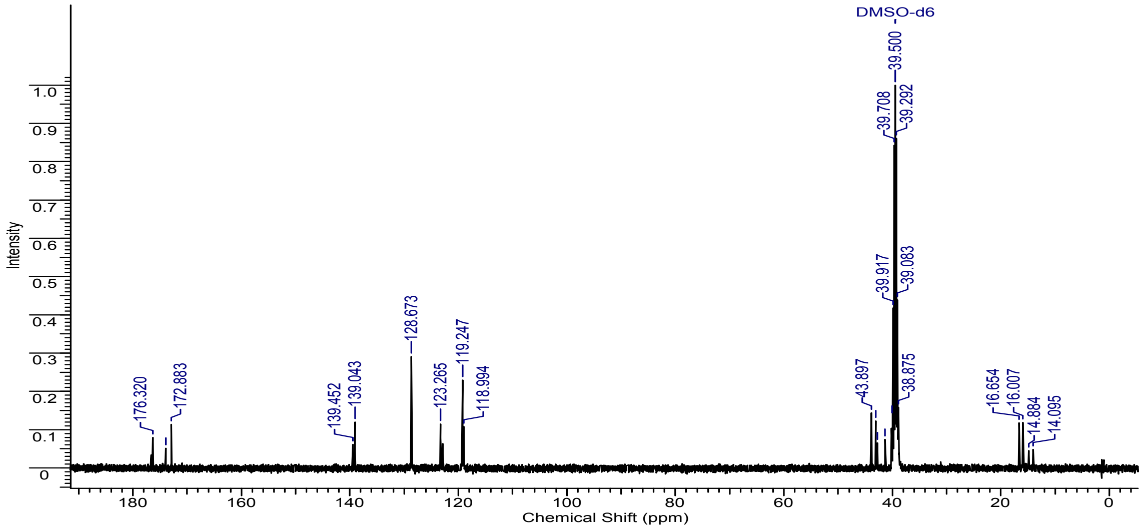


**Figure S79.** Mass

Inlet : Direct                                    Ion Mode : FAB+
RT : 2.67 min                                     Scan#: 33
Elements : C 14/0, H 14/0, O 4/0, N 1/0
Mass Tolerance : 20ppm, 50mmu if m/z > 2500
Unsaturation (U.S.) : 0.0 - 100.0

| Observed m/z | Int% | | | | | |
|---|---|---|---|---|---|---|
| 260.0945 | 100.0 | | | | | |
| Estimated m/z | Error[ppm] | U.S. | C | H | O | N |
| 260.0923 | +8.3 | 8.5 | 14 | 14 | 4 | 1 |

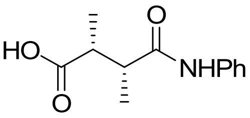


(2p)

**Figure S80.** $^{1}$H nmr

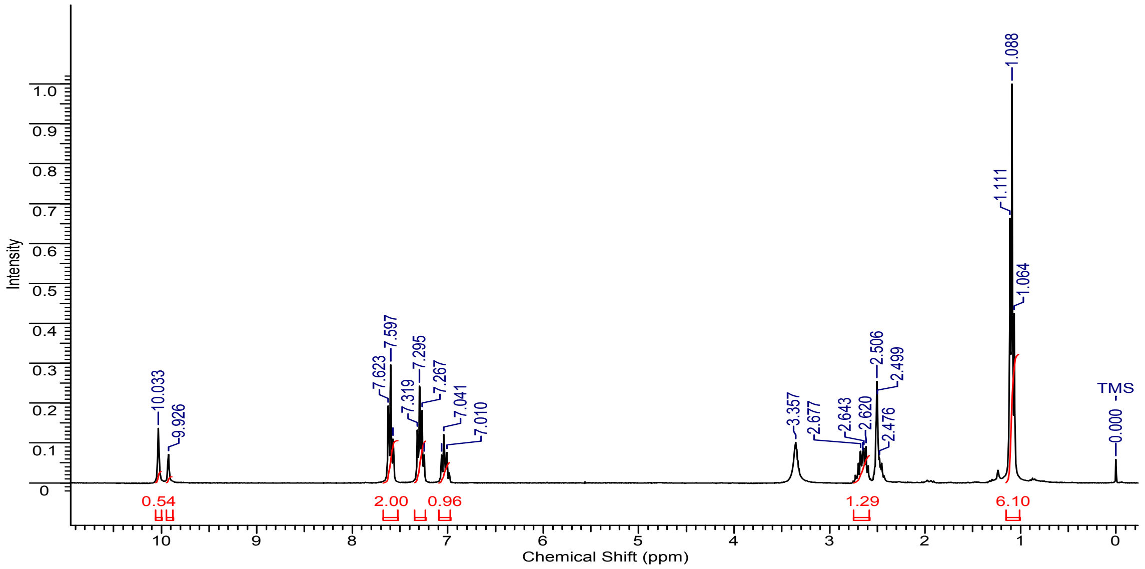


**Figure S81.** $^{13}$C nmr

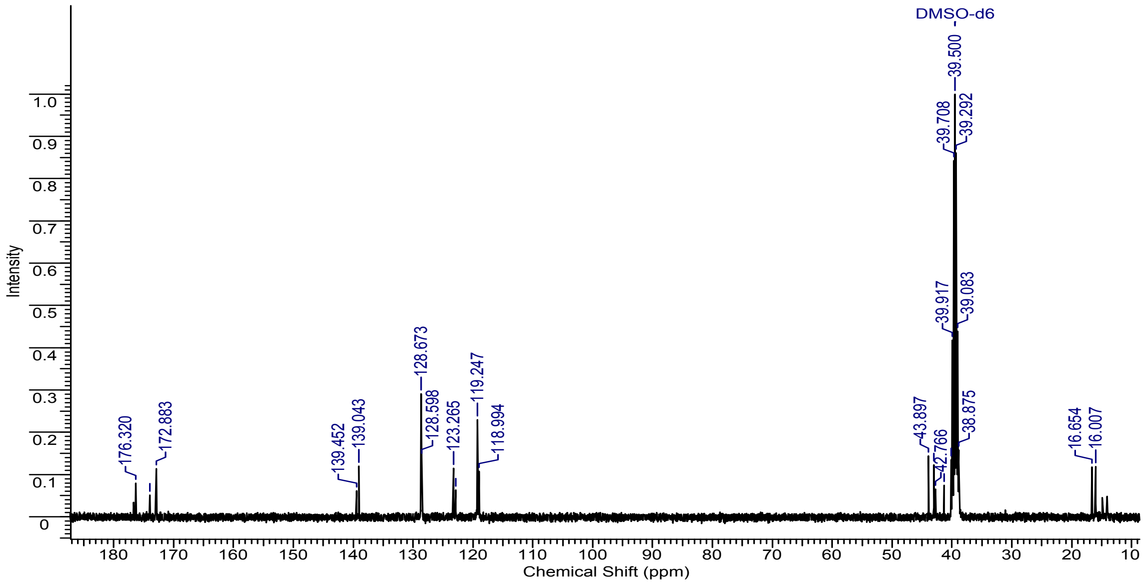


**Figure S82.** Mass

```
Inlet : Direct                          Ion Mode : FAB+
RT : 2.32 min                           Scan#: 25
Elements : C 12/0, H 16/0, O 3/0, N 1/0
Mass Tolerance          : 20ppm, 50mmu if m/z > 2500
Unsaturation (U.S.) : 0.0 - 100.0

Observed m/z   Int%
  222.1131      62.1
Estimated m/z  Error[ppm]  U.S.    C     H     O     N
  222.1130       +0.4       5.5    12    16    3     1
```

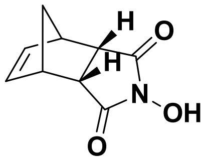

(3a)

**Figure S83.** $^{1}$H nmr

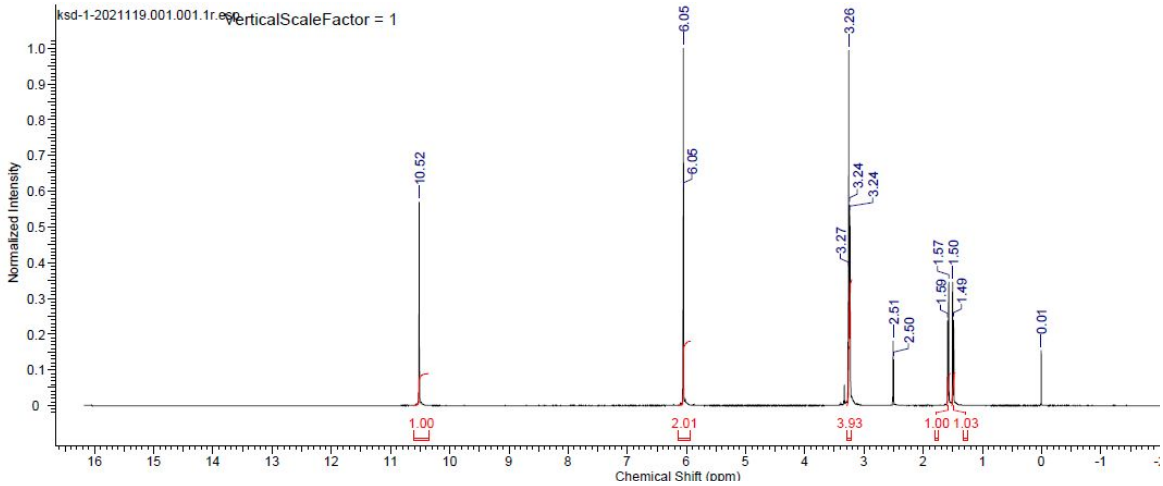


**Figure S84.** $^{13}$C nmr

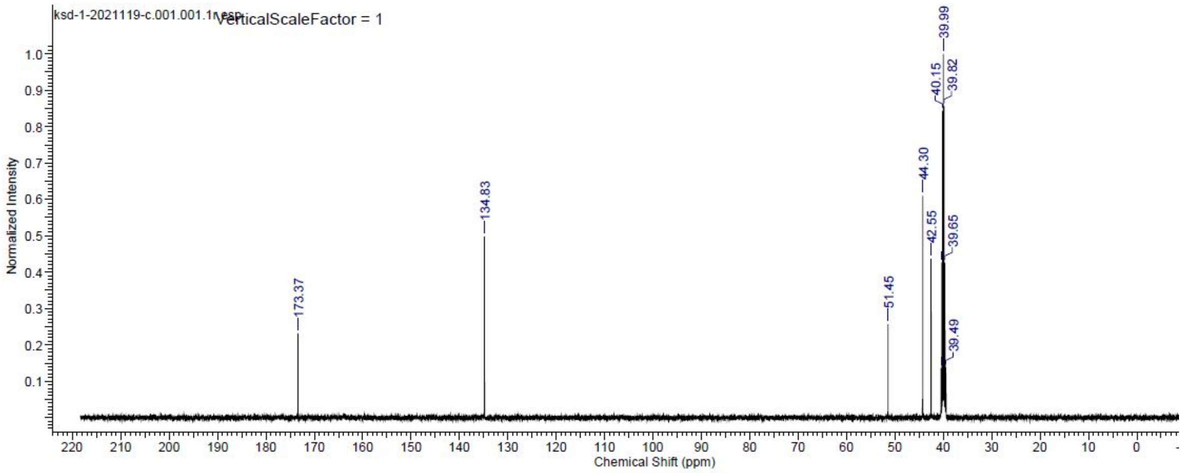


**Figure S85.** Mass

Note : Glycerol + DMSO

Inlet : Direct Ion Mode : FAB+

Spectrum Type : Normal Ion [MF–Linear]

RT : 0.00 min Scan# : (1,3) Temp : 3276.7 deg.C

BP : m/z 180 Int. : 238.17 (2497397)

Output m/z range : 10 to 200 Cut Level : 0.00 %

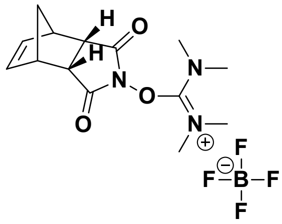

(3b)

**Figure S86.** $^{1}$H nmr

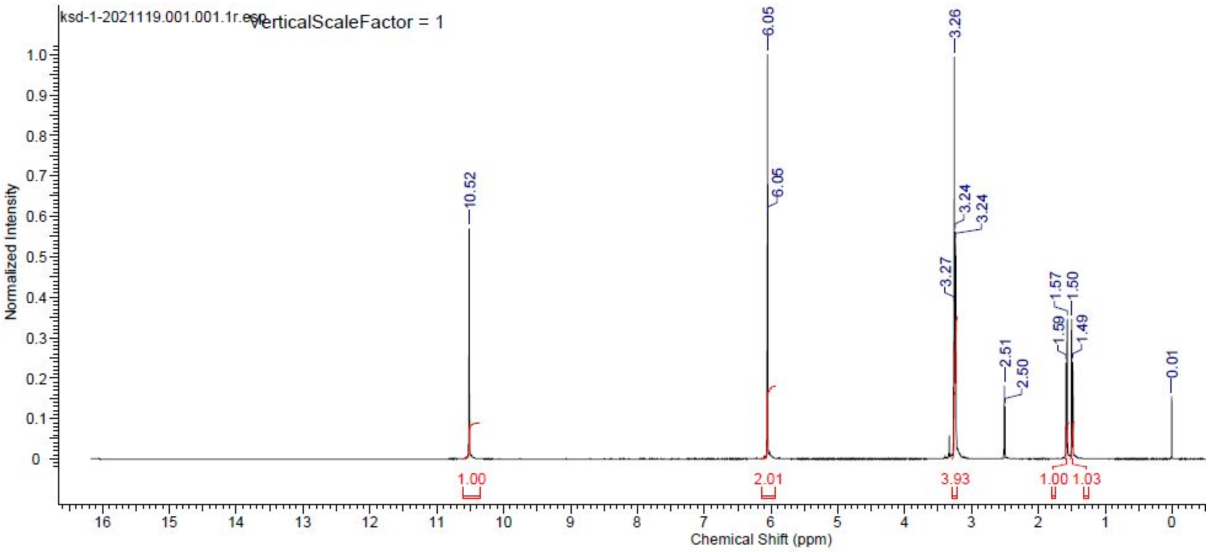


**Figure S87.** $^{13}$C nmr

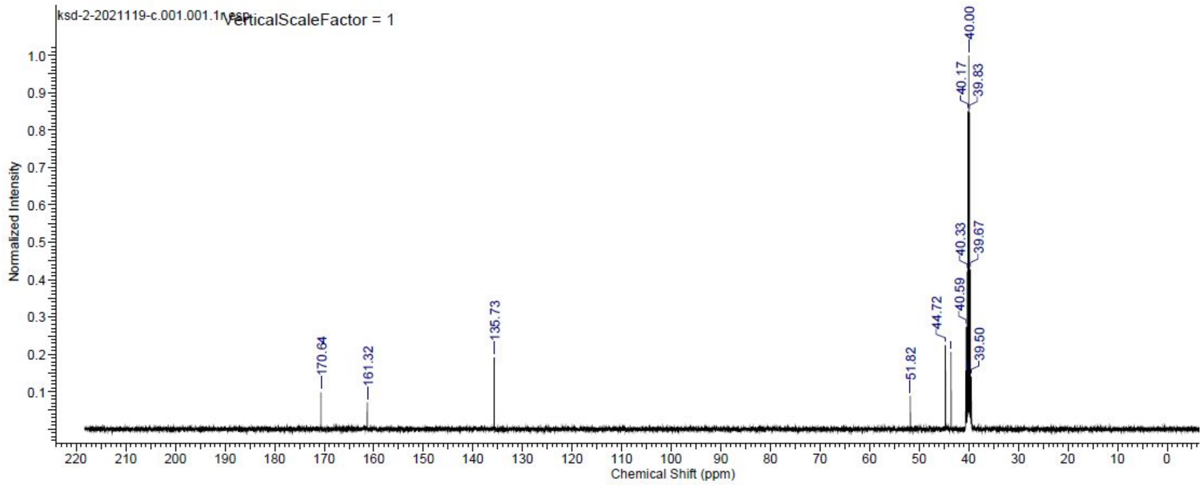


**Figure S88.** Mass

Note : Glycerol + DMSO

Inlet : Direct Ion Mode : FAB+

Spectrum Type : Normal Ion [MF-Linear]

RT : 0.00 min Scan# : (1,5) Temp : 3276.7 deg.C

BP : m/z 278 Int. : 207.43 (2175097)

Output m/z range : 10 to 700 Cut Level : 0.00 %

## 4. HPLC Spectra

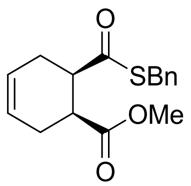


**Racemic**

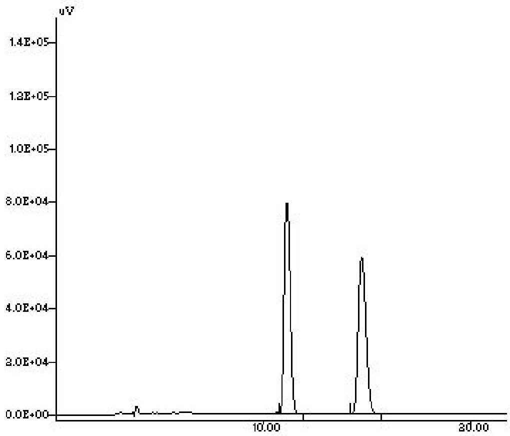


| # | Name | RT | Area[uV.Sec] | Quantity |
|---|---|---|---|---|
| 1 | | 11.100 | 1295062.850 | 0.000 |
| 2 | | 14.708 | 1295801.500 | 0.000 |

Total Area of Peak = 2590864.350 [uV.Sec]

**Table 1.1**

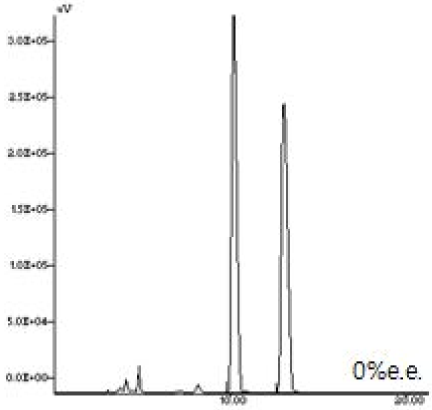


| # | Name | RT | Area[uV.Sec] | Quantity |
|---|---|---|---|---|
| 1 | | 10.167 | 5322127.000 | 0.000 |
| 2 | | 13.008 | 5282768.750 | 0.000 |

Total Area of Peak = 10604895.750 [uV.Sec]

**Table 1.2**

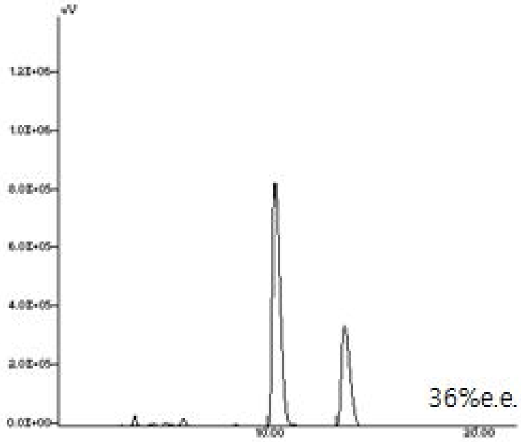


| # | Name | RT | Area[uV.Sec] | Quantity |
|---|---|---|---|---|
| 1 | | 10.350 | 17190996.500 | 0.000 |
| 2 | | 13.675 | 8175217.000 | 0.000 |

Total Area of Peak = 25366213.500 [uV.Sec]

**Table 1.3**

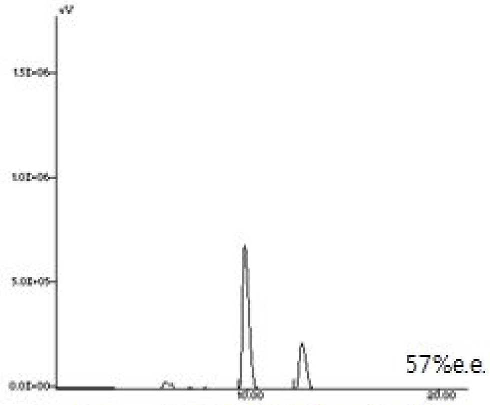


| # | Name | RT | Area[uV.Sec] | Quantity |
|---|---|---|---|---|
| 1 | | 9.833 | 12570535.000 | 0.000 |
| 2 | | 12.825 | 4468304.250 | 0.000 |

Total Area of Peak = 17038839.250 [uV.Sec]

**Table 1.4**

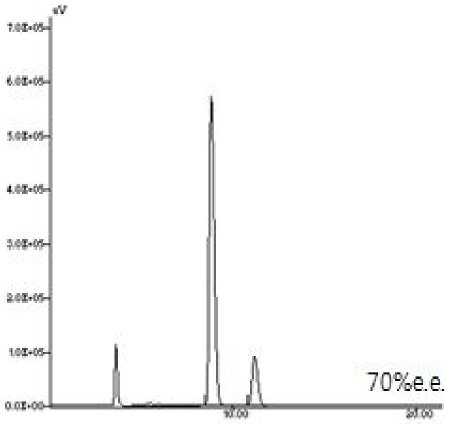


| # | Name | RT | Area[uV.Sec] | Quantity |
|---|---|---|---|---|
| 1 | | 8.742 | 8864879.148 | 0.000 |
| 2 | | 11.100 | 1569357.271 | 0.000 |

Total Area of Peak = 10434236.419 [uV.Sec]

**Table 1.5**

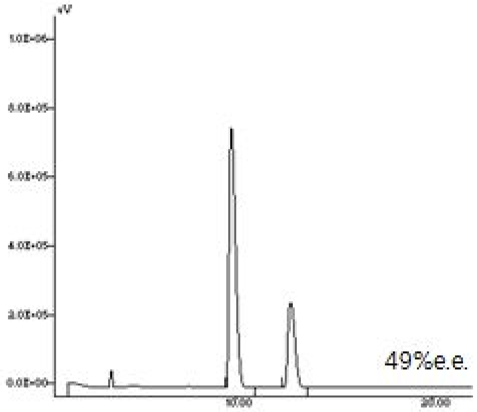


| # | Name | RT | Area[uV.Sec] | Quantity |
|---|---|---|---|---|
| 1 | | 9.733 | 13203032.500 | 0.000 |
| 2 | | 12.742 | 4529856.500 | 0.000 |

Total Area of Peak = 17732889.000 [uV.Sec]

**Table 1.6**

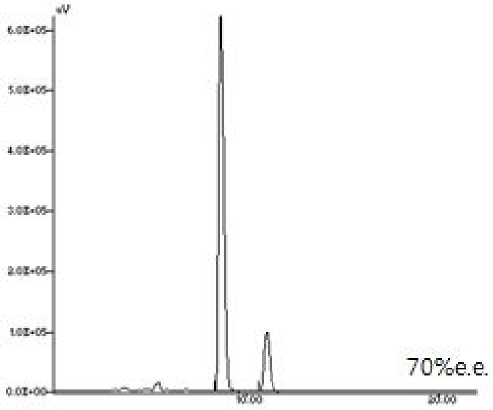


| # | Name | RT | Area[uV.Sec] | Quantity |
|---|---|---|---|---|
| 1 | | 8.642 | 9201096.500 | 0.000 |
| 2 | | 11.025 | 1646287.500 | 0.000 |

Total Area of Peak = 10847384.000 [uV.Sec]

**Table 1.7**

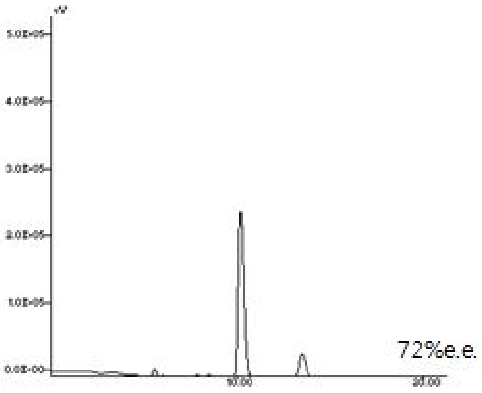


| # | Name | RT | Area[uV.Sec] | Quantity |
|---|---|---|---|---|
| 1 | | 10.133 | 4057692.327 | 0.000 |
| 2 | | 13.450 | 655471.500 | 0.000 |

Total Area of Peak = 4713163.827 [uV.Sec]

**Table 1.8**

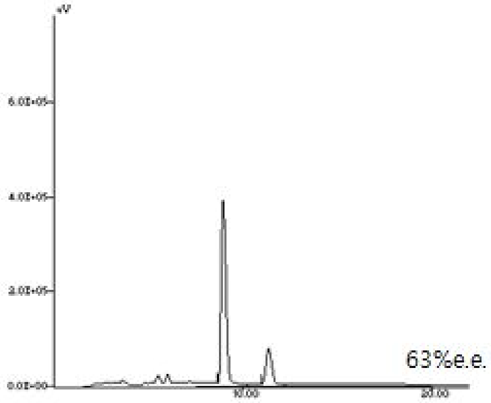


| # | Name | RT | Area[uV.Sec] | Quantity |
|---|---|---|---|---|
| 1 | | 8.908 | 6071789.126 | 0.000 |
| 2 | | 11.317 | 1663280.822 | 0.000 |

Total Area of Peak = 7735069.947 [uV.Sec]

**Table 1.9**

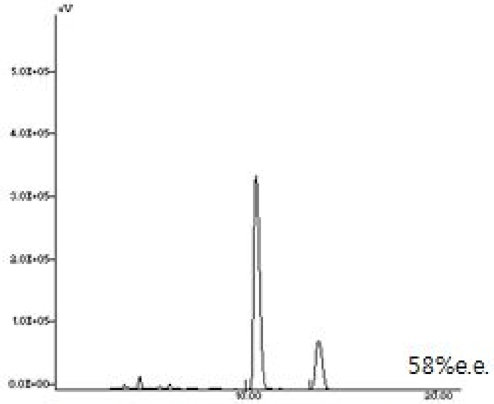


| # | Name | RT | Area[uV.Sec] | Quantity |
|---|---|---|---|---|
| 1 | | 10.500 | 5752142.250 | 0.000 |
| 2 | | 13.800 | 1547907.500 | 0.000 |

Total Area of Peak = 7300049.750 [uV.Sec]

**Table 1.10**

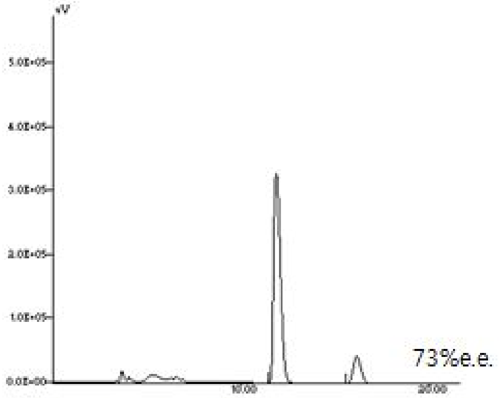


| # | Name | RT | Area[uV.Sec] | Quantity |
|---|---|---|---|---|
| 1 | | 10.825 | 6220564.750 | 0.000 |
| 2 | | 14.742 | 957890.500 | 0.000 |

Total Area of Peak = 7178455.250 [uV.Sec]

**Table 1.11**

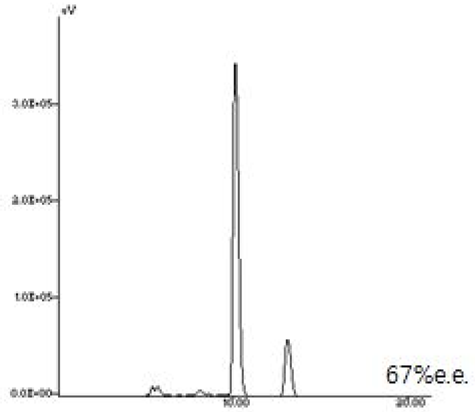


| # | Name | RT | Area[uV.Sec] | Quantity |
|---|---|---|---|---|
| 1 | | 10.083 | 5940954.936 | 0.000 |
| 2 | | 13.083 | 1155710.500 | 0.000 |

Total Area of Peak = 7096665.436 [uV.Sec]

**Table 1.12**

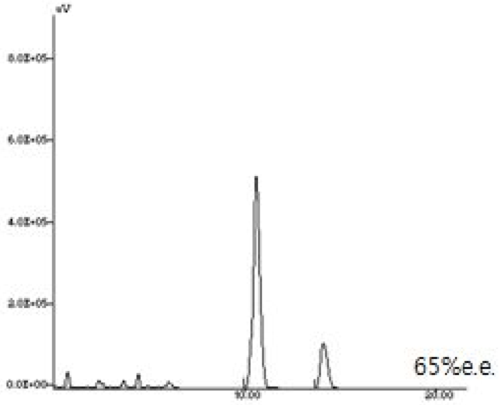


| # | Name | RT | Area[uV.Sec] | Quantity |
|---|---|---|---|---|
| 1 | | 10.558 | 11144735.467 | 0.000 |
| 2 | | 14.083 | 2420353.000 | 0.000 |

Total Area of Peak = 13565088.467 [uV.Sec]

**Table 1.13**

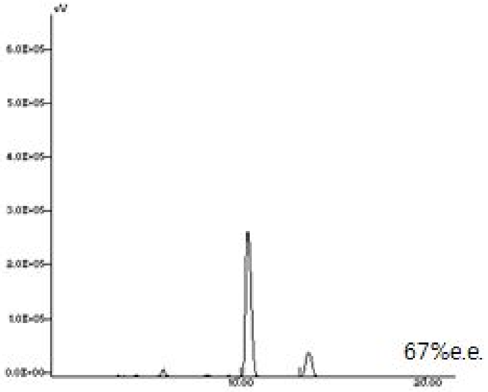


| # | Name | RT | Area[uV.Sec] | Quantity |
|---|---|---|---|---|
| 1 | | 10.492 | 4322908.702 | 0.000 |
| 2 | | 13.742 | 860819.682 | 0.000 |

Total Area of Peak = 5183728.384 [uV.Sec]

**Table 1.14**

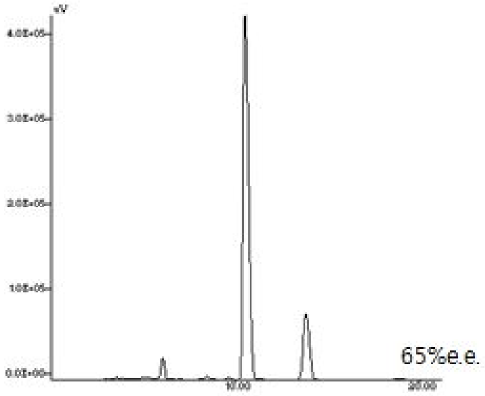


| # | Name | RT | Area[uV.Sec] | Quantity |
|---|---|---|---|---|
| 1 | | 10.492 | 7309408.500 | 0.000 |
| 2 | | 13.783 | 1547320.500 | 0.000 |

Total Area of Peak = 8856729.000 [uV.Sec]

**Table 1.15**

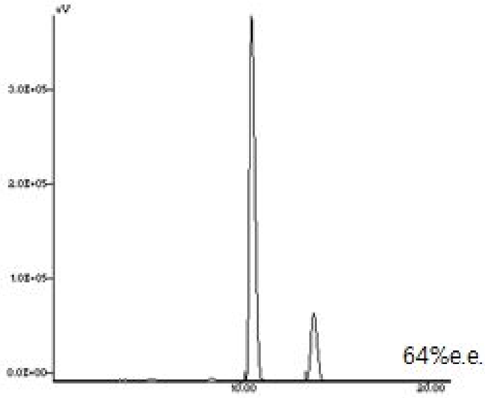


| # | Name | RT | Area[uV.Sec] | Quantity |
|---|---|---|---|---|
| 1 | | 10.533 | 6554974.500 | 0.000 |
| 2 | | 13.850 | 1431624.000 | 0.000 |

Total Area of Peak = 7986598.500 [uV.Sec]

**Table 1.16**

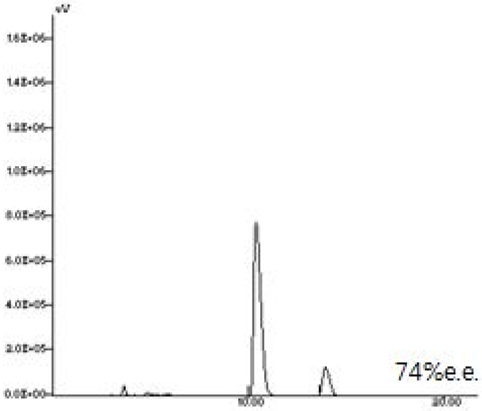


| # | Name | RT | Area[uV.Sec] | Quantity |
|---|---|---|---|---|
| 1 | | 10.375 | 16737825.750 | 0.000 |
| 2 | | 13.917 | 2492398.500 | 0.000 |

Total Area of Peak = 19230224.250 [uV.Sec]

**Table 2.1**

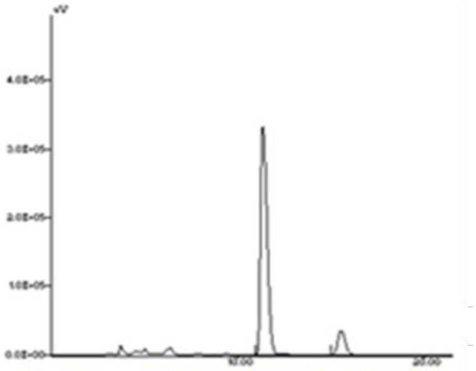

| # | Name | RT | Area[uV.Sec] | Quantity |
|---|---|---|---|---|
| 1 | | 11.333 | 6788785.000 | 0.000 |
| 2 | | 15.533 | 832457.500 | 0.000 |

Total Area of Peak = 7621242.500 [uV.Sec]

**Table 2.2**

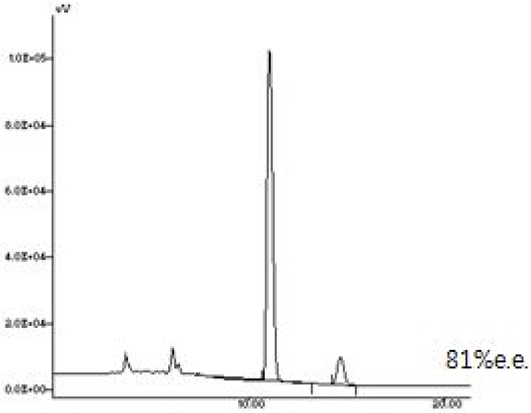


| # | Name | RT | Area[uV.Sec] | Quantity |
|---|---|---|---|---|
| 1 | | 11.025 | 1603647.823 | 0.000 |
| 2 | | 14.675 | 168499.013 | 0.000 |

Total Area of Peak = 1772146.836 [uV.Sec]

**Table 2.3**

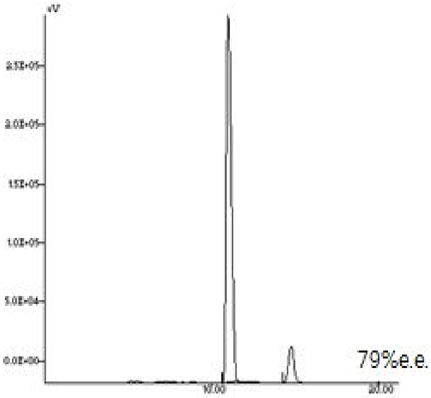


| # | Name | RT | Area[uV.Sec] | Quantity |
|---|---|---|---|---|
| 1 | | 10.950 | 5654154.000 | 0.000 |
| 2 | | 14.725 | 674559.500 | 0.000 |

Total Area of Peak = 6328713.500 [uV.Sec]

**Table 2.4**

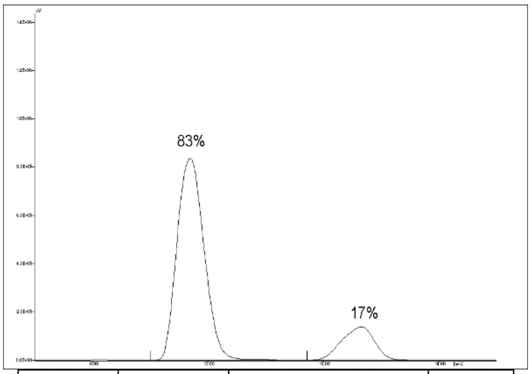


| Name | RT | Area [uV.Sec] | Area [%] |
|---|---|---|---|
| 1 | 9.692 | 26738159.359 | 83 |
| 2 | 12.650 | 5389003.000 | 17 |

**Table 2.5**

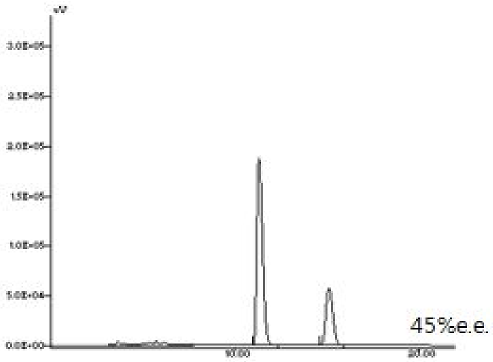


| # | Name | RT | Area[uV.Sec] | Quantity |
|---|---|---|---|---|
| 1 | | 11.275 | 3364237.384 | 0.000 |
| 2 | | 15.058 | 1268801.500 | 0.000 |

Total Area of Peak = 4633038.884 [uV.Sec]

**Table 2.6**

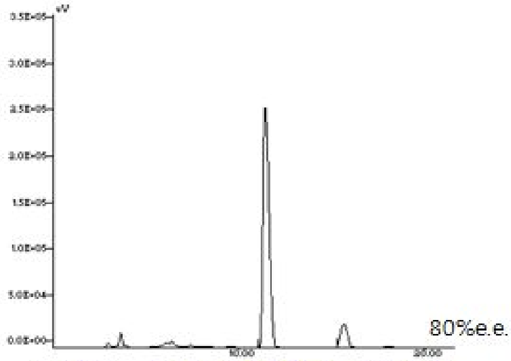


| # | Name | RT | Area[uV.Sec] | Quantity |
|---|---|---|---|---|
| 1 | | 11.375 | 4889235.000 | 0.000 |
| 2 | | 15.592 | 536824.500 | 0.000 |

Total Area of Peak = 5426059.500 [uV.Sec]

**Table 2.7**

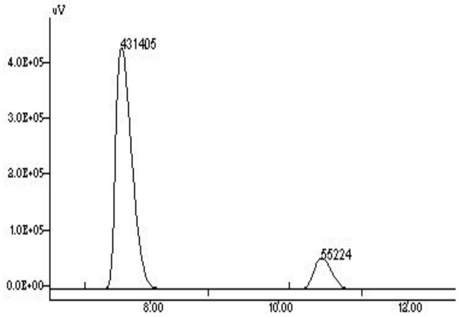


| # | Name | RT | Area[uV.Sec] | Quantity |
|---|---|---|---|---|
| 1 | | 7.550 | 7104428.750 | 0.000 |
| 2 | | 10.667 | 1044605.000 | 0.000 |

Total Area of Peak = 8149033.750 [uV.Sec]

**Table 2.8**

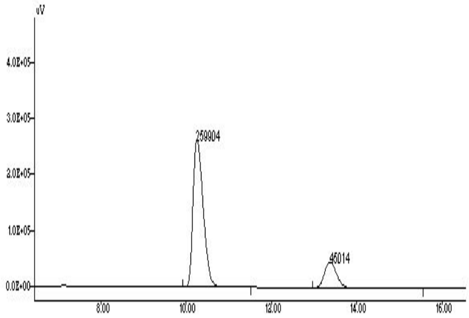

| # | Name | RT | Area[uV.Sec] | Quantity |
|---|---|---|---|---|
| 1 | | 10.250 | 4056959.000 | 0.000 |
| 2 | | 13.367 | 851050.286 | 0.000 |

Total Area of Peak = 4908009.286 [uV.Sec]

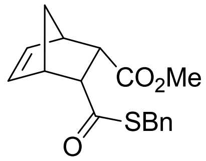


**(2b)**

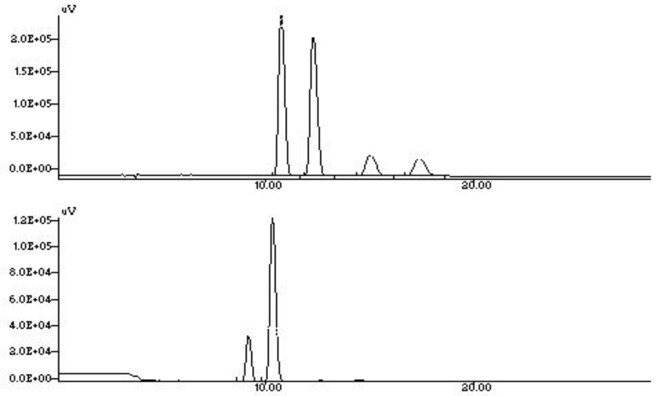

| # | Name | RT | Area[uV.Sec] | Quantity |
|---|---|---|---|---|
| 1 | | 9.125 | 475299.687 | 0.000 |
| 2 | | 10.283 | 1953041.939 | 0.000 |

Total Area of Peak = 2428341.626 [uV.Sec]

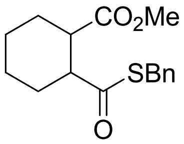


**(2c)**

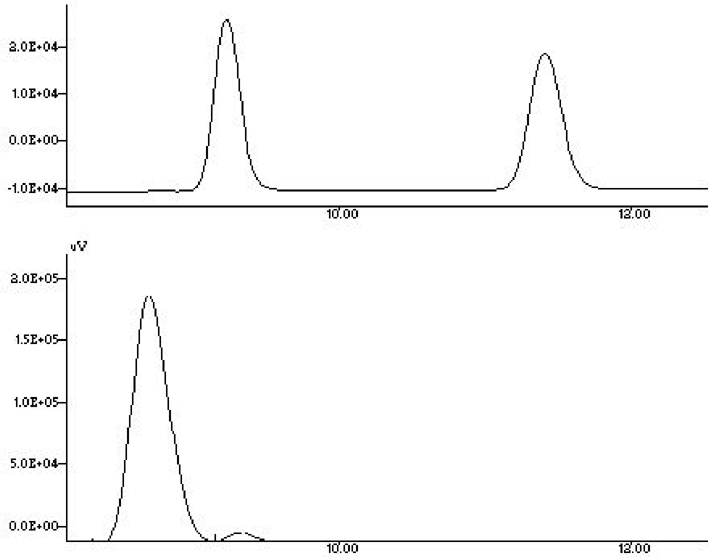

| # | Name | RT | Area[uV.Sec] | Quantity |
|---|---|---|---|---|
| 1 | | 8.533 | 2586122.500 | 0.000 |
| 2 | | 8.992 | 80536.000 | 0.000 |

Total Area of Peak = 2666658.500 [uV.Sec]

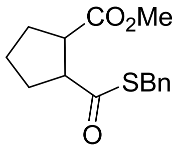


**(2d)**

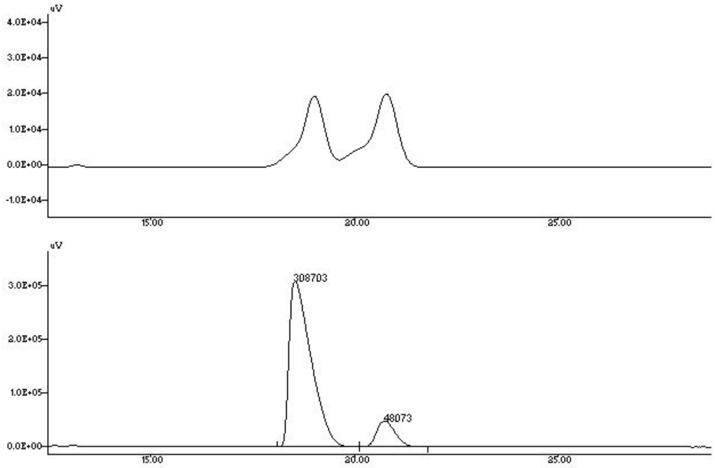


| # | Name | RT | Area[uV.Sec] | Quantity |
|---|---|---|---|---|
| 1 | | 18.533 | 10998383.000 | 0.000 |
| 2 | | 20.733 | 1446792.230 | 0.000 |

Total Area of Peak = 12445175.230 [uV.Sec]

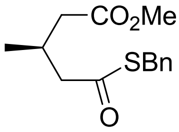


**(2e)**

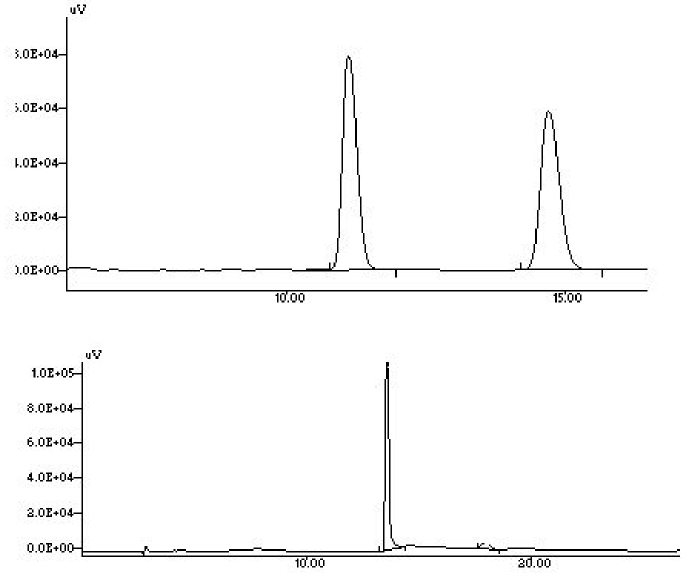


| # | Name | RT | Area[uV.Sec] | Quantity |
|---|---|---|---|---|
| 1 | | 13.542 | 908987.496 | 0.000 |
| 2 | | 17.925 | 45260.000 | 0.000 |

Total Area of Peak = 954247.496 [uV.Sec]

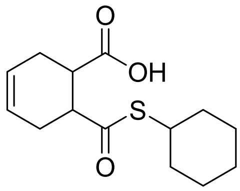


**(2f)**

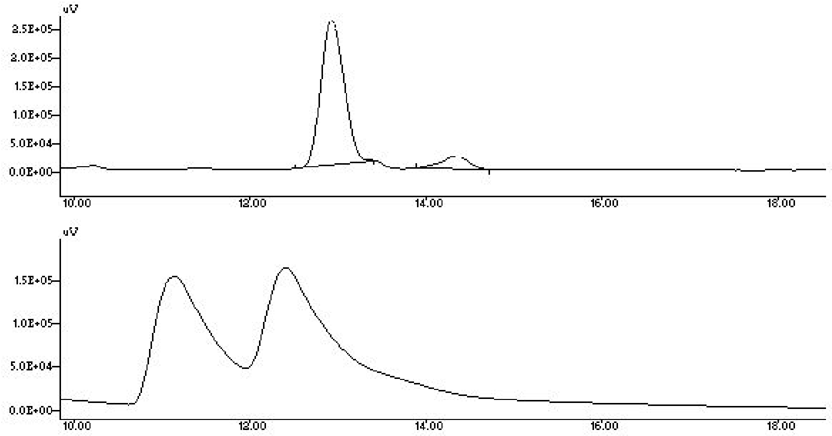


| # | Name | RT | Area[uV.Sec] | Quantity |
|---|---|---|---|---|
| 1 | | 12.925 | 4340729.455 | 0.000 |
| 2 | | 14.342 | 455723.500 | 0.000 |

Total Area of Peak = 4796452.955 [uV.Sec]

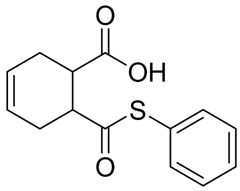


**(2g)**

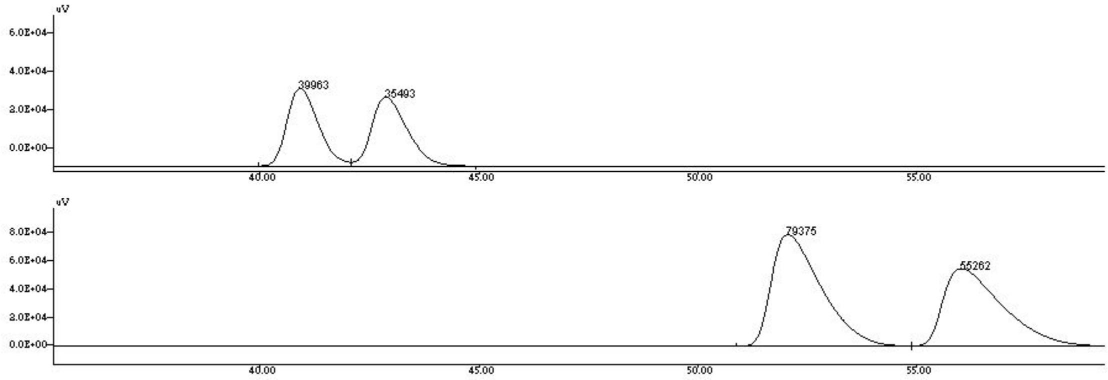


| # | Name | RT | Area[uV.Sec] | Quantity |
|---|---|---|---|---|
| 1 | | 52.050 | 6173120.470 | 0.000 |
| 2 | | 56.017 | 5100799.780 | 0.000 |

Total Area of Peak = 11273920.250 [uV.Sec]

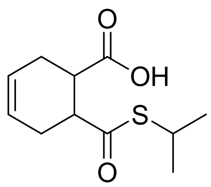

**(2h)**

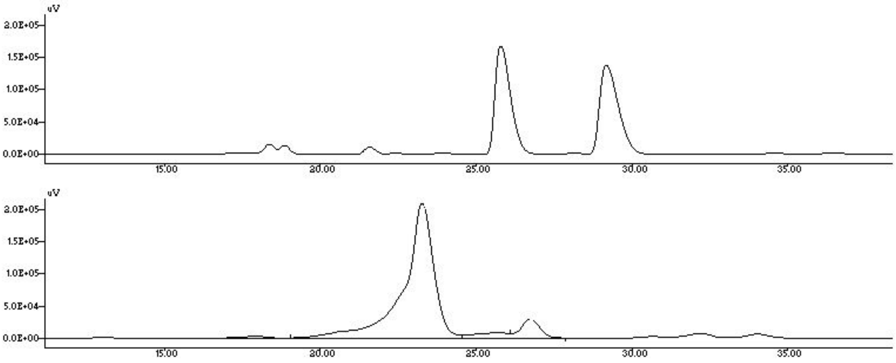


| # | Name | RT | Area[uV.Sec] | Quantity |
|---|---|---|---|---|
| 1 | | 23.258 | 13663267.856 | 0.000 |
| 2 | | 26.725 | 1176687.827 | 0.000 |

Total Area of Peak = 14839955.683 [uV.Sec]

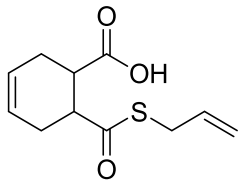

**(2i)**

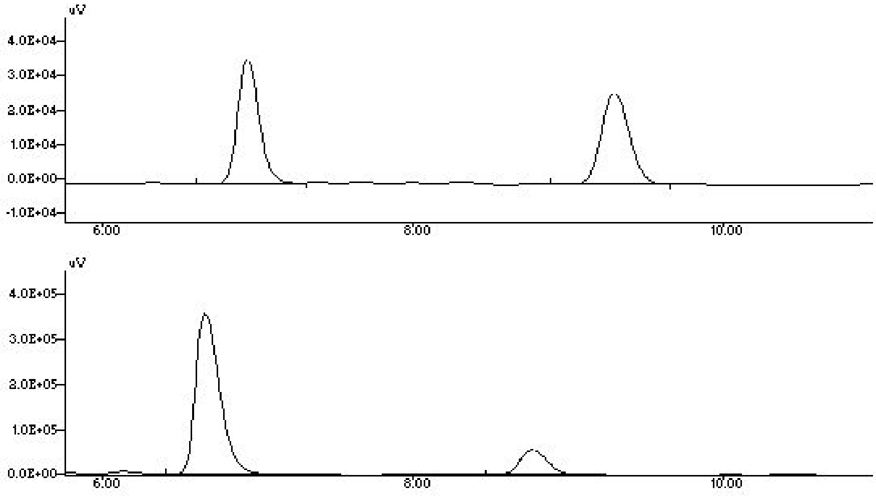


| # | Name | RT | Area[uV.Sec] | Quantity |
|---|---|---|---|---|
| 1 | | 6.658 | 3605684.618 | 0.000 |
| 2 | | 8.767 | 635419.233 | 0.000 |

Total Area of Peak = 4241103.850 [uV.Sec]

## 5. Copy of GC Chromatograms

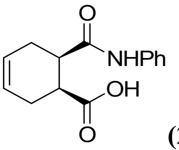


**(2i) Racemic**

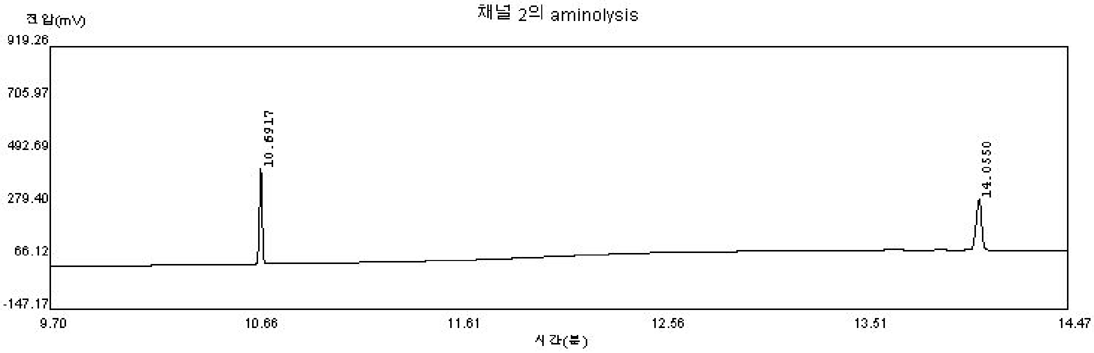


| Entry | RT [min] | Area [mV*s] | Shape | Width [sec] | Area % |
|---|---|---|---|---|---|
| 1 | 10.6917 | 367.8045 | PP | 7.3000 | 49.9758 |
| 2 | 14.0550 | 368.1607 | BV | 12.3000 | 50.0242 |

**Table 3.1**

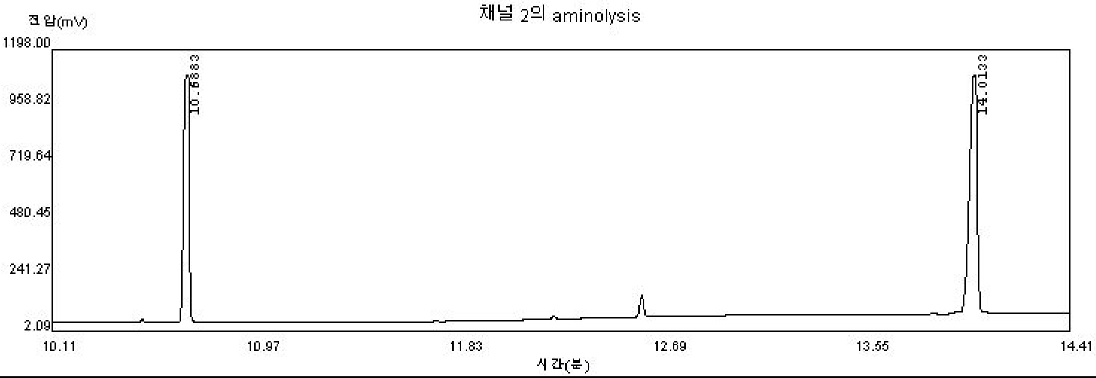


| Entry | RT [min] | Area [mV*s] | Shape | Width [sec] | Area % |
|---|---|---|---|---|---|
| 1 | 10.6883 | 1723.0880 | BB | 10.5000 | 42.7610 |
| 2 | 14.0133 | 2306.4941 | BP | 14.1000 | 57.2390 |

**Table 3.2**

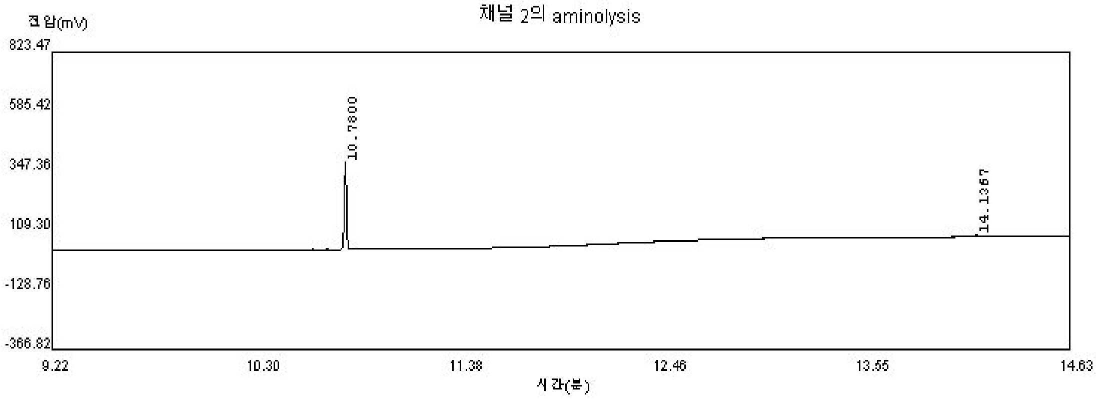


| Entry | RT [min] | Area [mV*s] | Shape | Width [sec] | Area % |
|---|---|---|---|---|---|
| | | | | | |

| | | | | | |
|---|---|---|---|---|---|
| 1 | 10.7800 | 337.5222 | BV | 8.8000 | 96.0488 |
| 2 | 14.1367 | 13.8848 | BB | 9.2000 | 3.9512 |

**Table 3.3**

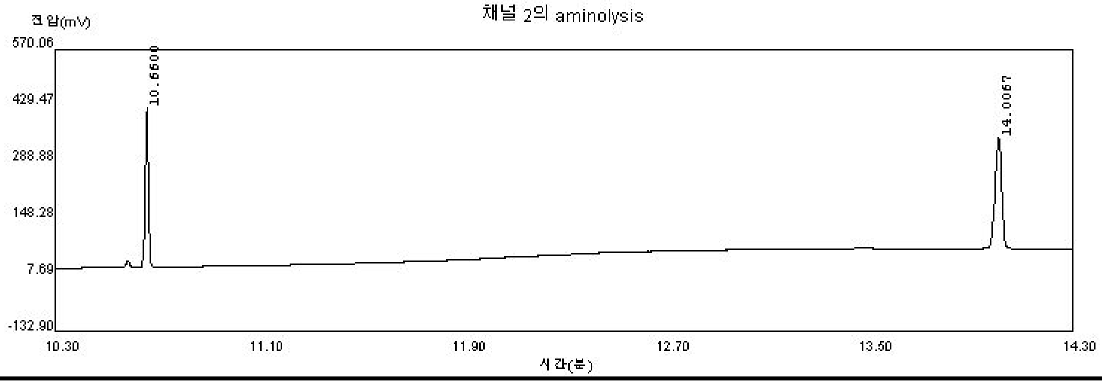


| Entry | RT [min] | Area [mV*s] | Shape | Width [sec] | Area % |
|---|---|---|---|---|---|
| 1 | 10.6600 | 378.4475 | BB | 5.4000 | 42.9214 |
| 2 | 14.0067 | 503.2744 | BP | 13.2000 | 57.0786 |

**Table 3.4**

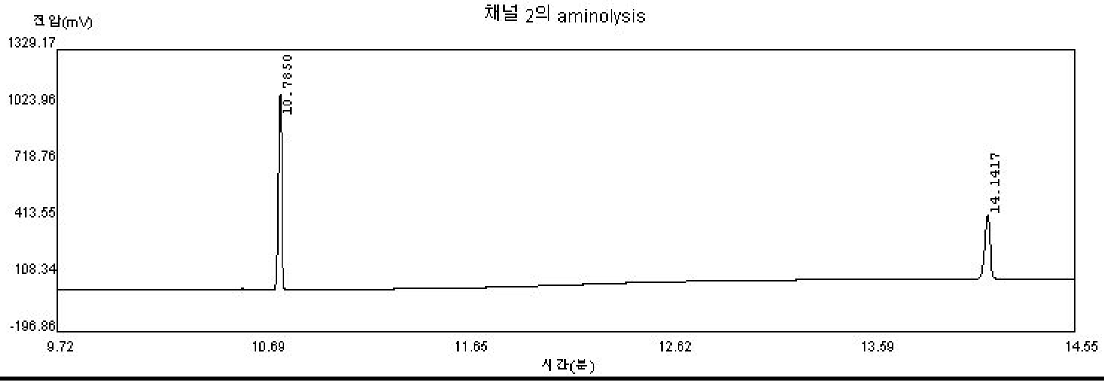


| Entry | RT [min] | Area [mV*s] | Shape | Width [sec] | Area % |
|---|---|---|---|---|---|
| 1 | 10.7850 | 1247.5061 | BP | 10.3000 | 67.0341 |
| 2 | 14.1417 | 613.4970 | BB | 13.2000 | 32.9659 |

**Table 3.5**

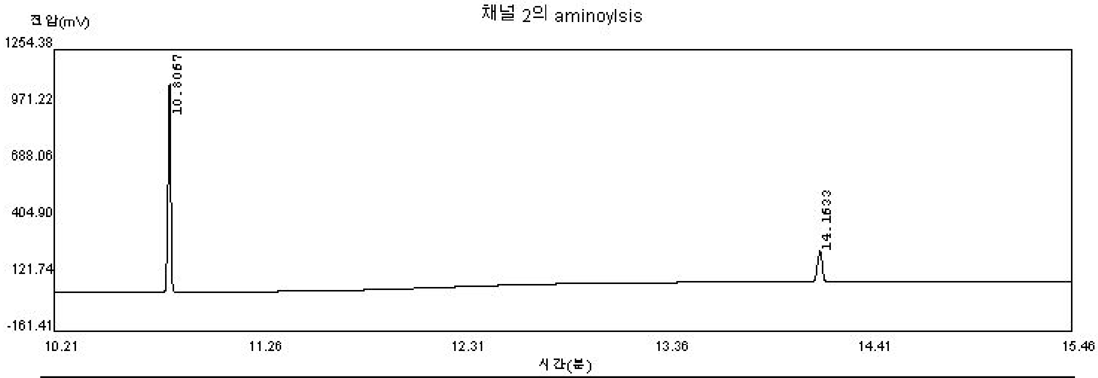


| Entry | RT [min] | Area [mV*s] | Shape | Width [sec] | Area % |
|---|---|---|---|---|---|
| 1 | 10.8067 | 1096.1391 | BP | 8.4000 | 80.1383 |
| 2 | 14.1633 | 271.6699 | BP | 13.1000 | 19.8617 |

**Table 3.6**

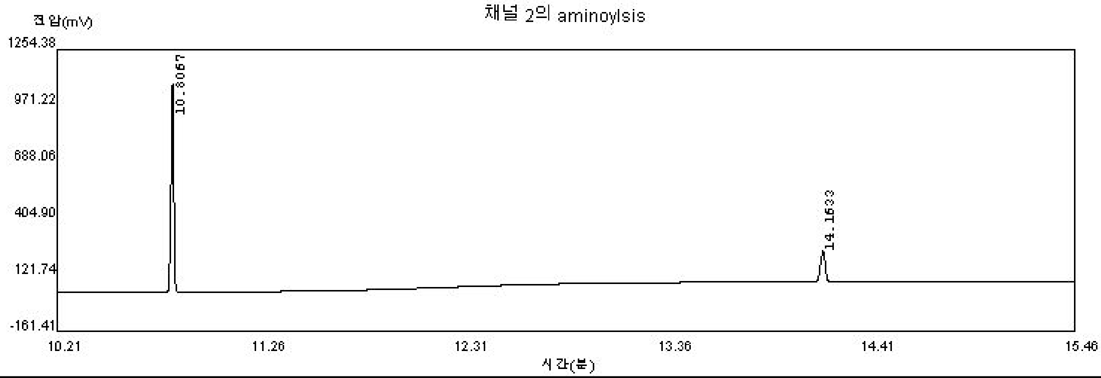


| 번호 | RT [min] | Area [mV*s] | Shape | Width [sec] | Area % |
|---|---|---|---|---|---|
| 1 | 10.8067 | 1096.1391 | BP | 8.4000 | 80.1383 |
| 2 | 14.1633 | 271.6699 | BP | 13.1000 | 19.8617 |

**Table 3.7**

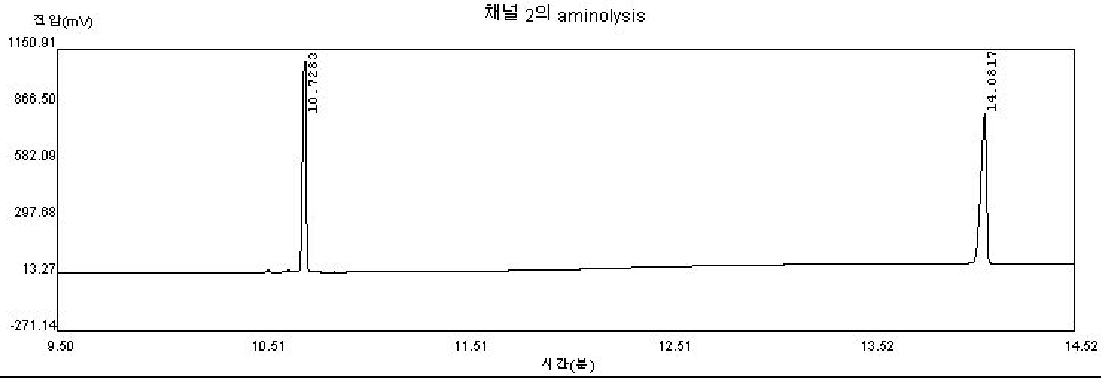


| Entry | RT [min] | Area [mV*s] | Shape | Width [sec] | Area % |
|---|---|---|---|---|---|
| 1 | 10.7283 | 1324.9902 | BB | 8.1000 | 47.0095 |
| 2 | 14.0817 | 1493.5710 | BV | 13.8000 | 52.9905 |

**Table 3.8**

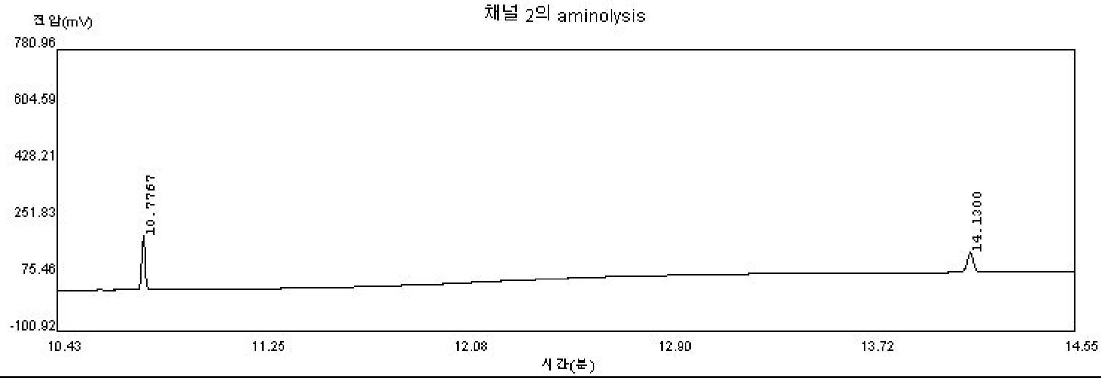


| Entry | RT [min] | Area [mV*s] | Shape | Width [sec] | Area % |
|---|---|---|---|---|---|
| 1 | 10.7767 | 160.6997 | VP | 7.3000 | 59.9391 |
| 2 | 14.1300 | 107.4052 | BB | 11.4000 | 40.0609 |

**Table 3.9**

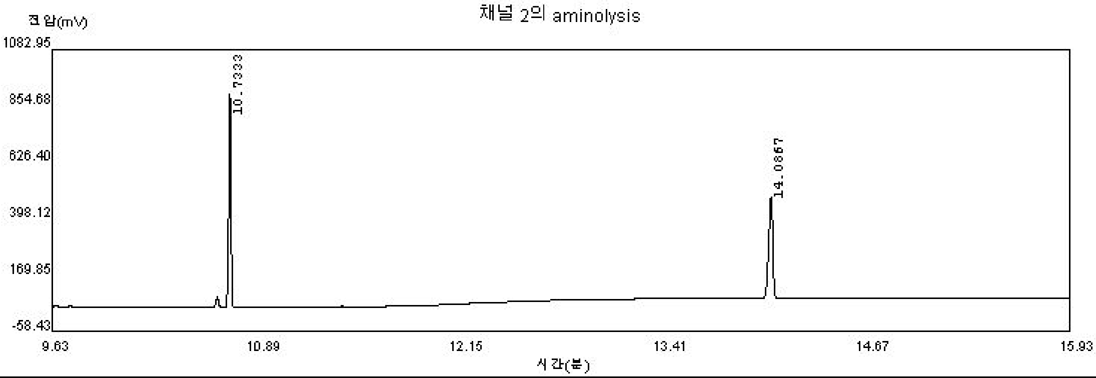


| Entry | RT [min] | Area [mV*s] | Shape | Width [sec] | Area % |
|---|---|---|---|---|---|
| 1 | 10.7250 | 1076.5404 | BP | 10.9000 | 42.5340 |
| 2 | 14.0817 | 1454.4684 | BP | 10.0000 | 57.4660 |

**Table 3.10**

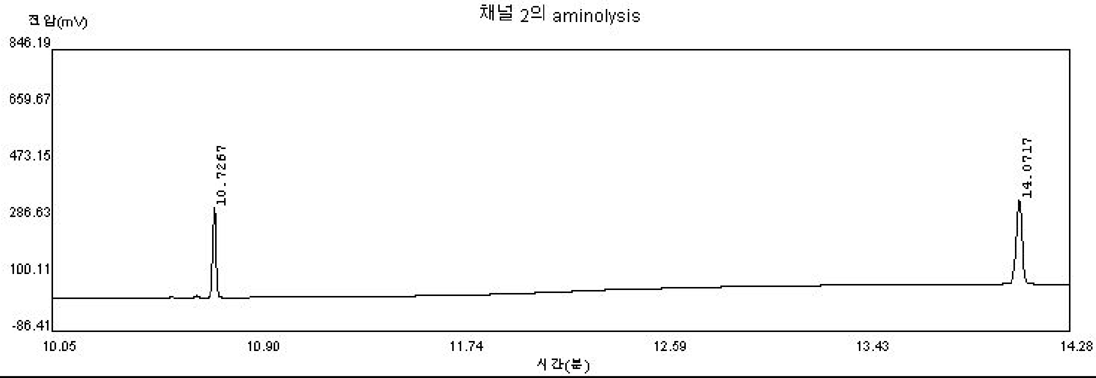


| Entry | RT [min] | Area [mV*s] | Shape | Width [sec] | Area % |
|---|---|---|---|---|---|
| 1 | 10.7267 | 287.6642 | BV | 6.1000 | 37.3138 |
| 2 | 14.0717 | 483.2687 | BB | 8.9000 | 62.6862 |

**Table 3.11**

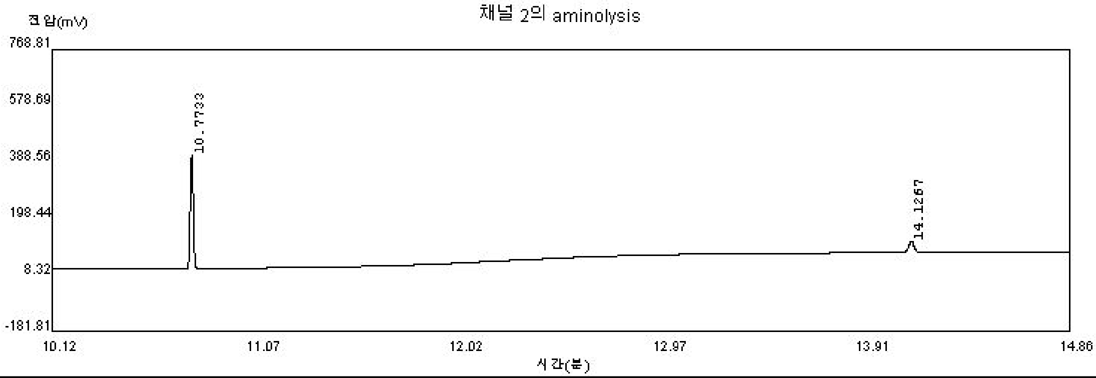


| Entry | RT [min] | Area [mV*s] | Shape | Width [sec] | Area % |
|---|---|---|---|---|---|
| 1 | 10.7733 | 365.6065 | VV | 6.6000 | 84.4786 |
| 2 | 14.1267 | 67.1734 | BB | 13.1000 | 15.5214 |

**Table 3.12**

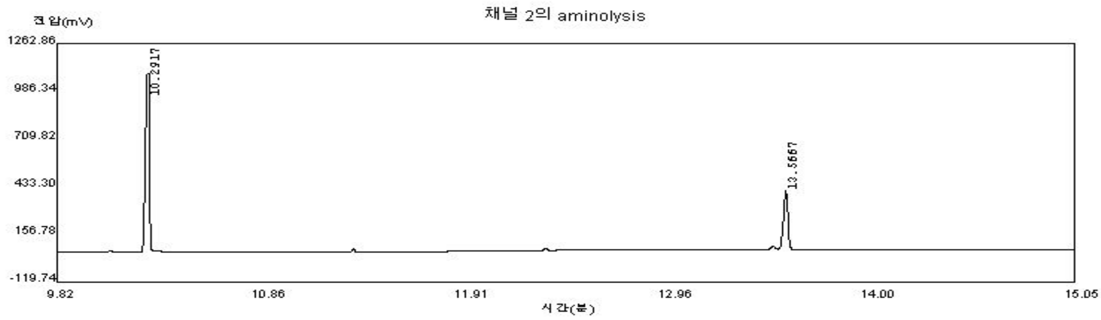


| 번호 | RT [min] | Area [mV*s] | Shape | Width [sec] | Area % |
|---|---|---|---|---|---|
| 1 | 10.2917 | 1501.0228 | BB | 6.0000 | 74.1390 |
| 2 | 13.5667 | 523.5842 | BP | 8.0000 | 25.8610 |

**Table 3.13**

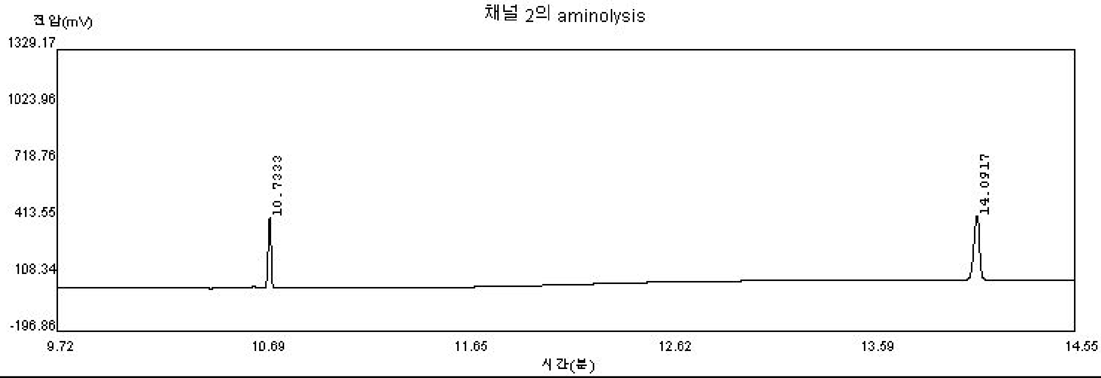


| Entry | RT [min] | Area [mV*s] | Shape | Width [sec] | Area % |
|---|---|---|---|---|---|
| 1 | 10.7333 | 364.6505 | BP | 8.4000 | 35.9087 |
| 2 | 14.0917 | 650.8427 | BP | 10.5000 | 64.0913 |

**Table 3.14**

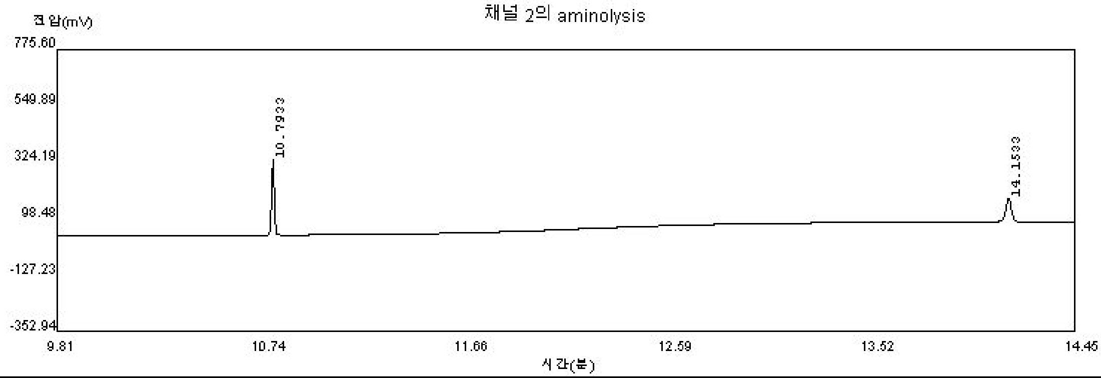


| Entry | RT [min] | Area [mV*s] | Shape | Width [sec] | Area % |
|---|---|---|---|---|---|
| 1 | 10.7933 | 283.5012 | VB | 10.1000 | 62.1293 |
| 2 | 14.1533 | 172.8071 | BB | 23.1000 | 37.8707 |

**Table 3.15**

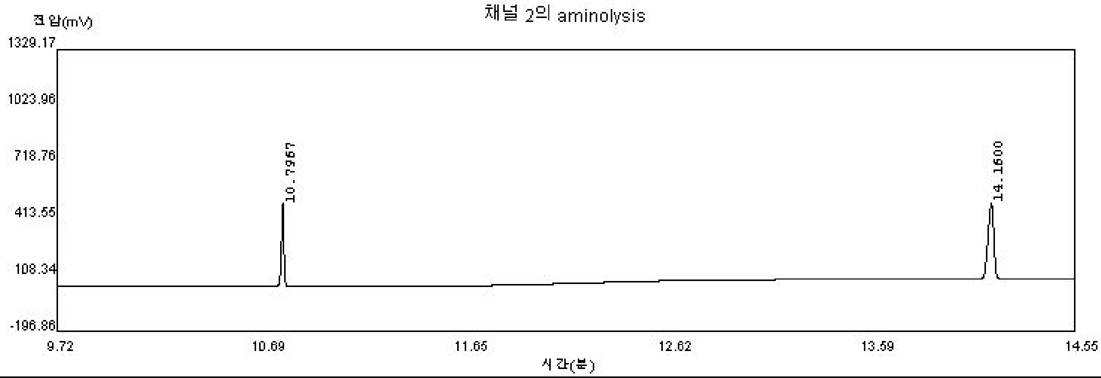


| Entry | RT [min] | Area [mV*s] | Shape | Width [sec] | Area % |
|---|---|---|---|---|---|
| 1 | 10.7967 | 421.7053 | BP | 6.5000 | 35.4204 |
| 2 | 14.1600 | 768.8659 | BB | 9.3000 | 64.5796 |

**Table 3.16**

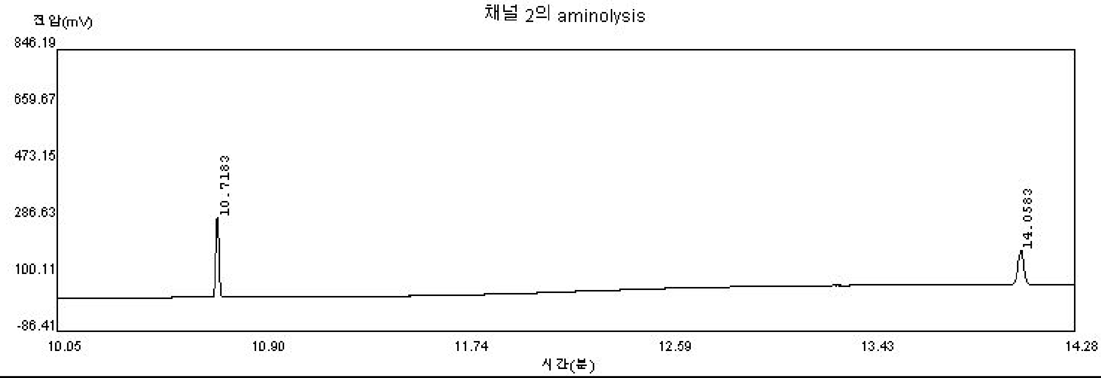


| Entry | RT [min] | Area [mV*s] | Shape | Width [sec] | Area % |
|---|---|---|---|---|---|
| 1 | 10.7183 | 251.6043 | BV | 6.5000 | 55.7189 |
| 2 | 14.0583 | 199.9561 | BB | 8.9000 | 44.2811 |

**Table 3.17**

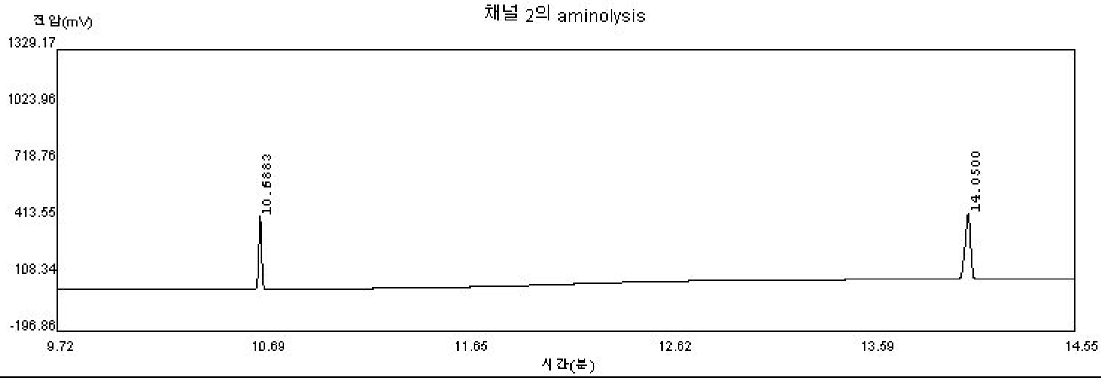


| Entry | RT [min] | Area [mV*s] | Shape | Width [sec] | Area % |
|---|---|---|---|---|---|
| 1 | 10.6883 | 374.9015 | BV | 7.2000 | 36.9807 |
| 2 | 14.0500 | 638.8760 | BB | 9.8000 | 63.0193 |

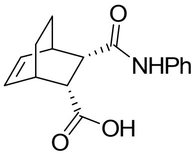


**(2j) Racemic**

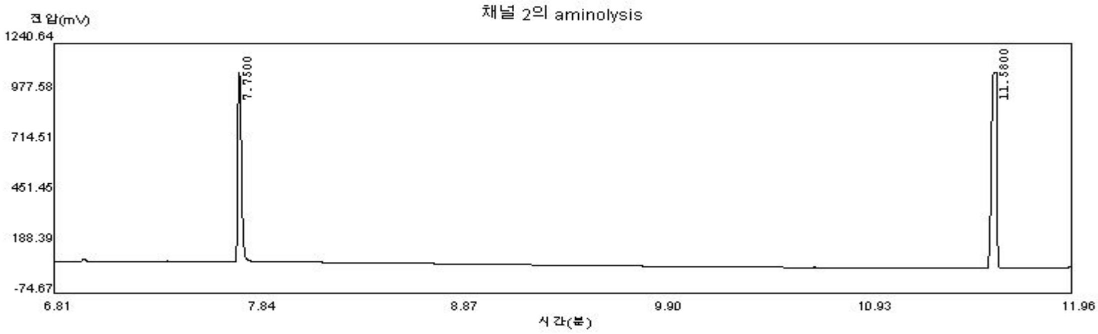


| Entry | RT [min] | Area [mV*s] | Shape | Width [sec] | Area % |
|---|---|---|---|---|---|
| 1 | 7.7500 | 1333.4750 | BB | 12.1000 | 49.2449 |
| 2 | 11.5800 | 1374.3672 | BB | 2.5000 | 50.7551 |

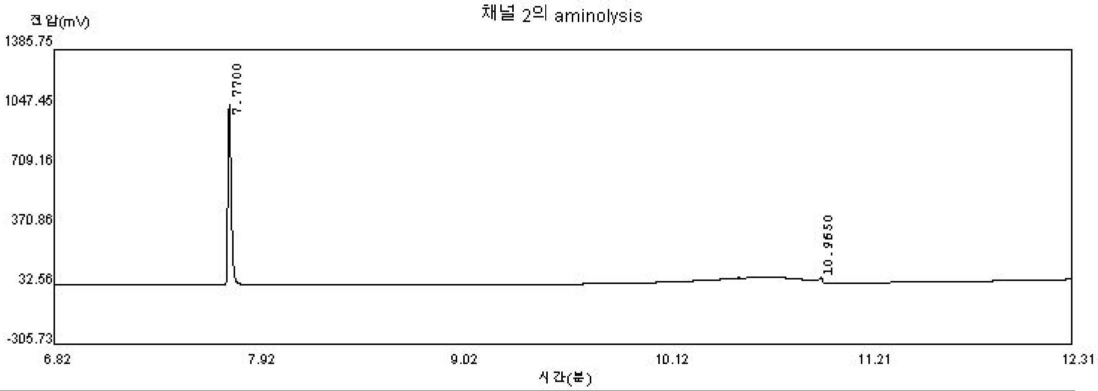


| Entry | RT [min] | Area [mV*s] | Shape | Width [sec] | Area % |
|---|---|---|---|---|---|
| 1 | 7.7700 | 1267.0443 | BP | 12.0000 | 97.6128 |
| 2 | 10.9650 | 30.9864 | BP | 3.3000 | 2.3872 |

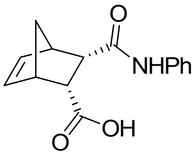


**(2k) Racemic**

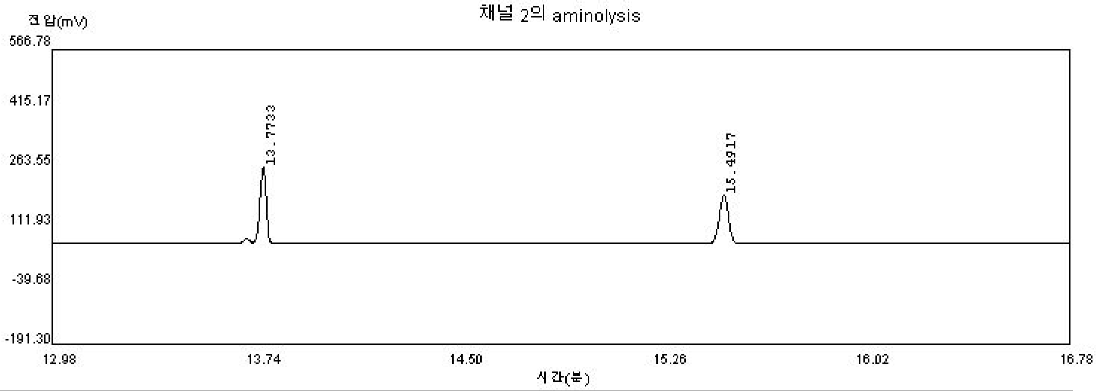


| Entry | RT [min] | Area [mV*s] | Shape | Width [sec] | Area % |
|---|---|---|---|---|---|
| 1 | 13.7733 | 300.4986 | BP | 8.5000 | 50.5979 |
| 2 | 15.4917 | 293.3970 | BP | 10.2000 | 49.4021 |

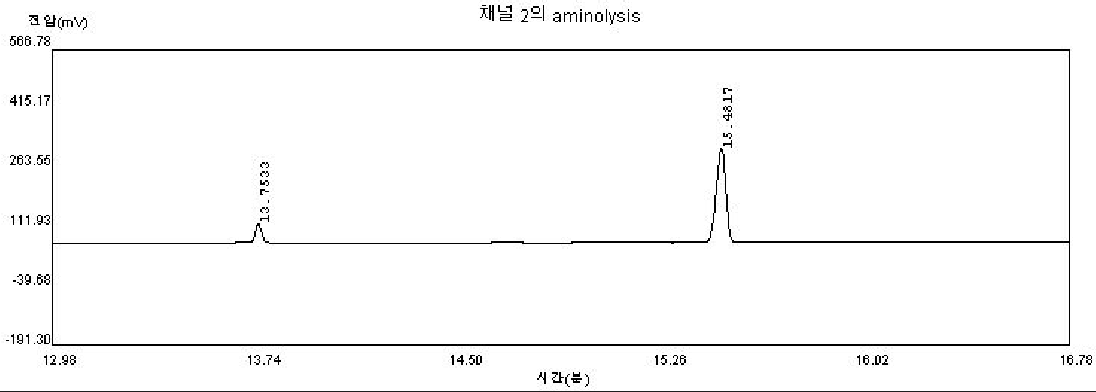


| Entry | RT [min] | Area [mV*s] | Shape | Width [sec] | Area % |
|---|---|---|---|---|---|
| 1 | 13.7533 | 31.6804 | BB | 2.2000 | 4.9813 |
| 2 | 15.4817 | 604.3064 | BP | 13.2000 | 95.0187 |

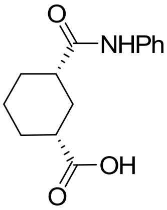


**(2l) Racemic**

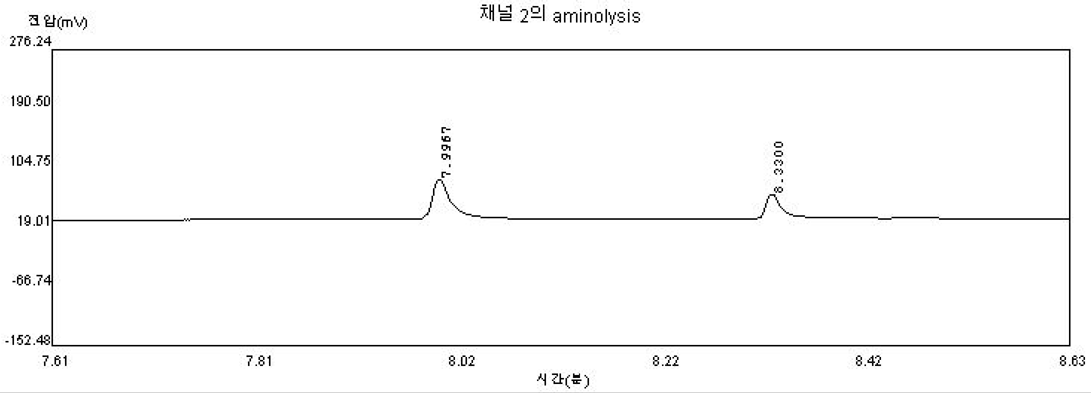


| Entry | RT [min] | Area [mV*s] | Shape | Width [sec] | Area % |
|---|---|---|---|---|---|
| 1 | 7.9967 | 39.2130 | BB | 4.0000 | 50.7866 |
| 2 | 8.3300 | 37.9983 | BP | 5.2000 | 49.2134 |

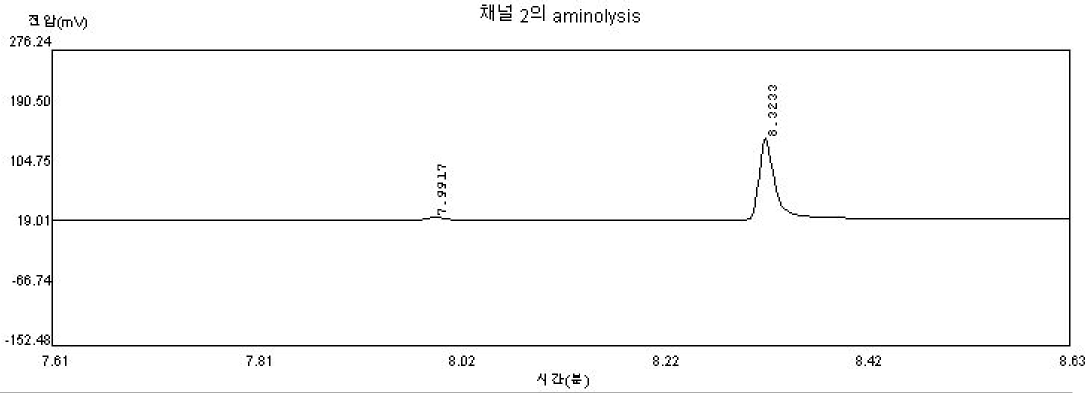


| Entry | RT [min] | Area [mV*s] | Shape | Width [sec] | Area % |
|---|---|---|---|---|---|
| 1 | 7.9917 | 4.0186 | BB | 4.6000 | 3.0802 |
| 2 | 8.3233 | 126.4465 | BB | 8.4000 | 96.9198 |

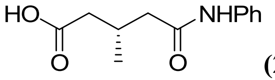


**(2m) Racemic**

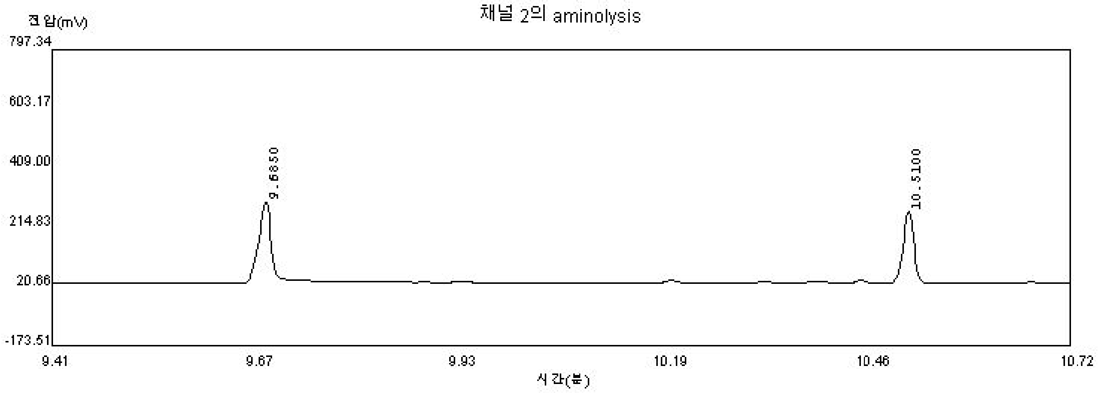


| Entry | RT [min] | Area [mV*s] | Shape | Width [sec] | Area % |
|---|---|---|---|---|---|
| 1 | 9.6850 | 228.6803 | BB | 4.9000 | 50.8230 |
| 2 | 10.5100 | 221.2741 | BB | 7.5000 | 49.1770 |

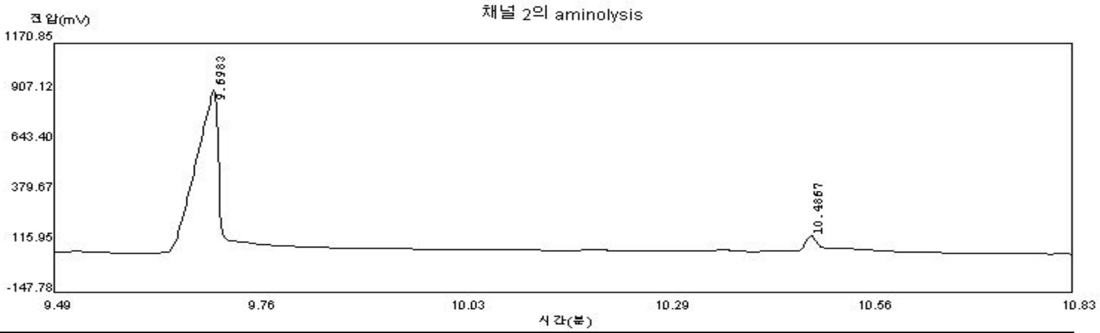


| Entry | RT [min] | Area [mV*s] | Shape | Width [sec] | Area % |
|---|---|---|---|---|---|
| 1 | 9.6983 | 1910.4851 | BB | 13.9000 | 97.1152 |
| 2 | 10.4867 | 56.7504 | BB | 6.0000 | 2.8848 |

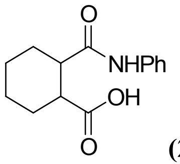


**(2n) Racemic**

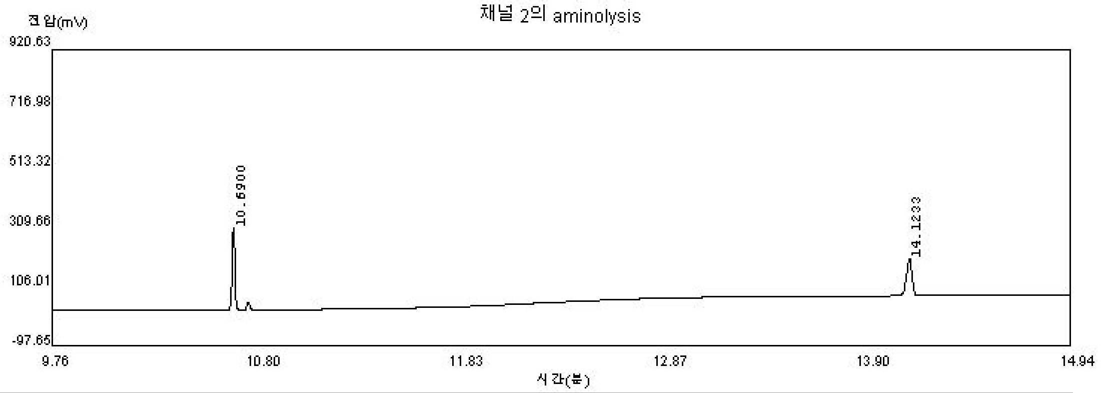


| Entry | RT [min] | Area [mV*s] | Shape | Width [sec] | Area % |
|---|---|---|---|---|---|
| 1 | 10.6900 | 270.5121 | BB | 4.8000 | 52.5893 |
| 2 | 14.1233 | 243.8741 | BB | 12.6000 | 47.4107 |

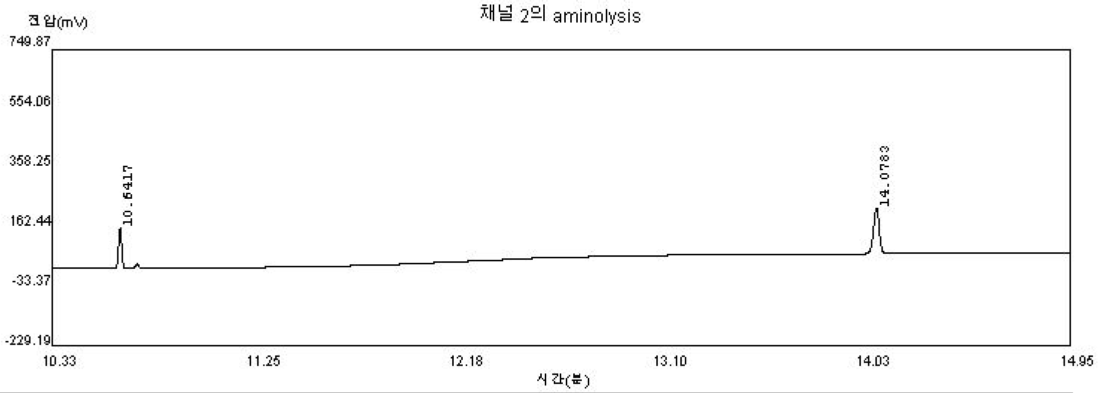


| Entry | RT [min] | Area [mV*s] | Shape | Width [sec] | Area % |
|---|---|---|---|---|---|
| 1 | 10.6417 | 40.9492 | BB | 5.5000 | 23.1922 |
| 2 | 14.0783 | 135.6156 | BB | 7.6000 | 76.8078 |

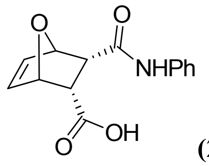


**(2o) Racemic**

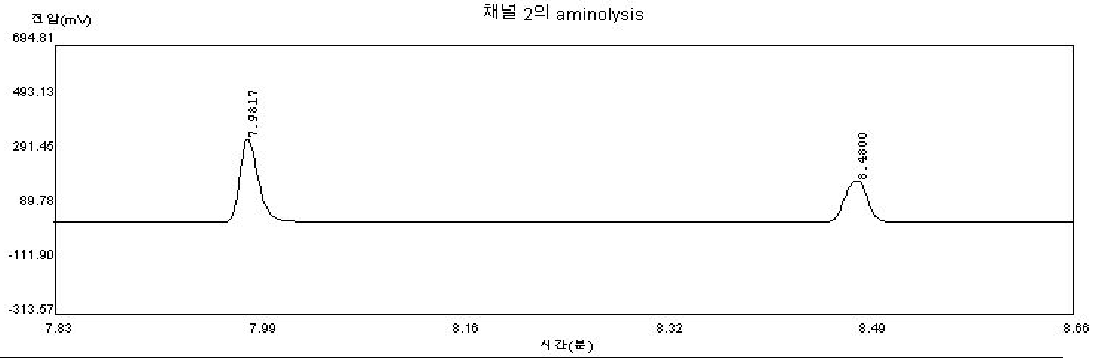


| Entry | RT [min] | Area [mV*s] | Shape | Width [sec] | Area % |
|---|---|---|---|---|---|
| **1** | **7.9817** | **182.7999** | **BB** | **7.0000** | **48.2490** |
| **2** | **8.4800** | **196.0679** | **BV** | **7.4000** | **51.7510** |

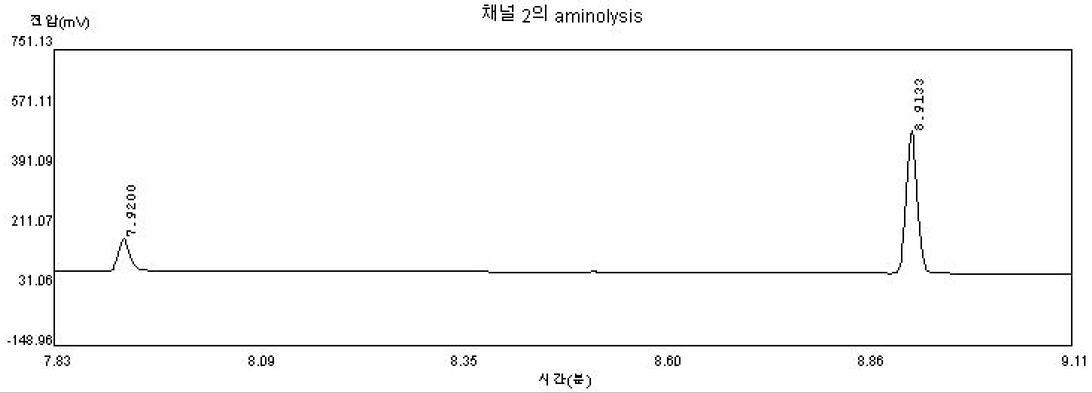


| Entry | RT [min] | Area [mV*s] | Shape | Width [sec] | Area % |
|---|---|---|---|---|---|
| **1** | **7.9200** | **96.4617** | **BB** | **13.9000** | **18.7836** |
| **2** | **8.9133** | **417.0810** | **BB** | **11.0000** | **81.2164** |

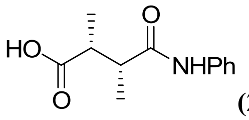


**(2p) Racemic**

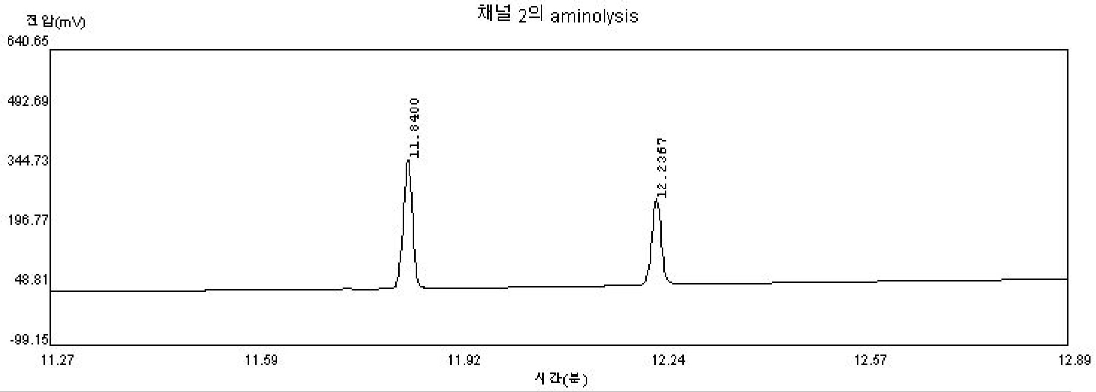


| Entry | RT [min] | Area [mV*s] | Shape | Width [sec] | Area % |
|---|---|---|---|---|---|
| 1 | 11.8400 | 172.0444 | BB | 2.4000 | 50.8499 |
| 2 | 12.2367 | 166.2932 | BB | 2.4000 | 49.1501 |

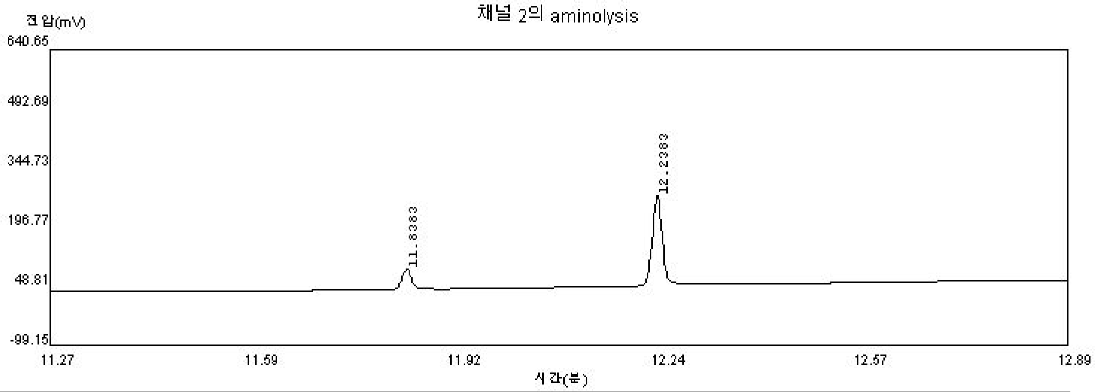


| Entry | RT [min] | Area [mV*s] | Shape | Width [sec] | Area % |
|---|---|---|---|---|---|
| 1 | 11.8383 | 32.4240 | BB | 2.4000 | 17.4205 |
| 2 | 12.2383 | 153.7018 | BB | 2.4000 | 82.5795 |

## Nonlinear effect

**Table 4.1**

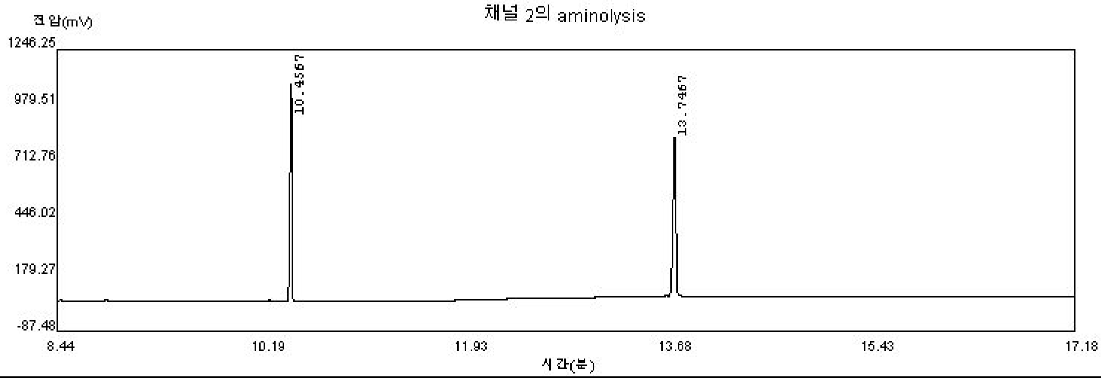


| Entry | RT [min] | Area [mV*s] | Shape | Width [sec] | Area % |
|---|---|---|---|---|---|
| 1 | 10.4567 | 1210.7259 | BB | 3.2000 | 50.3709 |
| 2 | 13.7467 | 1192.8941 | BP | 7.9000 | 49.6291 |

**Table 4.2**

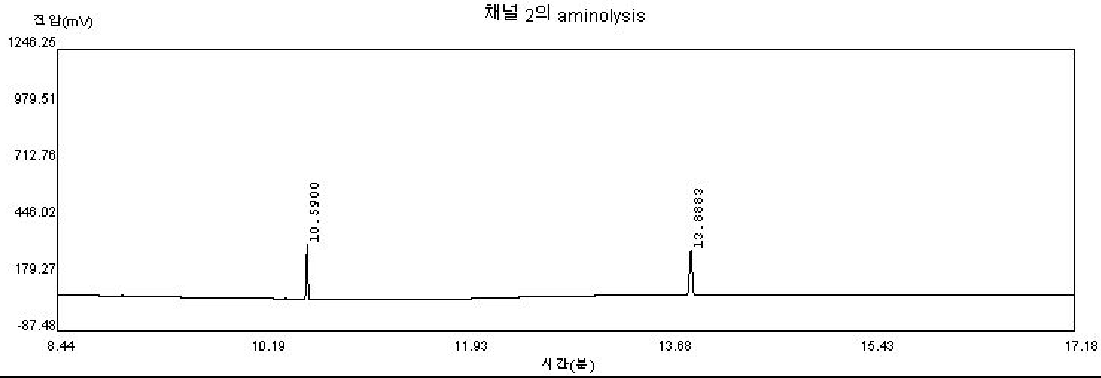


| Entry | RT [min] | Area [mV*s] | Shape | Width [sec] | Area % |
|---|---|---|---|---|---|
| 1 | 10.5900 | 239.7809 | BB | 4.4000 | 41.3492 |
| 2 | 13.8883 | 340.1117 | BP | 9.6000 | 58.6508 |

**Table 4.3**

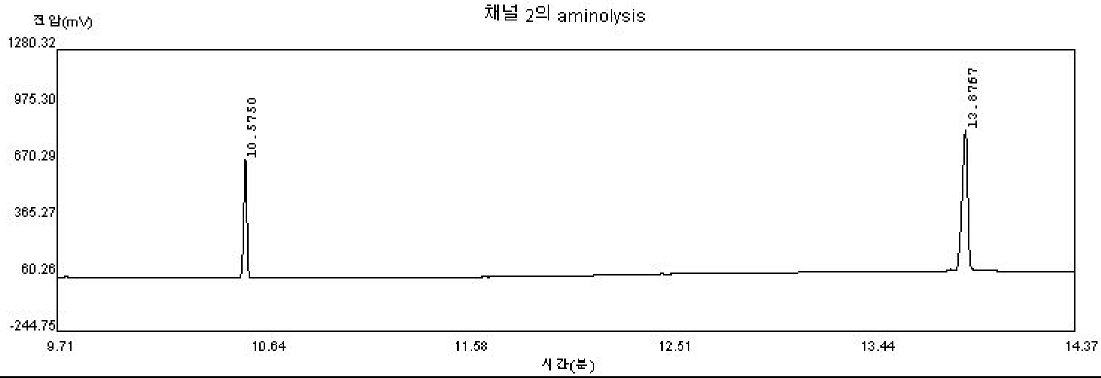


| Entry | RT [min] | Area [mV*s] | Shape | Width [sec] | Area % |
|---|---|---|---|---|---|
| 1 | 10.5750 | 609.0834 | BB | 3.7000 | 31.5697 |
| 2 | 13.8767 | 1320.2461 | BP | 8.1000 | 68.4303 |

**Table 4.4**

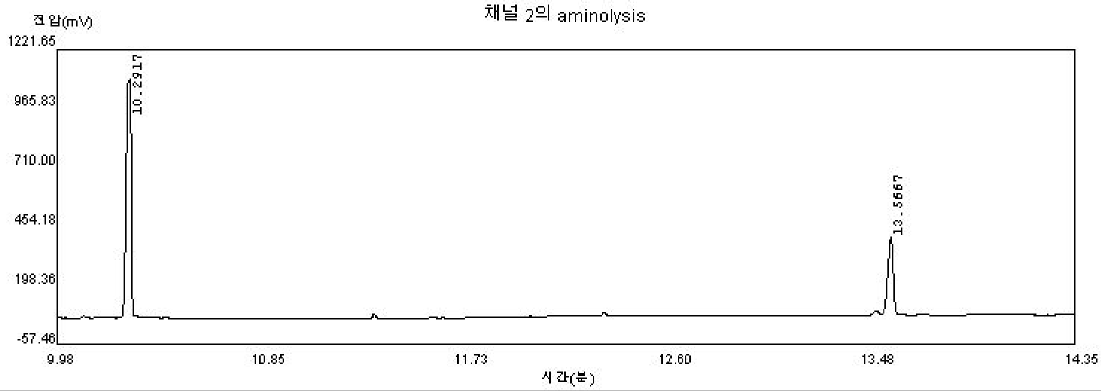


| Entry | RT [min] | Area [mV*s] | Shape | Width [sec] | Area % |
|---|---|---|---|---|---|
| 1 | 10.2917 | 1501.0228 | BB | 6.0000 | 74.1390 |
| 2 | 13.5667 | 523.5842 | BP | 8.0000 | 25.8610 |

**Table 4.5**

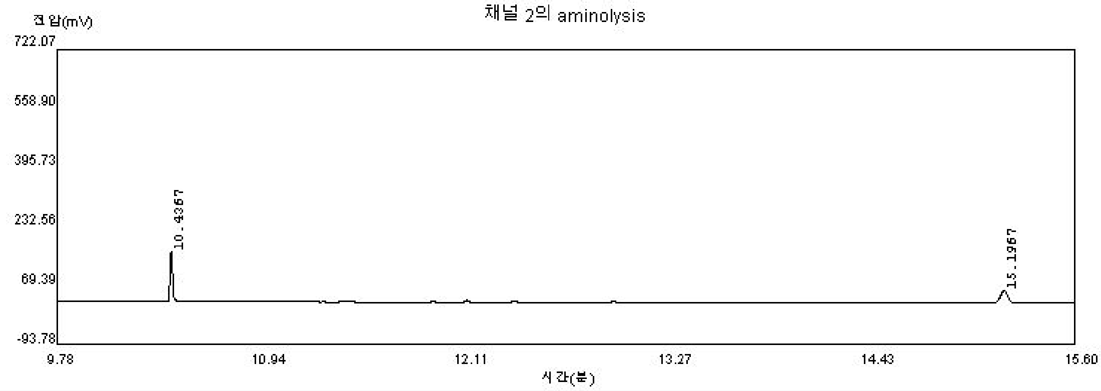


| Entry | RT [min] | Area [mV*s] | Shape | Width [sec] | Area % |
|---|---|---|---|---|---|
| 1 | 10.4367 | 129.0729 | BB | 2.8000 | 78.1975 |
| 2 | 15.1967 | 35.9872 | BB | 3.0000 | 21.8025 |

**Table 4.6**

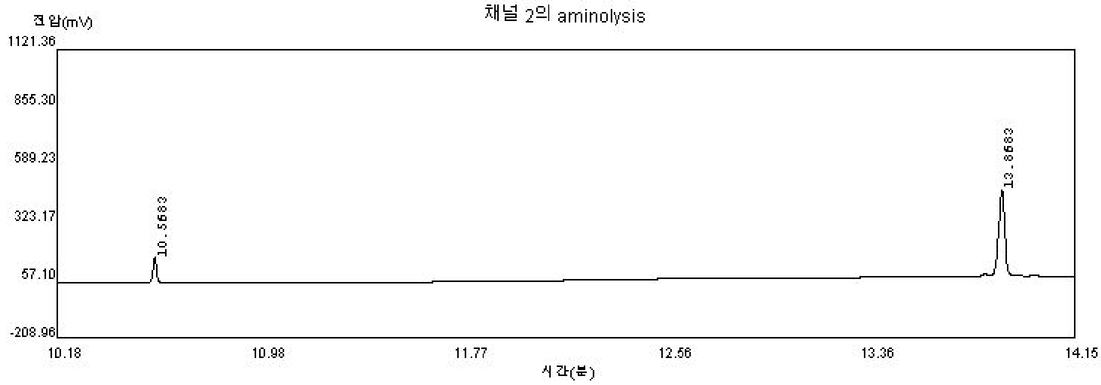


| Entry | RT [min] | Area [mV*s] | Shape | Width [sec] | Area % |
|---|---|---|---|---|---|
| 1 | 10.5683 | 105.8459 | BB | 3.7000 | 13.9911 |
| 2 | 13.8683 | 650.6746 | BP | 8.1000 | 86.0089 |

**Table 4.7**

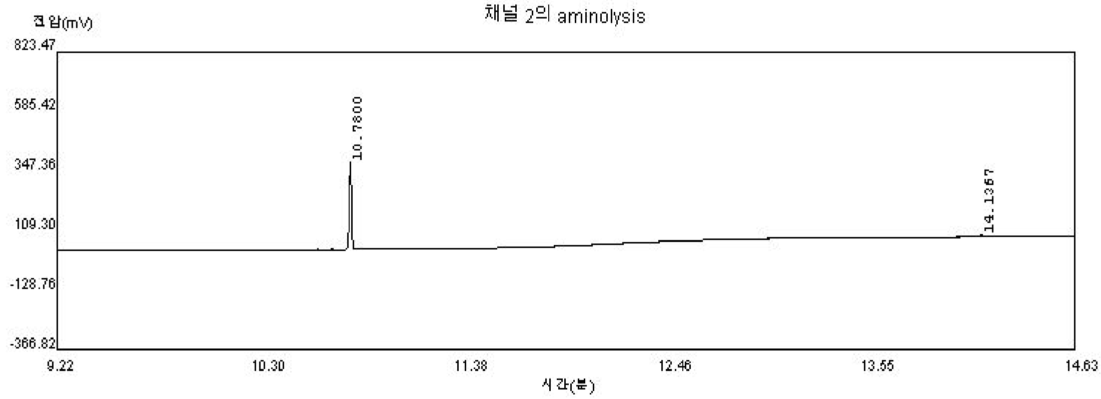


| Entry | RT [min] | Area [mV*s] | Shape | Width [sec] | Area % |
|---|---|---|---|---|---|
| 1 | 10.7800 | 337.5222 | BV | 8.8000 | 96.0488 |
| 2 | 14.1367 | 13.8848 | BB | 9.2000 | 3.9512 |

## 6. Computational Results of DFT Calculations for all Calculated Structures

### Fig 1. Thiolysis

### TS1

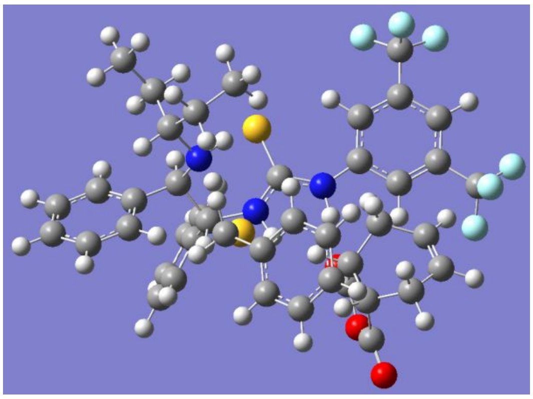

Temperature = 298.15 Kelvin

Pressure = 1 atm

Frequencies scaled by = 1

Symbolic Z-matrix:

Charge = 0 Multiplicity = 1

C -5.11901 0.57485 0.17383

C -4.83594 -0.80002 0.23358

C -5.87628 -1.70784 0.46359

C -7.18724 -1.25477 0.62751

C -7.46724 0.1108 0.55731

C -6.43146 1.02231 0.33081

H -4.30755 1.28184 0.00602

H -5.65775 -2.76805 0.53265

H -7.98385 -1.96569 0.81243

H -8.48425 0.46386 0.68243

H -6.64451 2.08386 0.28104

C -2.93922 -1.51783 2.56219

C -3.67567 -0.81274 3.52208

C -4.06649 -1.43211 4.71075

C -3.71983 -2.76262 4.95165

C -2.98291 -3.4704 3.99826

C -2.59435 -2.85444 2.80798

H -3.948 0.22019 3.33572

H -4.63556 -0.87537 5.44604

H -4.0185 -3.24385 5.87573

H -2.70722 -4.5025 4.18173

H -2.01944 -3.40137 2.07085

C -3.41576 -1.31195 0.07783

C -2.53605 -0.81169 1.27027

N -1.06092 -0.81106 1.04223

N -2.77969 -0.82955 -1.23574

H -2.75533 0.25447 1.37336

H -3.39278 -2.39958 0.04569

H -1.94437 -1.44111 -1.36163

H -0.62086 0.03556 1.41431

C -0.2167 -1.75016 0.54228

C 2.45214 -3.12789 -0.5065

C 3.72834 -3.57469 -0.83628

C 4.87534 -2.94962 -0.36558

C 4.72098 -1.84331 0.46201

C 3.46296 -1.38107 0.81502

C 2.30152 -2.00929 0.32515

H 1.58719 -3.65738 -0.87048

H 5.85694 -3.32869 -0.6092

H 3.37398 -0.53869 1.48984

N 1.09308 -1.41427 0.72651

S -0.77309 -3.18977 -0.29881

H 1.24175 -0.53011 1.24788

F 7.03714 -1.88815 0.9305

F 5.72603 -0.59297 2.19982

F 6.19969 -0.00657 0.12373

F 3.90788 -4.31348 -3.09348

F 5.02561 -5.41698 -1.54016

F 2.79748 -5.58343 -1.66804

C 5.91622 -1.09837 0.93829

C 3.86202 -4.72222 -1.77892

C 1.52588 2.17019 1.79588

C 1.93187 4.30284 0.74451

C 2.03359 4.38319 2.25864

O 1.31735 0.98291 2.06446

C -2.33453 3.45521 -1.66655

S -1.75994 2.04245 -0.49596

H -3.25584 3.87587 -1.25819

H -2.43589 0.20383 -1.10564

C -1.27927 4.5236 -1.78982

C -1.27335 5.62913 -0.92544

C -0.27655 4.43512 -2.76794

C -0.2986 6.62387 -1.04051

H -2.04076 5.70697 -0.16261

C 0.69667 5.42893 -2.88937

H -0.26761 3.5822 -3.43834

C 0.68868 6.53013 -2.02692

H -0.31799 7.47807 -0.37224

H 1.45249 5.35262 -3.66353

H 1.43438 7.31026 -2.12924

H -2.54674 3.01766 -2.64532

O 1.73854 3.0755 2.81698

C 1.55692 2.83986 0.44327

O 2.32397 5.29983 2.98552

C 2.55109 2.11379 -0.50609

C 3.29374 4.75556 0.13121

C 3.97963 2.39592 -0.08348

C 4.32272 3.64857 0.22947

H 2.36458 2.48681 -1.52196

H 2.32893 1.04333 -0.50566

H 3.10853 5.01573 -0.91889

H 3.62031 5.66116 0.65164

H 4.69348 1.58157 -0.04404

H 5.33074 3.90032 0.54216

H 0.53404 2.75086 0.03151

H 1.14696 4.98954 0.41073

C -3.64609 -0.90863 -2.50015

H -4.55329 -0.34923 -2.25999

C -4.01089 -2.37326 -2.80672

C -2.88189 -0.17785 -3.62949

H -4.54389 -2.78851 -1.94484

H -3.09734 -2.96206 -2.94456

H -3.56576 -0.07445 -4.47904

H -2.64541 0.83248 -3.27611

C -1.5837 -0.87637 -4.09001

C -4.91861 -2.49546 -4.05057

H -0.8652 -0.97792 -3.26956

H -1.78597 -1.87192 -4.49734

H -1.10354 -0.2827 -4.87487

H -5.23107 -3.53582 -4.18566

H -5.81923 -1.88176 -3.93356

H -4.39719 -2.17836 -4.95892

**TS2(major)**

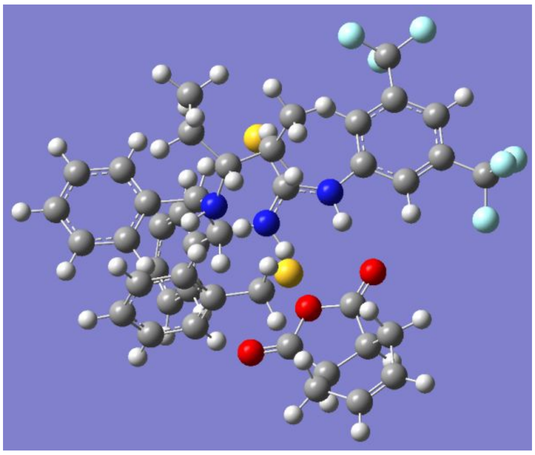

Temperature = 298.15 Kelvin

Pressure = 1 atm

Frequencies scaled by = 1

Symbolic Z-matrix:

Charge = 0 Multiplicity = 1

C -4.36814 -1.11691 0.82432

C -3.36122 -2.09254 0.87019

C -3.67775 -3.38795 1.29816

C -4.98143 -3.70599 1.68116

C -5.98124 -2.73228 1.63265

C -5.67237 -1.44061 1.20079

H -4.14434 -0.10896 0.48733

H -2.90035 -4.14219 1.342

H -5.21468 -4.70992 2.01569

H -6.99431 -2.979 1.92811

H -6.44392 -0.68225 1.15165

C -1.31826 -1.2331 2.91389

C -1.77633 -0.32925 3.88477

C -1.80621 -0.69293 5.2318

C -1.37895 -1.96271 5.62467

C -0.91319 -2.86248 4.66379

C -0.87906 -2.50216 3.31527

H -2.09087 0.66404 3.58229

H -2.16403 0.01455 5.97113

H -1.40386 -2.24728 6.67043

H -0.56768 -3.84542 4.96305

H -0.4795 -3.1895 2.58317

C -1.94008 -1.7552 0.4567

C -1.30143 -0.75436 1.46006

N 0.02621 -0.2259 1.03972

N -1.97976 -1.10431 -0.94981

H -1.93208 0.13999 1.42294

H -1.32729 -2.65012 0.37901

H -2.92078 -1.25712 -1.34855

H 0.06118 0.79049 1.17319

C 1.18797 -0.86775 0.76117

C 4.27069 -1.39657 0.22383

C 5.631 -1.42155 -0.07069

C 6.33726 -0.27268 -0.39765

C 5.645 0.93327 -0.41581

C 4.29417 0.99246 -0.11158

C 3.57729 -0.17767 0.21202

H 3.75292 -2.31605 0.4403

H 7.3914 -0.3131 -0.63017

H 3.79023 1.9502 -0.11437

N 2.21544 0.01098 0.51222

S 1.33144 -2.60869 0.66582

H 1.95727 1.00918 0.49211

F 7.68342 2.11918 -0.51401

F 5.79382 3.28545 -0.23576

F 6.27759 2.39019 -2.19516

F 5.54011 -3.7777 -0.25789

F 7.442 -2.74018 -0.81622

F 6.85568 -2.93662 1.30389

C 6.34696 2.1767 -0.83605

C 6.36131 -2.71596 0.03754

C 0.70414 3.37845 0.81494

C -1.17149 4.7628 1.49126

C -1.32147 3.33831 1.99748

O 1.71232 2.88199 0.33658

C -2.93644 2.33615 -2.48797

S -1.79313 2.00432 -0.97718

H -2.36188 2.14914 -3.39756

H -1.88577 -0.00553 -0.87498

C -4.16991 1.47281 -2.45015

C -4.26803 0.31908 -3.24411

C -5.25074 1.80102 -1.61559

C -5.40917 -0.48968 -3.2005

H -3.45539 0.07229 -3.92092

C -6.39497 1.00399 -1.58066

H -5.18338 2.68693 -0.99361

C -6.47732 -0.14914 -2.36888

H -5.46999 -1.37218 -3.82803

H -7.22713 1.28371 -0.94364

H -7.36578 -0.76874 -2.33975

H -3.20524 3.39436 -2.45205

O -0.13225 2.59487 1.63113

C 0.15822 4.78501 0.69863

O -2.19419 2.80611 2.63991

C -0.01167 5.18802 -0.80197

C -2.4165 5.18426 0.64685

C -0.92702 6.39204 -0.8926

C -2.06952 6.39106 -0.20157

H 0.87627 5.46981 1.16204

H -1.10763 5.42033 2.36509

H -2.6667 4.32807 0.00601

H -3.25582 5.38007 1.31885

H 0.97268 5.38283 -1.23445

H -0.46229 4.3277 -1.3156

H -0.63538 7.22634 -1.52135

H -2.76247 7.22491 -0.23476

C -1.50141 -2.5465 -3.00902

H -2.3698 -2.05757 -3.47758

C -1.99535 -3.87924 -2.38319

C -0.4355 -2.75264 -4.11842

H -0.13837 -1.76608 -4.49815

H 0.45958 -3.21003 -3.68089

H -2.72538 -3.65726 -1.59319

H -2.54346 -4.43534 -3.15312

C -0.93995 -3.61114 -5.29786

C -0.87832 -4.77989 -1.80941

H -0.18093 -3.65797 -6.08607

H -1.15942 -4.63521 -4.97947

H -1.85206 -3.1801 -5.72752

H -0.20388 -5.12215 -2.60097

H -0.27777 -4.26023 -1.05548

H -1.3192 -5.66723 -1.34085

**Fig 2. Aminolysis**

**Anilin**

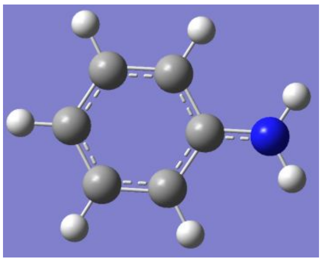

Temperature = 298.15 Kelvin

Pressure = 1 atm

Frequencies scaled by = 1

Electronic Energy (EE) = -286.02407 Hartree

Zero-point Energy Correction = 0.117752 Hartree

Thermal Correction to Energy = 0.123607 Hartree

Thermal Correction to Enthalpy = 0.124552 Hartree

Thermal Correction to Free Energy = 0.088542 Hartree

EE + Zero-point Energy = -285.90632 Hartree

EE + Thermal Energy Correction = -285.90046 Hartree

EE + Thermal Enthalpy Correction = -285.89952 Hartree

EE + Thermal Free Energy Correction = -285.93552 Hartree

E (Thermal) = 77.565 kcal/mol

Heat Capacity (Cv) = 23.123 cal/mol-kelvin

Entropy (S) = 75.788 cal/mol-kelvin

Symbolic Z-matrix:

Charge = 0 Multiplicity = 1

H -2.84361 -0.86766 0.00038

C -0.94725 0. -0.00003

C -0.21819 -1.20809 -0.00001

C -0.21819 1.20809 -0.00001

C 1.17351 -1.20223 0.00001

H -0.75517 -2.1509 -0.00003

C 1.17351 1.20223 0.00001

H -0.75517 2.1509 -0.00003

C 1.88581 0. 0.00002

H 1.70561 -2.14731 0.00002

H 1.70561 2.14731 0.00002

H 2.9687 0. 0.00003

N -2.32536 0. -0.00009

H -2.84361 0.86767 0.00039

**Anhydride**

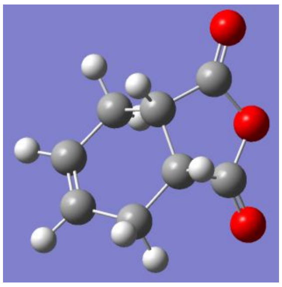

Temperature = 298.15 Kelvin

Pressure = 1 atm

Frequencies scaled by = 1

Electronic Energy (EE) = -532.39814 Hartree

Zero-point Energy Correction = 0.149948 Hartree

Thermal Correction to Energy = 0.158631 Hartree

Thermal Correction to Enthalpy = 0.159576 Hartree

Thermal Correction to Free Energy = 0.115693 Hartree

EE + Zero-point Energy = -532.24819 Hartree

EE + Thermal Energy Correction = -532.23951 Hartree

EE + Thermal Enthalpy Correction = -532.23856 Hartree

EE + Thermal Free Energy Correction = -532.28245 Hartree

E (Thermal) = 99.543 kcal/mol

Heat Capacity (Cv) = 33.571 cal/mol-kelvin

Entropy (S) = 92.358 cal/mol-kelvin

Symbolic Z-matrix:

Charge = 0 Multiplicity = 1

C 1.70627 0.58502 0.1936

C -0.28541 -0.56883 0.82696

C 0.51583 -1.38953 -0.18214

O 2.69227 1.27952 0.26139

O 1.75249 -0.72287 -0.39739

C 0.25512 0.85325 0.5738

O 0.24042 -2.43525 -0.72518

C -0.43774 1.51162 -0.65635

C -1.80558 -0.79229 0.71619

C -1.94046 1.34156 -0.60974

C -2.53712 0.3137 -0.00843

H 0.19185 1.50626 1.44367

H 0.06113 -0.89741 1.81745

H -1.95954 -1.74699 0.1929

H -2.2375 -0.90778 1.71806

H -0.16483 2.57125 -0.69035

H -0.04551 1.0493 -1.57428

H -2.53185 2.09531 -1.12095

H -3.62055 0.23181 -0.02043

**Cat.**

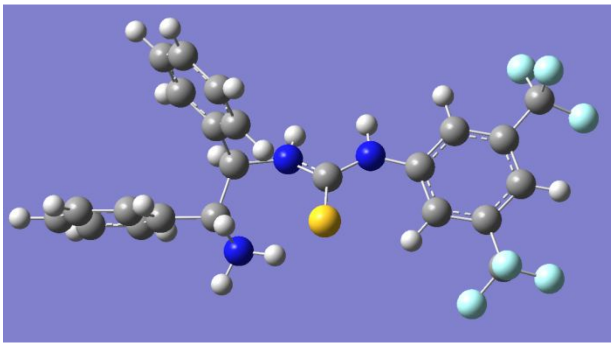

Temperature = 298.15 Kelvin

Pressure = 1 atm

Frequencies scaled by = 1

Electronic Energy (EE) = -2038.4342 Hartree

Zero-point Energy Correction = 0.390498 Hartree

Thermal Correction to Energy = 0.419537 Hartree

Thermal Correction to Enthalpy = 0.420482 Hartree

Thermal Correction to Free Energy = 0.325242 Hartree

EE + Zero-point Energy = -2038.0437 Hartree

EE + Thermal Energy Correction = -2038.0147 Hartree

EE + Thermal Enthalpy Correction = -2038.0138 Hartree

EE + Thermal Free Energy Correction = -2038.109 Hartree

E (Thermal) = 263.264 kcal/mol

Heat Capacity (Cv) = 110.445 cal/mol-kelvin

Entropy (S) = 200.448 cal/mol-kelvin

Symbolic Z-matrix:

Charge = 0 Multiplicity = 1

C 5.35849 1.33787 -1.80781

C 4.82708 1.14638 -0.52483

C 5.69825 0.95831 0.5539

C 7.08048 0.9594 0.3547

C 7.60459 1.1496 -0.92613

C 6.73965 1.33999 -2.00723

H 4.67012 1.50429 -2.62759

H 5.29272 0.8047 1.54752

H 7.74614 0.81433 1.19782

H 8.67773 1.1541 -1.07954

H 7.1414 1.49523 -3.00239

C 3.29335 -1.35884 0.35108

C 4.2752 -2.28379 -0.02492

C 4.73342 -3.24351 0.88009

C 4.21062 -3.28913 2.17354

C 3.23141 -2.36846 2.55692

C 2.77436 -1.40784 1.65364

H 4.69377 -2.24224 -1.0248

H 5.49665 -3.95033 0.57561

H 4.56373 -4.03365 2.87769

H 2.82295 -2.39699 3.56063

H 2.01493 -0.69495 1.95103

C 3.3155 1.12842 -0.32606

C 2.81394 -0.3166 -0.65186

N 1.33778 -0.45595 -0.89719

N 2.68733 2.05148 -1.30251

H 3.24995 -0.54539 -1.62815

H 3.07939 1.35208 0.7202

H 3.151 2.9666 -1.25754

H 1.69517 2.17017 -1.06525

H 1.14702 -1.08786 -1.67474

C 0.25972 0.03025 -0.23611

C -2.73205 0.98572 0.09707

C -4.09709 1.1922 0.26486

C -5.03531 0.22018 -0.05727

C -4.57426 -0.9948 -0.54801

C -3.217 -1.22889 -0.71787

C -2.27072 -0.24091 -0.40093

H -2.03806 1.77115 0.34469

H -6.0936 0.41447 0.04014

H -2.90295 -2.18378 -1.12204

N -0.92225 -0.55008 -0.67028

S 0.33444 1.23285 1.01256

H -0.81622 -1.3929 -1.23506

F -5.79041 -2.88065 0.25434

F -5.05699 -2.91949 -1.82873

F -6.75108 -1.57598 -1.24681

F -3.69111 3.50442 0.54004

F -5.8118 2.80855 0.3756

F -4.65299 2.43695 2.21884

C -5.54204 -2.08849 -0.84265

C -4.55983 2.48514 0.84774

**IM1**

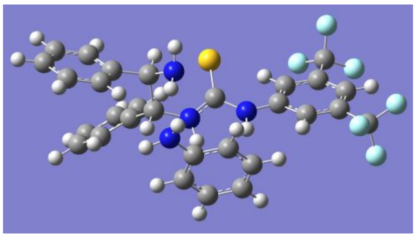

Temperature = 298.15 Kelvin

Pressure = 1 atm

Frequencies scaled by = 1

Electronic Energy (EE) = -2324.4712 Hartree

Zero-point Energy Correction = 0.509584 Hartree

Thermal Correction to Energy = 0.546307 Hartree

Thermal Correction to Enthalpy = 0.547251 Hartree

Thermal Correction to Free Energy = 0.432235 Hartree

EE + Zero-point Energy = -2323.9617 Hartree

EE + Thermal Energy Correction = -2323.9249 Hartree

EE + Thermal Enthalpy Correction = -2323.924 Hartree

EE + Thermal Free Energy Correction = -2324.039 Hartree

E (Thermal) = 342.813 kcal/mol

Heat Capacity (Cv) = 138.761 cal/mol-kelvin

Entropy (S) = 242.071 cal/mol-kelvin

Symbolic Z-matrix:

Charge = 0 Multiplicity = 1

C -4.83039 -0.81319 -1.28774

C -3.69273 -1.62221 -1.26071

C -3.83122 -3.00286 -1.41689

C -5.08988 -3.56401 -1.60258

C -6.21882 -2.75275 -1.6339

C -6.08692 -1.37739 -1.47466

H -4.74563 0.2764 -1.14978

H -2.94467 -3.64871 -1.38524

H -5.19033 -4.64791 -1.72264

H -7.20948 -3.1954 -1.78056

H -6.97444 -0.73615 -1.49361

C -2.6136 -1.09922 1.44711

C -3.59292 -0.55273 2.28254

C -4.04725 -1.26106 3.38758

C -3.52754 -2.52028 3.67038

C -2.5512 -3.06573 2.84507

C -2.09337 -2.36036 1.73687

H -4.01394 0.44231 2.064

H -4.81636 -0.8273 4.03495

H -3.88668 -3.07861 4.54106

H -2.13571 -4.05489 3.06519

H -1.30264 -2.78657 1.09179

C -2.31119 -1.04177 -1.09432

C -2.16267 -0.29464 0.25464

N -0.78877 0.25616 0.48452

N -1.96583 -0.09995 -2.19643

H -2.80021 0.63465 0.20993

H -1.5455 -1.87469 -1.1158

H -2.74473 0.49833 -2.39243

H -1.74223 -0.62264 -3.01635

H -0.80355 1.25342 0.5493

C 0.44355 -0.34233 0.25919

C 3.36423 -0.94085 -0.10192

C 4.72547 -1.12503 -0.30166

C 5.62598 -0.06793 -0.18232

C 5.13695 1.1941 0.14238

C 3.77638 1.41124 0.34909

C 2.88858 0.33616 0.22652

H 2.55295 -1.77018 -0.19674

H 6.70098 -0.22581 -0.34178

H 3.43095 2.42266 0.60449

N 1.49352 0.57396 0.48361

S 0.69641 -1.9675 -0.1082

H 1.25958 1.5447 0.53755

F 7.38282 2.14458 0.06782

F 6.03681 2.96399 1.51247

F 5.77898 3.40944 -0.56226

F 4.75369 -2.96126 -1.86786

F 6.54711 -2.68331 -0.74227

F 4.81239 -3.48637 0.20748

C 6.07315 2.39461 0.28473

C 5.20446 -2.532 -0.66668

H -3.82289 3.06635 -2.99959

C -3.81283 4.00857 -1.21256

C -3.43189 5.19639 -0.5705

C -4.12707 2.87657 -0.44767

C -3.37625 5.24325 0.81579

H -3.18461 6.09185 -1.15331

C -4.06291 2.93978 0.93844

H -4.43787 1.93764 -0.93502

C -3.68892 4.1185 1.57354

H -3.08618 6.17355 1.31579

H -4.31159 2.04764 1.53475

H -3.64578 4.16345 2.66639

N -3.97838 3.97844 -2.63067

H -3.4072 4.6551 -3.0855

**TS1**

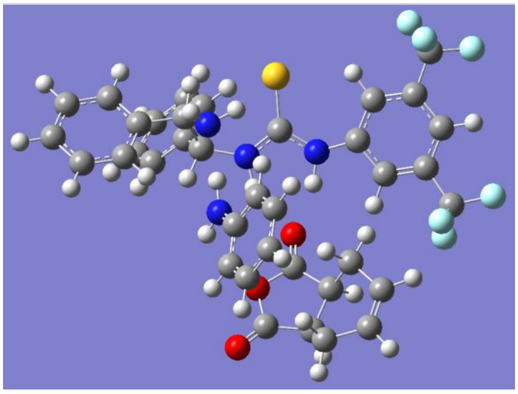

Temperature = 298.15 Kelvin

Pressure = 1 atm

Frequencies scaled by = 1

Electronic Energy (EE) = -2856.9231 Hartree

Zero-point Energy Correction = 0.663902 Hartree

Thermal Correction to Energy = 0.710089 Hartree

Thermal Correction to Enthalpy = 0.711034 Hartree

Thermal Correction to Free Energy = 0.577173 Hartree

EE + Zero-point Energy = -2856.2592 Hartree

EE + Thermal Energy Correction = -2856.213 Hartree

EE + Thermal Enthalpy Correction = -2856.212 Hartree

EE + Thermal Free Energy Correction = -2856.3459 Hartree

E (Thermal) = 445.588 kcal/mol

Heat Capacity (Cv) = 175.481 cal/mol-kelvin

Entropy (S) = 281.734 cal/mol-kelvinSymbolic Z-matrix:

Symbolic Z-matrix:

Charge = 0 Multiplicity = 1

C -4.9596 1.23218 -1.29662

C -4.91491 -0.16831 -1.20893

C -6.10819 -0.89532 -1.29117

C -7.32865 -0.23833 -1.45951

C -7.3674 1.15463 -1.55

C -6.18038 1.88726 -1.46804

H -4.0394 1.80355 -1.23715

H -6.07961 -1.97647 -1.21633

H -8.24543 -0.8131 -1.52187

H -8.31394 1.66519 -1.68518

H -6.20406 2.96867 -1.54121

C -4.06016 -1.6199 1.40973

C -5.06106 -0.98089 2.1537

C -5.90358 -1.71301 2.99217

C -5.7512 -3.0965 3.09884

C -4.75577 -3.74069 2.3598

C -3.9151 -3.01014 1.51838

H -5.18788 0.09294 2.06819

H -6.67387 -1.20373 3.55976

H -6.40167 -3.6675 3.75141

H -4.63185 -4.81487 2.43698

H -3.14753 -3.51058 0.94213

C -3.58905 -0.88824 -1.01298

C -3.17243 -0.78448 0.49263

N -1.71588 -0.97776 0.74263

N -2.52635 -0.21955 -1.83638

H -3.32992 0.26553 0.75717

H -3.69514 -1.94513 -1.27154

H -2.91713 0.03404 -2.75243

H -1.7568 -0.89068 -1.97376

H -1.30296 -0.21112 1.28027

C -0.83885 -1.89822 0.26665

C 1.98788 -3.33656 -0.30694

C 3.31974 -3.65547 -0.5702

C 4.37615 -2.83773 -0.18274

C 4.07138 -1.66848 0.50673

C 2.7612 -1.34507 0.8038

C 1.69112 -2.15486 0.38585

H 1.19551 -3.99322 -0.62855

H 5.39941 -3.11523 -0.39186

H 2.54956 -0.44758 1.36479

N 0.43251 -1.64936 0.72852

S -1.23365 -3.19034 -0.83363

H 0.51745 -0.79453 1.2897

F 6.33792 -1.2371 1.05682

F 4.764 0.11041 1.91351

F 5.2883 0.22754 -0.23073

F 3.62928 -4.64305 -2.71615

F 4.85477 -5.38706 -1.03559

F 2.66915 -5.85633 -1.13944

C 5.11881 -0.66435 0.82321

C 3.61448 -4.88558 -1.36148

C 0.89461 2.05762 1.7531

C 2.25334 4.05601 1.86378

C 0.77069 4.37375 1.96406

O 0.4729 0.90568 1.78997

H -1.82773 1.33183 -1.04632

O 0.0259 3.12325 1.93155

C 2.31467 2.54673 1.53031

O 0.18071 5.42082 2.03873

C 2.79805 2.30879 0.05608

C 2.93306 4.98035 0.79884

C 4.12008 3.03659 -0.09848

C 4.18903 4.31583 0.27184

H 3.00145 1.99476 2.17801

H 2.70945 4.24729 2.84176

H 2.22658 5.1267 -0.02861

H 3.13663 5.95551 1.24682

H 2.93154 1.24211 -0.12597

H 2.04784 2.69438 -0.6457

H 4.97076 2.46906 -0.44975

H 5.10576 4.89129 0.20987

C -0.75253 3.10696 -1.43966

C -0.53896 4.45738 -1.07306

C -0.1339 2.65011 -2.6292

C 0.25089 5.29771 -1.85725

H -1.01111 4.8486 -0.17944

C 0.65174 3.49925 -3.4024

H -0.28421 1.62001 -2.93158

C 0.85906 4.8308 -3.02639

H 0.37489 6.33342 -1.5571

H 1.10969 3.11621 -4.30808

H 1.46893 5.48786 -3.63372

N -1.5178 2.25628 -0.6759

H -1.85676 2.58619 0.22088

**TS2**

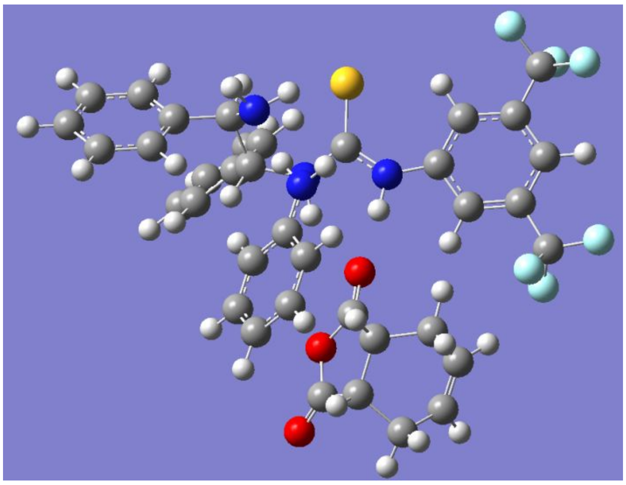

Temperature = 298.15 Kelvin

Pressure = 1 atm

Frequencies scaled by = 1

Electronic Energy (EE) = -2856.925 Hartree

Zero-point Energy Correction = 0.663878 Hartree

Thermal Correction to Energy = 0.710086 Hartree

Thermal Correction to Enthalpy = 0.71103 Hartree

Thermal Correction to Free Energy = 0.576382 Hartree

EE + Zero-point Energy = -2856.2611 Hartree

EE + Thermal Energy Correction = -2856.2149 Hartree

EE + Thermal Enthalpy Correction = -2856.214 Hartree

EE + Thermal Free Energy Correction = -2856.3486 Hartree

E (Thermal) = 445.586 kcal/mol

Heat Capacity (Cv) = 175.195 cal/mol-kelvin

Entropy (S) = 283.392 cal/mol-kelvin

Symbolic Z-matrix:

Charge = 0 Multiplicity = 1

C -5.2217 -0.35966 -1.71743

C -4.86799 -1.52275 -1.01664

C -5.87831 -2.32508 -0.47299

C -7.22079 -1.97304 -0.62366

C -7.56732 -0.81421 -1.32205

C -6.56473 -0.00953 -1.86876

H -4.44677 0.25978 -2.15335

H -5.61034 -3.22106 0.07507

H -7.99333 -2.60286 -0.19796

H -8.60961 -0.54179 -1.4414

H -6.82705 0.88978 -2.41429

C -3.4117 -1.61164 1.71615

C -4.3922 -0.85471 2.36962

C -4.94275 -1.29303 3.57564

C -4.5142 -2.4944 4.14264

C -3.53504 -3.25403 3.49661

C -2.98522 -2.81773 2.29041

H -4.73328 0.0758 1.92875

H -5.70121 -0.69686 4.06967

H -4.93769 -2.83533 5.08038

H -3.19632 -4.18691 3.93262

H -2.22495 -3.40374 1.78849

C -3.4046 -1.90049 -0.83303

C -2.83564 -1.11537 0.39419

N -1.35132 -0.97988 0.43844

N -2.63092 -1.50759 -2.05684

H -3.17399 -0.08597 0.25023

H -3.31807 -2.97133 -0.62958

H -3.16163 -1.80069 -2.88829

H -1.73788 -2.02295 -2.04328

H -1.05133 -0.05173 0.74932

C -0.35515 -1.8557 0.15028

C 3.17113 -0.73344 0.32586

C 4.51439 -1.00229 0.12516

C 4.9392 -2.2594 -0.28585

C 3.96936 -3.2372 -0.48237

C 2.60985 -2.9847 -0.32099

C 2.18778 -1.70853 0.08362

H 2.86519 0.24495 0.67307

H 5.98466 -2.46644 -0.459

H 1.88507 -3.7555 -0.52825

N 0.87543 -1.25464 0.25175

S -0.60075 -3.51717 -0.32511

H 0.84219 -0.24392 0.43745

F 4.62744 -5.40133 0.26671

F 5.58061 -4.58935 -1.55313

F 3.44577 -5.25783 -1.59425

F 5.34629 0.71586 1.54083

F 6.77064 -0.27367 0.13163

F 5.21388 1.14027 -0.61023

C 4.40112 -4.61873 -0.84229

C 5.47212 0.1202 0.29277

C 0.61929 2.68239 0.72385

C 1.20106 4.99696 0.32574

C 0.26556 4.83279 1.51475

O 0.55609 1.4576 0.74718

H -2.1059 0.24577 -2.4628

O -0.04948 3.43197 1.67386

C 1.35984 3.57798 -0.26199

O -0.17388 5.64914 2.28457

C 2.83861 3.13428 -0.42139

C 2.53292 5.63908 0.8247

C 3.59824 3.41767 0.86155

C 3.43136 4.59834 1.46206

H 3.27442 3.70292 -1.25474

H 2.89717 2.0789 -0.69155

H 3.03988 6.08775 -0.04062

H 2.2841 6.44569 1.52089

H 4.25937 2.65359 1.24874

H 3.94632 4.84857 2.38328

H 0.8233 3.49703 -1.21632

H 0.70764 5.66417 -0.3853

C -1.81 2.36176 -2.37306

C -1.34079 3.50397 -3.07361

C -2.31463 2.57834 -1.06535

C -1.41693 4.77761 -2.51607

H -0.94684 3.37825 -4.07703

C -2.37845 3.854 -0.51428

H -2.68839 1.73199 -0.5034

C -1.93905 4.97346 -1.23182

H -1.07787 5.62845 -3.09826

H -2.78508 3.9769 0.48348

H -2.02499 5.96821 -0.81147

N -1.78096 1.11687 -2.93446

H -1.44167 1.02581 -3.88597

**IM2**

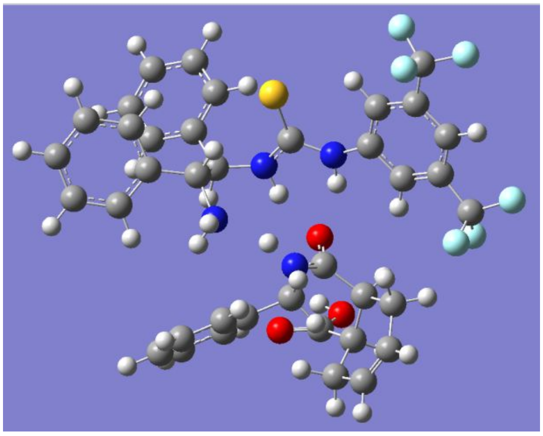

Temperature = 298.15 Kelvin

Pressure = 1 atm

Frequencies scaled by = 1

Electronic Energy (EE) = -2856.9079 Hartree

Zero-point Energy Correction = 0.665091 Hartree

Thermal Correction to Energy = 0.710614 Hartree

Thermal Correction to Enthalpy = 0.711559 Hartree

Thermal Correction to Free Energy = 0.57868 Hartree

EE + Zero-point Energy = -2856.2428 Hartree

EE + Thermal Energy Correction = -2856.1973 Hartree

EE + Thermal Enthalpy Correction = -2856.1964 Hartree

EE + Thermal Free Energy Correction = -2856.3292 Hartree

E (Thermal) = 445.917 kcal/mol

Heat Capacity (Cv) = 173.503 cal/mol-kelvin

Entropy (S) = 279.667 cal/mol-kelvinSymbolic Z-matrix:

Symbolic Z-matrix:

Charge = 0 Multiplicity = 1

C -4.99221 -0.37844 -1.76649

C -4.02276 -1.31088 -1.37765

C -4.28088 -2.68563 -1.49769

C -5.50568 -3.12371 -2.03492

C -6.46171 -2.18678 -2.45622

C -6.20716 -0.81442 -2.31119

H -4.79766 0.6636 -1.64833

H -3.55118 -3.40077 -1.18409

H -5.70441 -4.16692 -2.12634

H -7.37978 -2.51618 -2.88361

H -6.9397 -0.09663 -2.62176

C -3.31841 -2.06641 1.33754

C -4.70396 -2.21774 1.50535

C -5.22132 -3.36426 2.12125

C -4.35669 -4.37323 2.57045

C -2.96857 -4.24497 2.37436

C -2.44885 -3.09141 1.74796

H -5.35762 -1.45029 1.1701

H -6.27683 -3.46695 2.25142

H -4.75216 -5.23318 3.06358

H -2.31409 -5.01994 2.70316

H -1.39496 -3.00109 1.59898

C -2.67807 -0.81347 -0.81175

C -2.79165 -0.75541 0.72155

N -1.48584 -0.49687 1.21698

N -2.45258 0.56304 -1.40269

H -3.47539 0.02341 0.98911

H -1.89655 -1.48536 -1.09017

H -3.2181 1.12702 -1.09064

H -2.47068 0.45225 -2.40061

H -1.11616 0.3681 1.27892

C -0.54998 -1.44872 1.62528

C 1.89376 -2.65306 0.66201

C 2.96933 -3.07518 -0.13265

C 4.11338 -2.27911 -0.22697

C 4.17097 -1.0505 0.43415

C 3.11248 -0.61632 1.17185

C 1.94895 -1.40404 1.29554

H 1.03011 -3.26884 0.76856

H 4.95309 -2.61093 -0.80896

H 3.15917 0.3424 1.66157

N 0.79734 -0.85355 2.03951

S -0.88368 -3.20057 1.65991

H 0.67625 0.0681 1.81391

F 6.5117 -0.98375 0.15155

F 5.59138 0.55599 1.43688

F 5.32612 0.64082 -0.74983

F 2.33273 -4.17935 -2.12458

F 4.12251 -4.91569 -1.0704

F 2.11718 -5.27737 -0.22605

C 5.43934 -0.18142 0.31434

C 2.88561 -4.40296 -0.91014

C 0.33614 1.85822 0.4999

C 1.32262 4.07551 1.12397

C 0.7815 4.04811 2.56698

O -0.07778 1.19849 1.51533

H -1.16141 1.36771 -1.0865

O 1.67767 4.15976 3.67259

C 1.64746 2.64879 0.63303

O -0.45816 3.93197 2.77512

C 2.3499 2.75982 -0.73493

C 0.23615 4.66628 0.20271

C 1.52214 3.61988 -1.71718

C 0.66411 4.58889 -1.27391

H 2.2997 2.15431 1.32195

H 2.21209 4.67262 1.09848

H -0.00716 4.15747 0.49124

H 0.04946 5.68243 0.47621

H 3.29632 3.23816 -0.59444

H 2.49643 1.78043 -1.13931

H 1.63221 3.4648 -2.76811

H 0.27382 5.29916 -1.97112

C -0.75977 3.35515 -0.95393

C -1.96009 3.9497 -0.02121

C -0.60289 4.0373 -2.17513

C -1.66622 5.37258 0.04922

H -2.1023 3.42307 0.89784

C -0.95431 5.39076 -2.27227

H -0.21069 3.52186 -3.02734

C -1.47591 6.05859 -1.15307

H -2.07738 5.88149 0.89284

H -0.82286 5.91137 -3.19181

H -1.72756 7.09851 -1.22697

N -0.3205 1.9549 -0.8392

H 1.33319 3.6617 4.41478

**Product**

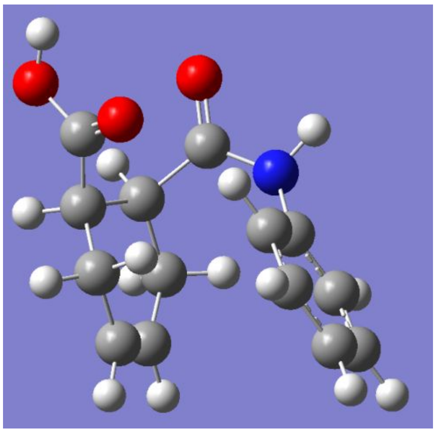

Thermo Tab Data Section:

Imaginary Freq = 0

Temperature = 298.15 Kelvin

Pressure = 1 atm

Frequencies scaled by = 1

Electronic Energy (EE) = -857.52876 Hartree

Zero-point Energy Correction = 0.300588 Hartree

Thermal Correction to Energy = 0.317252 Hartree

Thermal Correction to Enthalpy = 0.318196 Hartree

Thermal Correction to Free Energy = 0.255861 Hartree

EE + Zero-point Energy = -857.22817 Hartree

EE + Thermal Energy Correction = -857.21151 Hartree

EE + Thermal Enthalpy Correction = -857.21057 Hartree

EE + Thermal Free Energy Correction = -857.2729 Hartree

E (Thermal) = 199.079 kcal/mol

Heat Capacity (Cv) = 64.889 cal/mol-kelvin

Entropy (S) = 131.194 cal/mol-kelvin

Symbolic Z-matrix:

Charge = 0 Multiplicity = 1

C -1.00462 -0.66972 1.4066

C -1.76639 0.83877 -0.58676

C -2.39289 -0.48652 -0.96406

O -1.92481 -1.41181 1.79338

H 0.35331 -2.01015 1.96363

O -3.72956 -0.49804 -0.65683

C -1.36483 0.75669 0.93734

O -1.80764 -1.4566 -1.4435

C -0.39098 1.88186 1.38749

C -0.60907 1.17588 -1.54162

C 0.37567 2.57185 0.28344

C 0.27751 2.27299 -1.00995

H -2.31305 0.88948 1.4654

H -2.53713 1.60964 -0.66048

H -0.01211 0.27728 -1.71196

H -1.03132 1.47082 -2.51109

H -0.96567 2.64029 1.93785

H 0.31987 1.45427 2.10487

H 1.046 3.36438 0.60738

H 0.87582 2.81209 -1.73908

C 1.43217 -0.81603 0.63772

C 1.42447 -1.27877 -0.68425

C 2.57807 -0.20748 1.15631

C 2.55238 -1.1011 -1.48799

H 0.52459 -1.74619 -1.0685

C 3.70571 -0.0374 0.3525

H 2.57697 0.1233 2.18762

C 3.69321 -0.4819 -0.97192

H 2.54198 -1.4547 -2.51259

H 4.59112 0.43792 0.75818

H 4.56967 -0.35174 -1.59605

N 0.31065 -1.09672 1.50425

H -4.08096 -1.42274 -0.78844

Endo form(3a)

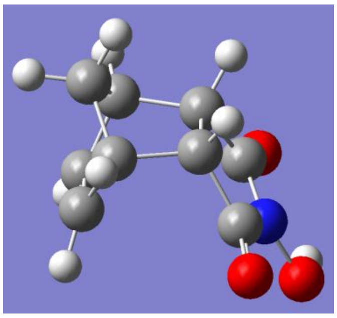

Thermo Tab Data Section:

Imaginary Freq = 0

Temperature = 298.15 Kelvin

Pressure = 1 atm

Frequencies scaled by = 1

Electronic Energy (EE) = -628.90838 Hartree

Zero-point Energy Correction = 0.17153 Hartree

Thermal Correction to Energy = 0.181327 Hartree

Thermal Correction to Enthalpy = 0.182271 Hartree

Thermal Correction to Free Energy = 0.136334 Hartree

EE + Zero-point Energy = -628.73685 Hartree

EE + Thermal Energy Correction = -628.72705 Hartree

EE + Thermal Enthalpy Correction = -628.72611 Hartree

EE + Thermal Free Energy Correction = -628.77204 Hartree

E (Thermal) = 113.784 kcal/mol

Heat Capacity (Cv) = 39.86 cal/mol-kelvin

Entropy (S) = 96.682 cal/mol-kelvin

Symbolic Z-matrix:

Charge = 0 Multiplicity = 1

C 1.40628 -0.61069 1.38982

C 1.69965 -1.08726 -0.02561

C 1.63357 1.17661 -0.05925

C 1.3679 0.72656 1.37003

H 1.17226 -1.26154 2.22207

H 1.09793 1.38741 2.18341

C 2.61051 0.06515 -0.51895

H 2.78631 0.05407 -1.59886

H 3.56431 0.10183 0.0094

C 0.42092 -0.78573 -0.8953

H 0.52389 -1.22813 -1.88803

C 0.37551 0.77069 -0.91916

H 0.45 1.19108 -1.92418

H 2.065 -2.10652 -0.12944

H 1.93813 2.21202 -0.19561

C -0.9028 -1.23997 -0.29642

C -0.97104 1.12648 -0.33435

N -1.62081 -0.06331 -0.06674

O -1.28729 -2.3547 -0.05828

O -1.48044 2.20494 -0.10146

O -2.86359 -0.05729 0.52325

H -3.04446 0.89826 0.6155

**Exo form(3a)**

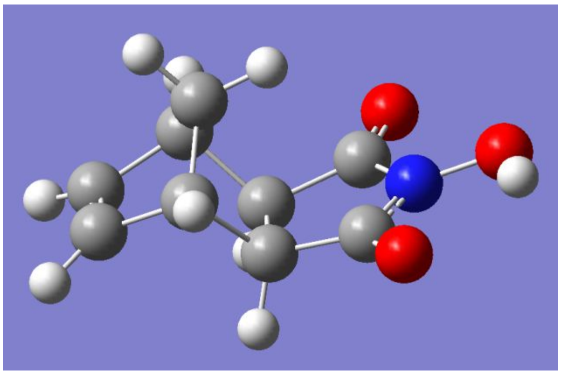

Thermo Tab Data Section:

Imaginary Freq = 0

Temperature = 298.15 Kelvin

Pressure = 1 atm

Frequencies scaled by = 1

Electronic Energy (EE) = -628.90929 Hartree

Zero-point Energy Correction = 0.171537 Hartree

Thermal Correction to Energy = 0.181365 Hartree

Thermal Correction to Enthalpy = 0.182309 Hartree

Thermal Correction to Free Energy = 0.136295 Hartree

EE + Zero-point Energy = -628.73776 Hartree

EE + Thermal Energy Correction = -628.72793 Hartree

EE + Thermal Enthalpy Correction = -628.72699 Hartree

EE + Thermal Free Energy Correction = -628.773 Hartree

E (Thermal) = 113.808 kcal/mol

Heat Capacity (Cv) = 39.918 cal/mol-kelvin

Entropy (S) = 96.845 cal/mol-kelvin

Symbolic Z-matrix:

Charge = 0 Multiplicity = 1

C -2.76444 -0.7265 -0.28635

C -1.44997 -1.16014 0.35188

C -1.50259 1.10104 0.36033

C -2.79508 0.61085 -0.28161

H -3.47973 -1.39999 -0.74084

H -3.54131 1.25323 -0.7314

C -1.26811 -0.0287 1.39173

H -0.2834 -0.00622 1.86619

H -2.03641 -0.05018 2.16485

C -0.34357 -0.77676 -0.70151

C -0.37719 0.78177 -0.69178

H -1.38557 -2.18929 0.69918

H -1.48658 2.12892 0.71639

C 1.06259 -1.13279 -0.27065

C 1.0119 1.23001 -0.24783

N 1.74676 0.05308 -0.08369

O 1.42227 2.34505 -0.06149

O 1.58527 -2.21372 -0.08495

O 3.04733 0.04446 0.36721

H 3.23714 -0.91132 0.43538

H -0.54038 -1.22849 -1.67438

H -0.59136 1.2328 -1.66108

2step TNTU

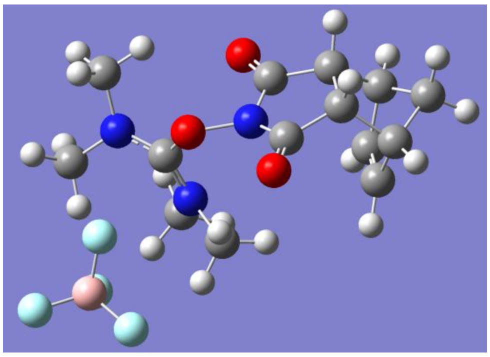

Calculation Method = RB3LYP

Basis Set = Aug-CC-pVTZ

Charge = 0

Spin = Singlet

Solvation = scrf=(cpcm,solvent=dmso)

E(RB3LYP) = -1360.3748 Hartree

RMS Gradient Norm = Hartree/Bohr

Imaginary Freq =

Dipole Moment = 19.441851 Debye

Point Group = C1

Symbolic Z-matrix:

Charge = 0 Multiplicity = 1

C -3.2963 -0.58882 1.71334

C -4.233 0.3289 0.9428

C -4.02172 -1.59492 -0.23776

C -3.17395 -1.72529 1.01789

H -2.77426 -0.31323 2.62047

H -2.53421 -2.56911 1.24015

C -5.18108 -0.72146 0.3078

H -5.83117 -0.31414 -0.47203

H -5.78242 -1.24427 1.05234

C -3.48625 0.79484 -0.36565

H -4.07969 1.54982 -0.886

C -3.33339 -0.52761 -1.17186

H -3.83435 -0.49776 -2.14174

H -4.68069 1.14835 1.50062

H -4.27685 -2.51913 -0.75086

C -2.08444 1.3522 -0.21043

C -1.84474 -0.69893 -1.4351

O -1.71976 2.3969 0.26637

O -1.27234 -1.53948 -2.06492

C 0.97742 0.96766 -0.00909

N 0.9697 0.10471 0.989

N 1.71382 2.06326 -0.10541

C 0.74287 -1.33505 0.76158

C 3.06516 2.17011 0.47207

C 1.33474 3.16109 -1.00758

C 1.3894 0.46746 2.34751

H 0.25536 3.21062 -1.11083

H 1.69303 4.09051 -0.56327

H 1.80336 3.02701 -1.98528

H 3.0779 2.94045 1.24765

H 3.4018 1.21265 0.85618

H 3.74225 2.45831 -0.33385

H 0.6345 -1.53922 -0.29909

H 1.62437 -1.87204 1.11206

H -0.14552 -1.65376 1.31008

H 1.34015 1.54643 2.47896

H 0.68861 -0.00123 3.04205

H 2.39674 0.09834 2.54342

B 4.04012 -1.29046 -0.18835

F 3.9722 -0.75849 1.15364

F 5.33893 -1.21334 -0.65978

F 3.17258 -0.47566 -0.98875

F 3.54814 -2.60024 -0.16428

O 0.18317 0.76131 -1.10116

N -1.23538 0.38521 -0.76108
